# The human endogenous attentional control network includes a ventro-temporal cortical node

**DOI:** 10.1038/s41467-020-20583-5

**Published:** 2021-01-15

**Authors:** Ilaria Sani, Heiko Stemmann, Bradley Caron, Daniel Bullock, Torsten Stemmler, Manfred Fahle, Franco Pestilli, Winrich A. Freiwald

**Affiliations:** 1 Laboratory of Neural Systems, The Rockefeller University, 1230 York Avenue, New York, NY 10065 USA; 2 Laboratory of Neurology & Imaging of Cognition, University of Geneva, Chemin de mines 9, 1202 Geneva, CH Switzerland; 3 Institute for Brain Research and Center for Advanced Imaging, University of Bremen, 28334 Bremen, Germany; 4 Department of Psychological and Brain Sciences, Indiana University, Bloomington, IN USA; 5 Department of Psychology, The University of Texas at Austin, Austin, TX 78712 USA; 6 Center for Brains, Minds & Machines, Cambridge, MA USA

**Keywords:** Attention, Brain

## Abstract

Endogenous attention is the cognitive function that selects the relevant pieces of sensory information to achieve goals and it is known to be controlled by dorsal fronto-parietal brain areas. Here we expand this notion by identifying a control attention area located in the temporal lobe. By combining a demanding behavioral paradigm with functional neuroimaging and diffusion tractography, we show that like fronto-parietal attentional areas, the human posterior inferotemporal cortex exhibits significant attentional modulatory activity. This area is functionally distinct from surrounding cortical areas, and is directly connected to parietal and frontal attentional regions. These results show that attentional control spans three cortical lobes and overarches large distances through fiber pathways that run orthogonally to the dominant anterior-posterior axes of sensory processing, thus suggesting a different organizing principle for cognitive control.

## Abstract (web summary)

Endogenous attention is known to be controlled by dorsal fronto-parietal brain areas. Here the authors identify a control attention area located in the temporal lobe, which is functionally distinct from surrounding areas, and is directly connected to parietal and frontal attentional regions.

## Introduction

Endogenous attention is the brain function that supports goal-directed behavior by selecting currently relevant pieces of information at the expense of irrelevant ones^1^. The dominant neurocognitive model of this process suggests that frontal and parietal areas form a network that controls the focus of attention. This model is supported by a large body of literature, across a wide range of research domains, including neuropsychology^2–7^, whole-brain functional magnetic resonance imaging (fMRI) across primate species^8–12^, and electrophysiology in macaque monkeys^13–15^.

**Fig. 1:**
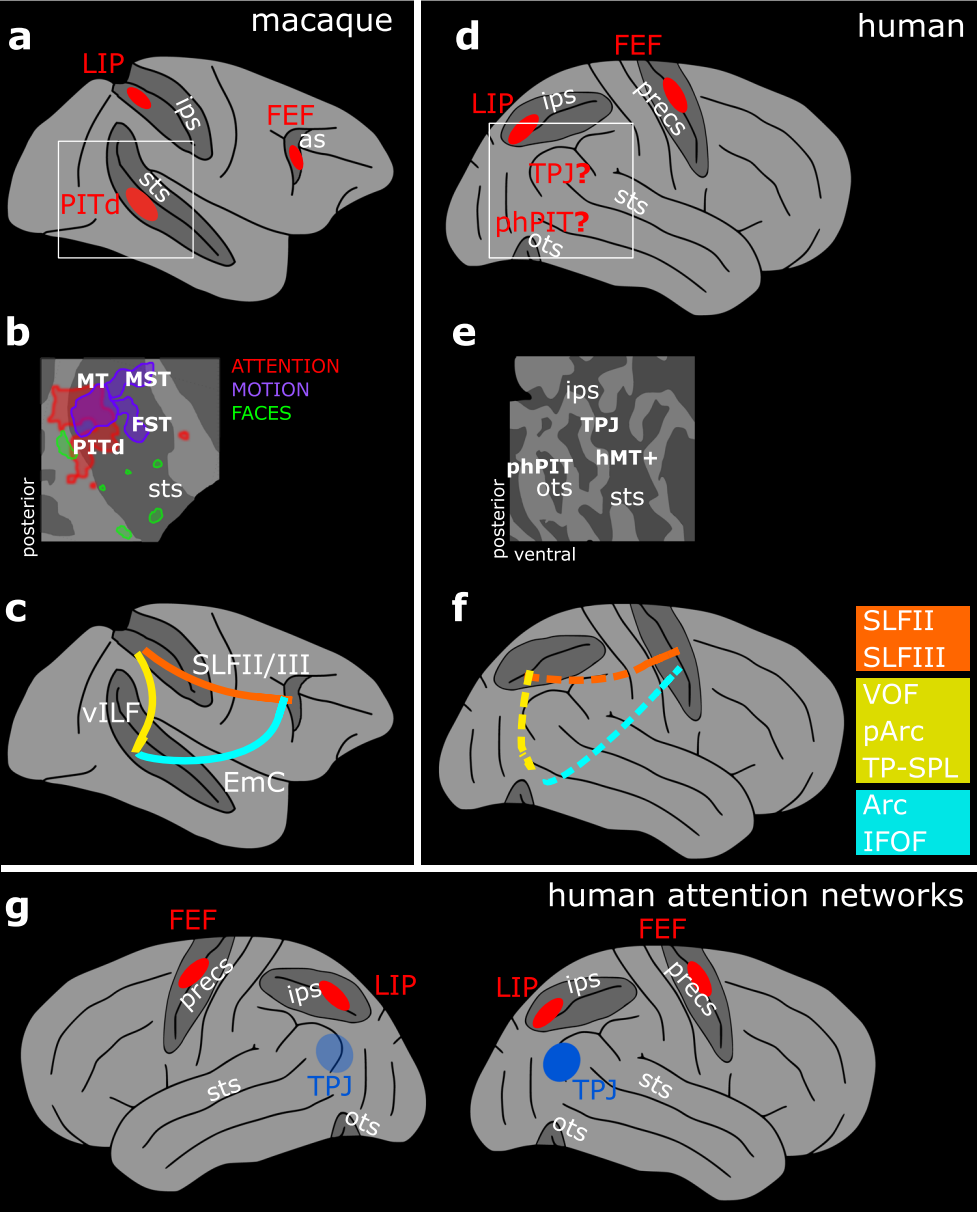
Functional and structural identification of the putative ventral endogenous attention node. **a** Whole brain model of the three-node attention network of the macaque as defined by functional activation in ref. ^10^. **b** Functional characterization of the macaque ventral attention node and nearby areas displayed on a schematic flat map of the right superior temporal sulcus^10^. PITd was activated by attention, but not by motion – the task relevant dimension. **c** Schematic of macaque PITd connections with the dorsal attention network as defined in ref. ^19^. **d** Whole brain model of the human fronto-parietal attention network and the two candidate areas possibly constituting a human ventral node for endogenous attention. **e** Schematic flat map of the human superior temporal sulcus and the quest for a functional characterization of parieto-temporal areas around phPIT and TPJ. **f** Schematic of the hypothesized connections between the putative ventral attention node and the dorsal attention network; colored squares indicate possible alternative hypothesis for the homolog fibers bundles in humans and macaques. **g** Schematic of the components of the endogenous (red) and exogenous (blue) attention network in the human brain; neuroimaging data suggest stronger activation in the right hemisphere for the latter (opaque vs. transparent blue). as arcuate sulcus, ips intraparietal sulcus, ots occipito-temporal sulcus, precs pre-central sulcus, sts superior temporal sulcus, FEF Frontal Eye Field, FST fundus of the superior temporal sulcus, LIP Lateral Intraparietal area, MT and MT+ middle temporal area, MST medial superior temporal area, PITd Posterior Infero-Temporal dorsal area, phPIT putative human Posterior Infero-Temporal area, TPJ temporo-parietal junction, Arc Arcuate Fasciculus, EmC Extreme Capsule, IFOF Inferior Frontal Occipital Fasciculus, ILF Inferior Longitudinal Fasciculus, pArc posterior Arcuate Fasciculus, SLF Superior Longitudinal Fasciculus, TP–SPL Temporo-Parietal connection to the Superior Temporal Lobule, vILF vertical branch of the Inferior Longitudinal Fasciculus, VOF Vertical Occipital Fasciculus.

Deviating from this dominant model, recent results identified an area in the lower bank of the macaque superior temporal sulcus (STS), the dorsal portion of posterior infero-temporal cortex (PITd), as an additional attentional control area (Fig. 1a, b). The designation of this area as an attentional control area is supported by several related findings. First, this area has been found to be strongly engaged by multiple attention tasks, but not by the task-relevant feature dimension (Fig. 1b)^10^, a pattern also observed subsequently in individual PITd neurons^16^. Second, the neurons encoded attentional state, yet provided little information on visual stimulus properties^16^, as would be expected for attentional control regions^17^. Third, PITd has been shown to be both necessary and sufficient for directing spatial attention^16,18^. And fourth, PITd has been shown to be interconnected with frontal and parietal attention areas (Fig. 1c)^19^. Thus area PITd exhibits the same functional characteristics as other classical attention control areas located in the parietal and frontal cortex, to which it is directly connected.

To date it is not yet known whether the human brain possesses a homolog area with similar properties to PITd. A first hypothesis is that the temporo-parietal junction (TPJ) is the homolog of macaque area PITd^18^. TPJ, like macaque PITd, is located ventrally to parietal and frontal attention control areas. TPJ supports attentional functions (Fig. 1g): while the dorsal partieto-frontal attention network controls the endogenous, goal-driven focus of attention, TPJ has been suggested to control exogenous attention^9^ or context updating^20–22^, both of which constitute forms of attention guided by external stimulation. Lesions to TPJ, similar to lesions in parietal and frontal regions^23^, have been shown to result in the emergence of spatial neglect, a neurological condition wherein patients are either found to have great difficulties or to be wholly incapable of allocating of attentional resources to the contralesional hemifield^23,24^. Similarly, inactivation of macaque area PITd^18^ and lesions of the STS^25^ also produce neglect-like spatial attention deficits. However, while macaque area PITd is strongly activated during prolonged, sustained attention^10,11^, human TPJ activity is actually reduced during endogenous attention^20,26^. And while macaque area PITd does not activate during tasks involving target detection and shifts of attention^12,27^, human TPJ activity increases in those experimental conditions, particularly when stimuli are salient or unexpected^9,28^. Furthermore, while PITd is directly connected to parietal and frontal attention control areas^19^, TPJ is thought to belong to a neuroanatomical substrate segregated from the endogenous attention network^29^. Under a homology argument, a second hypothesis appears plausible, namely the existence of an unreported ventral attentional node in the human brain^10,19^. Specifically, this node could be located  within the putative human posterior infero-temporal area (phPIT; Fig. 1d), suggested to be the corresponding location^30,31^ and the retinotopic homolog of macaque PITd^32^.

Here we directly tested whether a previously unreported ventral attentional control area exists in the human brain. Our approach consisted of three components: first, we combined the same behavioral paradigm that led to the discovery of the macaque ventral attention node with whole-brain functional magnetic resonance imaging (fMRI) to map attentional modulation and to permit a direct, inter-species comparison of attention maps (Fig. 1d). Second, we mapped visual responses of the human temporal lobe in search of a visually responsive and attentionally modulated, yet not shape or motion selective area (Fig. 1e). Third, we used diffusion-weighted MRI (dMRI) and tractography to define the structural connections of this putative attention node with traditional dorsal attention control areas (Fig. 1f). For the interpretation of human brain organization and function, we used a comparative approach that allowed us to transfer deep functional and causal knowledge from the macaque brain (that cannot be obtained in humans)^33^.

Our results provide direct evidence for the existence of a previously unrecognized attention node located in the ventral temporal cortex of the human brain. This node is functionally distinct from surrounding cortical areas and not tuned to any specific visual feature. Crucially it is directly connected to parietal and frontal attentional regions.

## Results

### A high-load attentional task reveals activation of the ventral cortical area phPIT, but not of TPJ

**Fig. 2:**
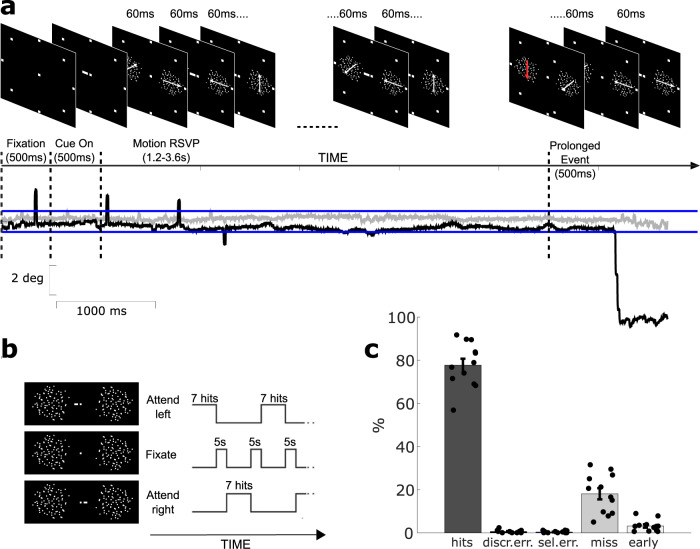
Experimental design and behavioral performance. **a** Example trial of the attentive motion discrimination task depicting critical task events. Top row shows display elements of the task; note that they are not drawn in scale (see Methods). Bottom row show an example eye trace corresponding to the trial on top: subjects were required to keep fixation until the prolonged motion event occurred (see also methods); black and gray solid traces represent *x* and *y* axes, respectively; dashed lines mark critical task events; blue lines show the tolerance window for eye movements (±1 deg. visual angle); spikes in the eye traces correspond to eye blinks and were not considered as breaks in fixation. **b** Schematic of the block design adopted during scanning. The experiment included three types of block: ‘Attend left’ and ‘Attend right’ where the task described in A was preformed; ‘Passive Fixation’ blocks where the trial structure was exactly the same as in the attention trials, but no attentional cue was displayed. Subjects alternated between paying attention to the right, passive fixation, and paying attention to the left. During the “right” and “left” blocks, subjects had to detect and discriminate a motion event at the cued location, while ignoring similar visual stimulation at the irrelevant location. During passive fixation blocks, the trial structure was the same as the attention blocks, but subjects were required to passively fixate the central spot while the moving stimuli were displayed. See also Methods. **c** Behavioral performance of human subjects. In each trial, of the 8 possible motion directions, one was chosen for the target, and a different one was chosen for the distractor. This resulted in five main behavioral outcomes: the subject could saccade into the motion direction displayed by the target (“hit”) or the direction of the distracter (“selection error”) or to 1 of 6 remaining targets (“discrimination error”); the subject could fail to respond to the prolonged event (“missed detection”) or respond before the prolonged event actually occurred (“early selection”). Data are expressed as mean across 12 subjects; gray points represent the values for each individual subject. Source data are provided as a Source Data file.

To unravel whether humans possess a ventral attention node similar to the one previously described in macaques, we had human subjects perform the same attentive motion-tracking task that lead to the discovery of PITd as an attention area in macaque monkeys^10^. The task required subjects to covertly pay attention to one of two random dot stimuli (Fig. 2a; see Methods). Random dots changed translation direction every 60 ms, until the translation direction ceased changing for 500 ms (prolonged event), which then returned once more to rapid direction changes. Subjects were required to pay attention to the cued rapid visual serial presentation (RSVP) to detect the prolonged motion event and discriminate its direction of motion by making a saccade to one out of 8 peripheral saccade target dots (Fig. 2a). Blocks of attention task trials were separated by periods of passive fixation (Fig. 2b). During scanning, human subjects detected and discriminated the prolonged motion event on the target random dot stimulus correctly in 78.3% of the trials (Fig. 2c, see Methods). They missed the target prolonged motion event in 17.6% of trials, indicating that the task was difficult and attention-demanding.

**Fig. 3:**
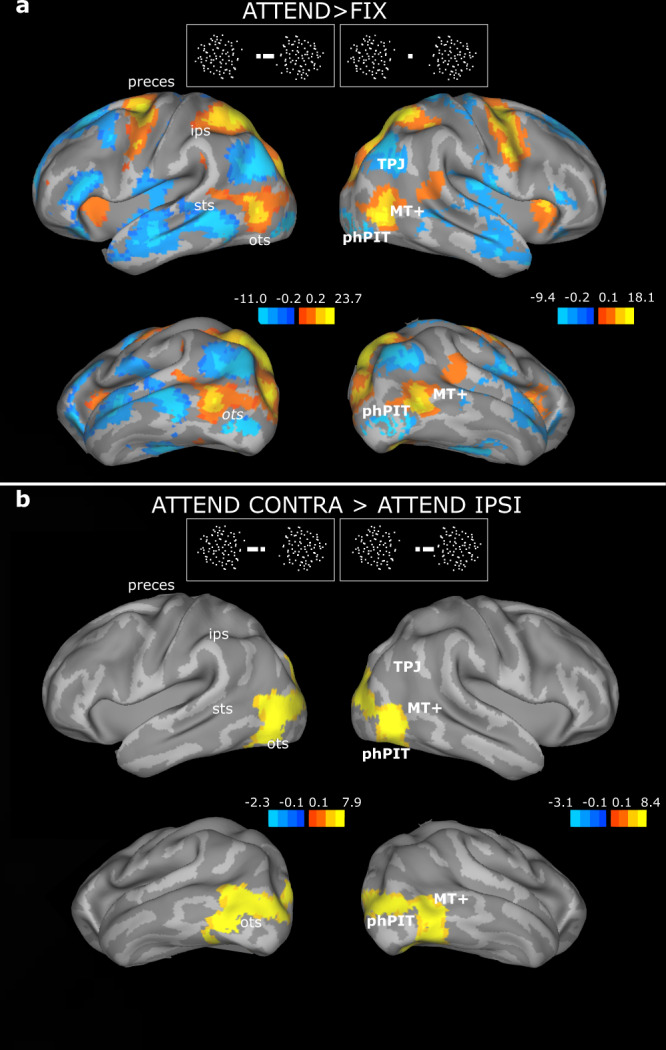
Attentive motion discrimination leads to distinct activations and inactivation in occipital, temporal, parietal, and frontal lobes. **a** Statistical parametric maps of the contrast ‘attention (ATTEND) vs. fixation (FIX)’ overlaid on the lateral and inferior views of the inflated average human brain. The color-bar shows *T*-values task-related activations (yellow/red) and inactivation (blue). **b** Statistical parametric maps of the contrast ‘attend contralaterally (CONTRA) versus ipsilaterally (IPSI)’. Conventions as in A. One GLM analysis was performed for the whole brain. The contrasts attention Right > Left and Left > Right are shown in the left and right column, respectively, i.e., attention contralateral > ipsilateral. The color-bar shows *T*-values task-related activations; for visualization purposes, a slightly different threshold is displayed for the left and right hemisphere to highlight activation similarities; the full range of the color-bars is used; dark orange shades are only visible at the edges. sts superior temporal sulcus, ips intraparietal sulcus, as arcuate sulcus, ots occipito-temporal sulcus, MT+ middle temporal area, phPIT putative human Posterior Infero-Temporal area, TPJ temporo-parietal junction.

Contrasting activation during the attentive motion discrimination task versus passive fixation allowed identification of brain regions with task-related activation in the broadest sense, i.e., related to sensory processing, attention, response generation, or interactions between these components (Fig. 3a). We compared the resulting attentional activation pattern to recent functional and anatomical maps, in particular recently defined maps of parieto-occipito-temporal areas of the human brain^31,32^. We found task-related activations in early and mid-level visual areas (including V1, V2, V3a, and V4v), the middle temporal MT+ motion complex, object-selective, but not face-selective or scene-selective areas, and the posterior tip of the occipito-temporal sulcus (OTS). More dorsally, significant activation was observed in the superior parietal lobule, the intraparietal sulcus (including the lateral intra-parietal area, LIP), and the precentral gyrus (Brodmann 6 and 8, including the frontal eye fields, FEF). The TPJ and insular regions showed decreased activation. This overall activation pattern is consistent with previous fMRI studies in humans^9,34^.

Our main goal for the analysis of brain activation patterns during the attention task was to isolate cortical areas modulated specifically by endogenous attention. We thus contrasted the two spatial attention conditions ‘attend contralateral’ and ‘attend ipsilateral’ (Fig. 3b), which differed with respect to the allocation of spatial attention but not with respect to task demand, response preparation, or execution. These spatial attention conditions were also dissociated from saccade planning, since saccades to any of the eight different targets were generated equally frequently in both conditions. Attentional modulation was localized in early retinotopic visual areas and MT+, posterior lateral occipital LO complex, phPIT and V8^31,32,35^ (see also Supplementary Table 1 and Figs. S1, S2). phPIT occupies a homologous position in retinotopic cortex to macaque PITd^32^, and here we show that it is modulated by the same attention task that also modulated macaque PITd.

### Ventral cortical area phPIT shows a visual selectivity profile distinct from that of nearby areas

**Fig. 4:**
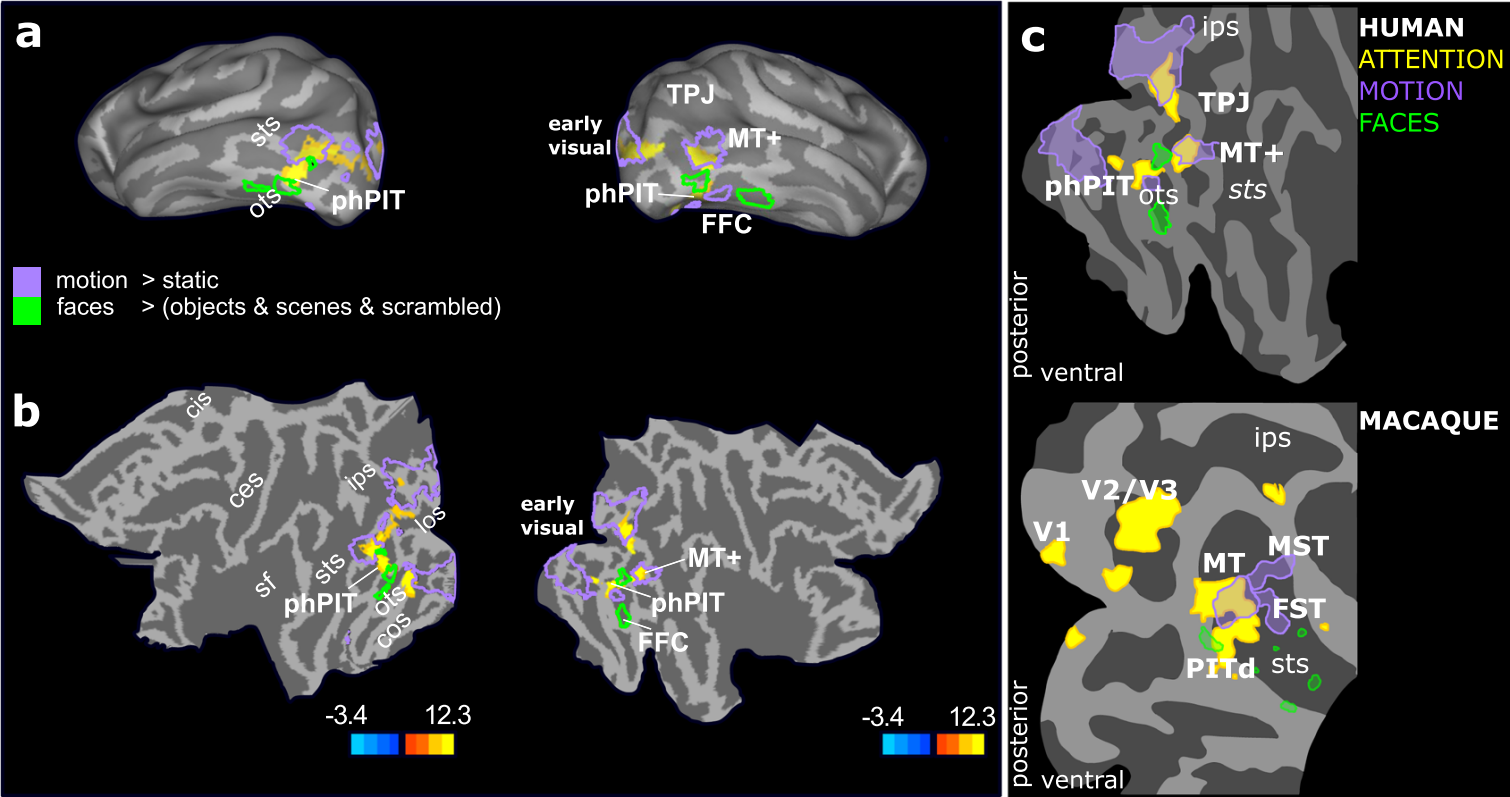
phPIT functional profile differs from nearby areas. **a** Statistical parametric maps of the contrast ‘attend contralaterally versus ipsilaterally’ overlaid on the inferior views of the average human inflated brain. Conventions as in Fig. 3a, b. Solid lines show visual selectivity for motion (purple) and faces (green). **b** Statistical parametric maps of the contrast ‘attend contralaterally versus ipsilaterally’ overlaid on flat map of the left and right hemispheres; conventions as in panel A. The color-bar shows *T*-values task-related activations. **c** Schematic flat-map representations of activation patterns in humans (top) and macaques (bottom). ces central sulcus, cos collateral sulcus, ips intraparietal sulcus, los lateral occipital sulcus, ots occipito-temporal sulcus, sf Sylvian Fissure, sts superior temporal sulcus, FFC fusiform Face Area, FST fundus of the superior temporal sulcus, LIP Lateral Intraparietal area, MT and MT+ middle temporal area, MST medial superior temporal area, PITd Posterior Infero-Temporal dorsal area, phPIT putative human Posterior Infero-Temporal area, TPJ temporo-parietal junction, V1-2-3 visual areas 1-2-3.

To gain a deeper understanding of phPIT functional specialization, we first tested whether this area is activated by attention, yet not specialized for the task relevant dimension, a key property of attentional priority maps encoding the current locus of attention^17^. Second we assessed similarities of the functional organization of cortex surrounding PITd and phPIT: macaque area PITd is surrounded by a characteristic pattern of visually specialized areas (Fig. 1b)^10^. More specifically, PITd is adjacent to motion areas (purple areas, Fig. 1b) and located in between two face patches (green areas, Fig. 1b). This spatial pattern of functional selectivity provides a unique opportunity to establish cross-species homology. We ran two separate localizers to establish motion-selectivity and face-selectivity. Figure 4a, b shows that phPIT is located directly adjacent to, but not overlapping with, motion-selective areas (purple outlines) and face selective areas (green outlines) bilaterally. Importantly, activity in phPIT was not modulated by motion, the relevant feature dimension in the attention task (Fig. 4; see also Figs. S2 and S3). Furthermore, phPIT activity was modulated by neither object shape selectivity, nor by scene selectivity (Figs. S2 and S3). The combination of retinotopic and eccentricity mapping (see methods section) allowed us to further separate the infero-temporal activation from that of early visual areas, of motion areas, and of other specialized areas located more anteriorly in the temporal lobe (Fig. S4). In individual subjects we were able to identify the infero-temporal activation as the most anterior and ventral area possessing a retinotopic organization. More specifically, the phPIT activated by attention corresponded in part to Glasser area PIT and contained the representation of intermediate and peripheral eccentricities and in part to Glasser area V8 containing the representation of central and intermediate eccentricities. These results are consistent with previous fine retinotopic mapping of this infero-temporal region (ref. ^32,35^; see also methods section).

**Fig. 5:**
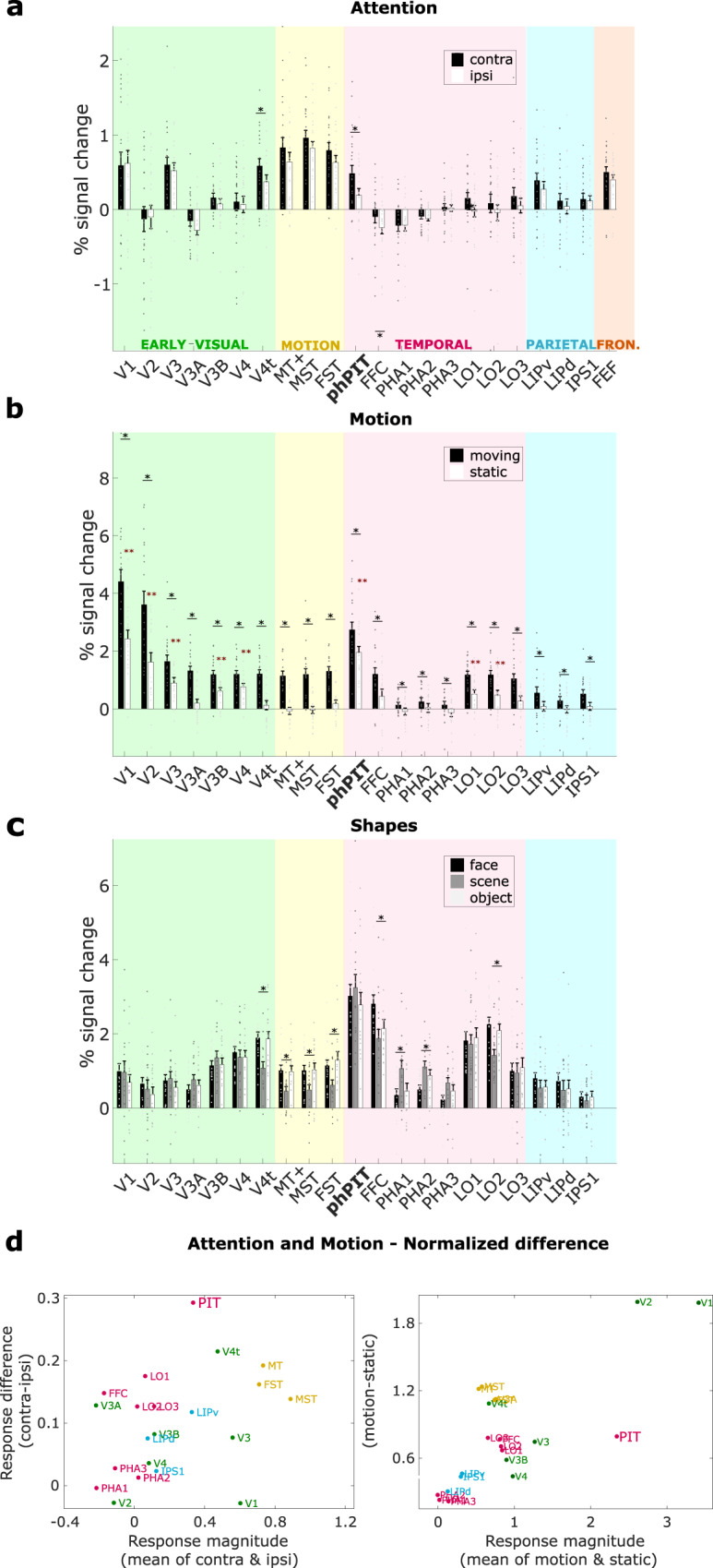
Comparative functional profile of cortical ROIs in the occipital, temporal, parietal, and frontal lobes. **a** Attentional modulation; the bar plot shows the average percentage signal change across subjects for each ROI, expressed as mean across 24 hemispheres; gray points represent the values for left and right hemispheres of each individual subject. Signal extracted during the attend contralateral (Attend contra) and the attend ipsilateral (Attend ipsi) condition are shown in black and gray, respectively. Black asterisks indicate a significantly stronger response differences for attended than for unattended condition (*p* < 0.05, one-sided paired *t-*test uncorrected for multiple comparisons; exact *p*-values are reported in Supplementary Table 2); **b** ROI responses to motion and static stimuli; bar plots show the average percentage signal change across subjects for each ROI in response to moving and static stimuli, expressed as mean across 20 hemispheres; gray points represent the values for each individual subject; red asterisks indicate a significant response for static stimuli *p* < 0.05, one-sided *t-*test uncorrected for multiple comparisons; exact *p*-values are reported in Supplementary Table 2; black asterisks indicate a significantly stronger response differences for moving than for static stimuli *p* < 0.05, one-sided paired *t-*test uncorrected for multiple comparisons; exact *p*-values are reported in Supplementary Table 2. **c** ROI responses to three shape categories; bar plots show the average percentage signal change across subjects for each ROI in response to faces-scenes-objects, expressed as mean across 18 hemispheres; gray points represent the values for each individual subject and hemispheres; black asterisks indicate significantly different responses for the three different stimulus categories (*p* < 0.05, one-way ANOVA uncorrected for multiple comparisons; exact *p*-values are reported in Supplementary Table 2). **d** Attention and motion modulation profiles of ROIs relative to mean activation. Scatter plots show activation differences (vertical axis) as a function of average activation (horizontal axis) during the attention task (left) and the motion localizer (right). The ratio of response difference and response magnitude defines the attention index. V1-2-3-3A-3B-4 visual areas 1-2-3-3A-3B-4, V4t visual area 4 transition, MT+ middle temporal area, MST medial superior temporal area, FST fundus of the superior temporal sulcus, phPIT putative human Posterior Infero-Temporal area, FFC fusiform Face Area, PH1-2-3 para-hippocampal area 1-2-3, LO1-2-3 lateral occipital areas 1-2-3, LIPv ventral latera intraparietal area, LIPd dorsal latera intraparietal area, IPS1 intra parietal sulcus 1, FEF frontal eye field. Source data are provided as a Source Data file.

To further test the profile of response of specific regions to the attention task and the motion, face and scene localizers, and thus provide a richer profile of the selectivity of each region, we implemented a region of interest (ROI)-based analyses (see Methods). Figure 5 shows that area phPIT is characterized by a strong significant attention effect. PIT, in fact, is the area with the largest attention index among areas with a robust positive response to visual stimuli (Fig. 5d, left panel) and the largest response difference of all areas for attended versus non-attended stimulus (Fig. 5d, left panel). Attention effect in PIT are thus stronger than in early visual areas or in motion areas (Fig. 5a).

Motion selectivity exhibits almost the reverse pattern of effects. As the motion localizer confirmed, early visual areas, traditional motion areas MT, MST, FST, and even LO1, 2, and 3 exhibit strong preferences for motion over static stimuli (Fig. 5b). Critically, traditional motion areas are characterized by a strong response to moving stimuli and a non-significant response to static stimuli. PIT exhibits a modulation by motion (Fig. 5b), which is smaller than that of the other areas relative to its visual activation (Fig. 5d), yet a strong activation by static stimuli (red asterisks). Finally, the shape localizer, confirmed specialized processing for faces in FFC, for places in PHA1-3, for objects in LO2. PIT, among the most strongly activated areas, showed instead similar responses for faces, scenes, and objects (Fig. 5c); the response differences between these categories were not significant.

Overall, PIT stood out as a highly and generally visually responsive area. PIT responds strongly to both shape-less motion stimuli and motion-less shape stimuli, suggesting that neurons in this area are not strongly tuned and thus capable of representing any stimulus. PIT also stood out as an area whose activity is very strongly attention modulated, both in absolute terms and relative to its degree of visual activation (Fig. 5d). Importantly, PIT’s response profile across all three tasks strongly differs from that of nearby motion areas (in yellow) as well as from that of nearby temporal areas (in pink).

A direct comparison with attentional and visual characterization of macaque PITd shows that activation patterns in human and macaque areas were quite similar (Fig. 4c). Importantly, in both species, PIT’s functional profile differed profoundly from that of nearby areas and was more similar to dorsal attention areas. In particular, the fact that PIT was not activated by the relevant dimension of the attention task, motion, suggests PIT is generally involved in attentional processing and not specifically in attentive motion processing. This functional mapping clearly shows that in both species attention-modulated PIT is located inside the ventral visual pathway^36^, a further point differentiating PIT from human TPJ.

### Evidence for white matter connections between phPIT and the fronto-parietal attention network

To unravel whether and how phPIT is structurally connected to dorsal attention areas in parietal and frontal cortex, we combined task-based fMRI data with an independent dMRI dataset from a publicly available large-scale project with a cloud-computing platform and reproducible Methods^37^. More specifically, we used the structural–functional cortical parcellation operated by the Glasser atlas^31^ and combined it with diffusion imaging data from over 200 human subjects from the Human Connectome Project^38–40^. For each subject, we segmented cortical volume masks for LIP, FEF, and PIT and then tested their pairwise structural connectivity. We first performed ROI-to-ROI ensemble probabilistic tractography to create a macrostructural model of attention pathways^41^ and then validated the results by applying Linear Fascicle Evaluation, LiFE:^42,43^. Second, we estimated structural properties of the whole tracts (macroscopic level) and within each voxel (microscopic level).

**Fig. 6:**
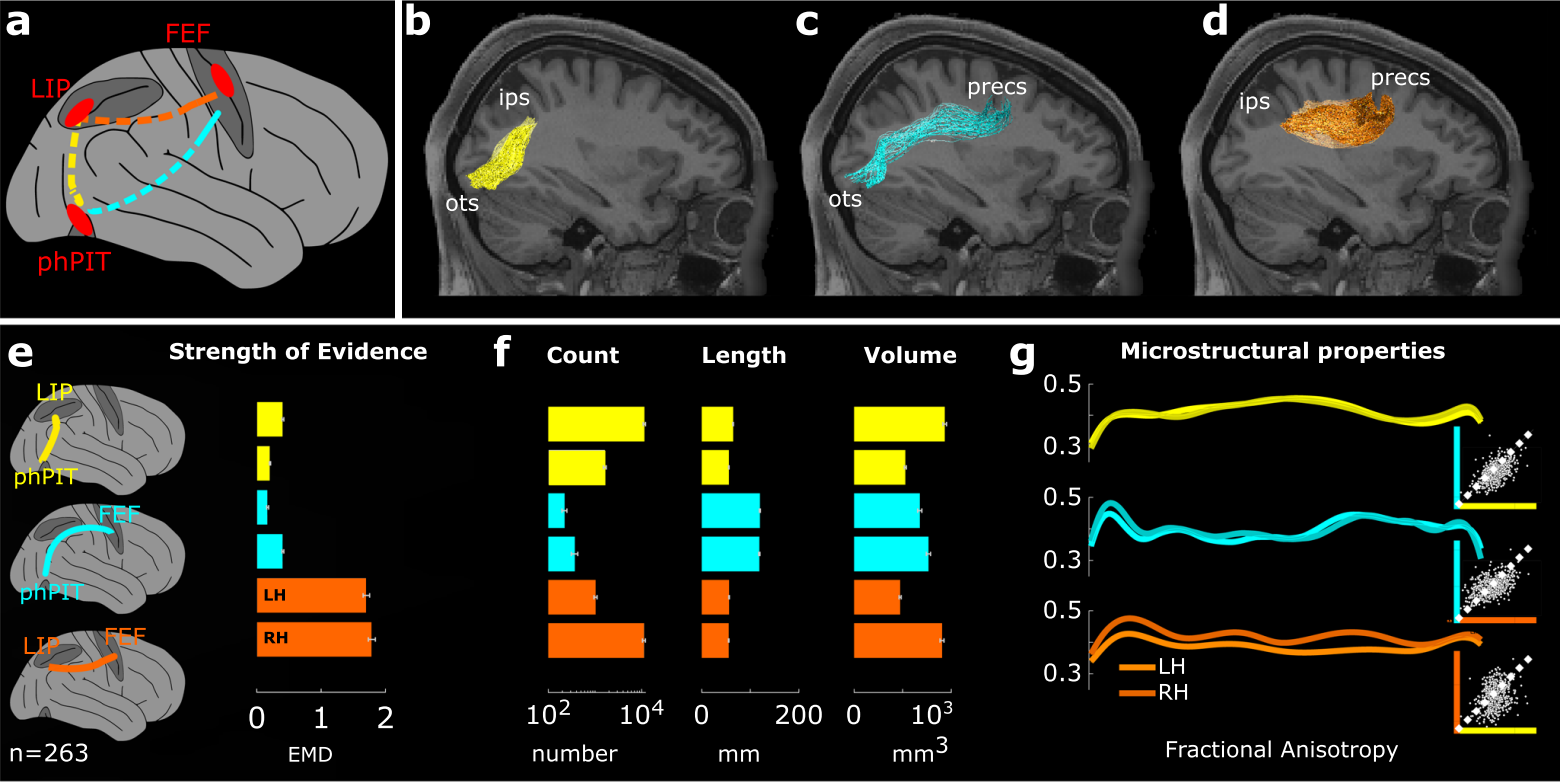
Human dorso-ventral attentional connection identified using tractography. **a** Schematic connections; phPIT–LIP: yellow trace; phPIT–FEF: cyan trace; LIP–FEF: orange trace. **b**–**d** Sagittal-view of phPIT-to-LIP, phPIT-to-FEF, and LIP-to-FEF connections overlaid on T1 image for subject 101006. Conventions as in Fig. 1. **e** The bar plot shows the mean earth mover’s distance (EMD; see also Methods) in support of the existence of the tracts; lower bars for each tract represent the average across the right hemisphere, upper bars across the left. **f** Bar plots show the average streamline number, tract length (mm), tract volume (mm^3^) for functional tracts of 263 subjects; lower bars for each tract represent the average across the right hemisphere, upper bars across the left. **g** Microstructural properties of functional tracts as measured by fractional anisotropy (FA; see Methods). Each line represents the FA value averaged across subjects and calculated along the tract. Insets show direct comparison of individual subject FA between phPIT–LIP and phPIT–FEF connections (top), LIP–FEF and phPIT–FEF connections (middle), phPIT–LIP and LIP–FEF connections (bottom). Data are expressed as mean across 263 subjects, and separately for the two hemispheres; gray points represent the values for each individual subject. ips intra-parietal sulcus, ots occipito-temporal sulcus, sts superior temporal sulcus, preces pre-central sulcus, FEF frontal eye field, LIP lateral intra parietal area, phPIT putative human posterior infero-temporal area, LH left hemisphere, RH right hemisphere. Source data are provided as a Source Data file.

ROI-to-ROI ensemble probabilistic tractography showed direct structural connectivity between phPIT and LIP (Fig. 5b), and between phPIT and FEF (Fig. 5c), as well as the well-known connection between LIP and FEF (Fig. 5d). We observed the core of the tracts at consistent positions for the majority of subjects and hemispheres, with some variability only for the temporo-frontal connection (Fig. S5). Because tractography Methods are prone to false positive results^44^, we next assessed the statistical strength of the evidence supporting the existence of these specific tracts by using the LiFE algorithm combined with a virtual lesion method^42,43^. Briefly, for each subject, we first generated a whole-brain connectome (i.e., a comprehensive collection of streamlines, or putative tracts, representing the brain’s white matter connectivity^45^), and compared it with the subset of all streamlines except the tract of interest (lesioned connectome). The LiFE algorithm refined the connectomes, which, prior to optimization, potentially contained streamlines without significant supporting evidence in the diffusion data. The virtual lesion method calculated the difference between the tractography models with and without a connection of interest, by measuring the models’ prediction of the dMRI data. The Earth Mover’s Distance (EMD^46^; see Methods) was used to measure the strength of evidence for each tract in each subject, because it has been shown to be robust to connection size, volume, and length^42^. For the three tracts of interest here (LIP–FEF, phPIT–FEF, and phPIT–LIP), the mean EMD was significantly higher than zero (Wilcoxon signed rank test, *p* < 0.01; Fig. 6e). This indicates that the tracts non-trivially contributed to the accuracy of the whole-brain connectomes in modeling the predicted diffusion signal, thus providing evidence for their existence. These results are consistent with the hypothesis of direct interconnectivity between attentional nodes located in the frontal, parietal, and temporal lobes.

To characterize the macroscopic properties of the defined white matter tracts, we calculated the number of streamlines, their average length, and their total volume (see Methods). Across subjects, the tract connecting phPIT and FEF was, on average, longer than the phPIT–LIP connection (Fig. 6f, middle panel, cyan vs. yellow), had a lower streamline count, and similar volume (Fig. 6f, left and right panels). The LIP–FEF tract had a similar length as the LIP–phPIT connection (Fig. 6f, middle panel, orange vs. yellow), but a higher count and volume (Fig. 6g, left and right panels). Microstructural properties were measured by fractional anisotropy (FA) and showed higher values for the temporo-parietal connection between phPIT and LIP (Fig. 6g), suggesting differences in degree of myelination, fiber diameter, density, or coherence for this vertical connection^47–49^. Interestingly, phPIT’s connections with either frontal or parietal lobe did not show hemispheric asymmetry in microstructural properties, while the LIP–FEF connection did, consistent with previous literature^50^.

In sum, the morphological evidence from tractography (Fig. 6b–d), the statistical evidence computed through LiFE (Fig. 6e), and the quantitative assessments of corresponding metrics (Fig. 6f, g) all agree in supporting the existence of direct phPIT–LIP and phPIT–FEF connections.

### The vertical pathways connecting phPIT and dorsal attention areas

We next characterized the anatomical and positional characteristics of the human attention network described here by quantifying the spatial relationships between our functionally defined bundles (phPIT–LIP, phPIT–FEF, and LIP–FEF) and previously characterized and established major human white matter tracts^37,51^.

First, we determined whether the phPIT–LIP tract runs through any of the vertically oriented tracts occupying the posterior white matter of the human brain. We segmented the vertical occipital fasciculus (VOF), the posterior arcuate (pArc), and the temporo-parietal connection to the superior parietal lobule (TP–SPL) as in ref. ^51^, and overlaid them with the functionally defined phPIT–LIP connection (Fig. 6a). The phPIT–LIP tract runs predominantly vertically, but also exhibits traversal along the posterior–anterior axis of the brain (Fig. 6a, yellow streamlines). Furthermore, this tract is situated along the medio-lateral axis similarly to the VOF, but differs in its anterior–posterior positioning as it connects to the parietal lobe, whereas the VOF is confined to occipital areas (Fig. 6a, yellow vs. pink streamlines; see also Fig. S7). Nearby, both the pArc and the TP–SPL are noted to exhibit a predominantly vertical orientation, but again have an anterior–posterior elongation different from phPIT–LIP tract and their ventral endpoints are located anteriorly to phPIT (Fig. 6a; yellow vs. red and green streamlines; see also Fig. S6). To estimate and compare the similarity between attentional tracts and the previously characterized major white matter connections, we calculated the percentage of tract overlap (Methods). We found that phPIT–LIP did not reach a degree of overlap higher than 40% with any of the tested anatomical tracts individually (Fig. 6d) and that they overall failed to capture the core of the vertical attentional connection between phPIT and LIP (Fig. S7). This result suggests that the connectivity between dorsal and ventral endogenous attention nodes is not explained by current anatomical segmentation of the white matter in the posterior part of the brain, and thus given the percentage of overlap likely constitute an unreported sub-portion of the pArc.

**Fig. 7:**
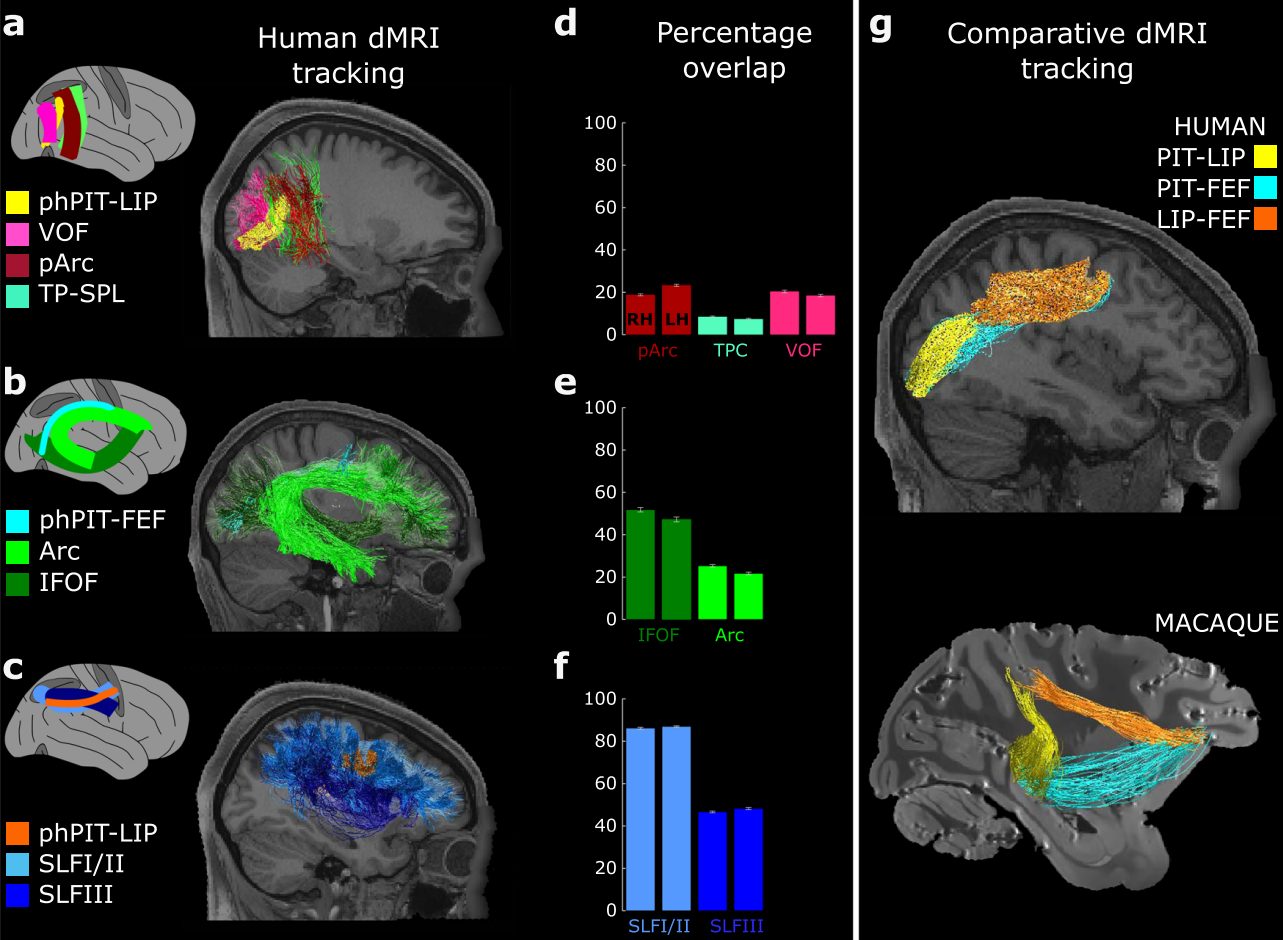
A sub-portion of pArc, Arc, and SLF support the endogenous attention network. **a** Sagittal view of the VOF (pink), pArc (dark red), TP–SPL (green), and phPIT-to-LIP (yellow) connections of subject 101106. **b** Sagittal view of the Arc (light green), IFOF (dark green), and phPIT–FEF (cyan) connectivity of subject 101106. **c** Sagittal view of SLF (dark and light blue) and LIP–FEF (orange) connectivity of subject 101106. **d**–**f** Quantitative overlap, i.e., proportion of functionally defined attentional tracts overlapping with hypothesized anatomical pathways. Data are expressed as mean across 263 subjects; left bars for each tract represent the average across the left hemisphere, right bars across the right. **g** Sagittal view of the human and macaque showing the comparative anatomy of the dorso-ventral endogenous attention network as defined in ref. ^19^. FEF frontal eye field, LIP lateral intra parietal area, phPIT putative human posterior infero-temporal area, Arc Arcuate Fasciculus, IFOF Inferior Frontal Occipital Fasciculus, pArc posterior Arcuate Fasciculus, SLF Superior Longitudinal Fasciculus TP–SPL Temporo-Parietal connection to the Superior Temporal Lobule, VOF Vertical Occipital Fasciculus. Source data are provided as a Source Data file.

We next characterized the white matter pathways that connect phPIT to FEF. This connection might occupy similar volumes of white matter as the inferior frontal occipital fasciculus (IFOF), which connects TPJ to other frontal nodes of the human exogenous attention network through the extreme capsule^52^. Such a finding would suggest that phPIT and TPJ share similar pattern of frontal lobe connectivity, despite their distinct functional roles in attention. Alternatively, the functional difference between phPIT and TPJ may be paralleled by a difference in structural connectivity. The arcuate fasciculus (Arc) may be a good candidate to transmit information related to endogenous attention between frontal and temporal lobes. We found that the phPIT–FEF connection, similarly to the Arc, but differently from the IFOF, travels vertically from phPIT and then enters the dorsal part of the brain through the dorsal component of the Arc (Fig. 7b, cyan vs. green streamlines). Despite the similar traversal pattern, the quantitative overlap of phPIT–FEF with the Arc was low (~20%) because the Arc does not run dorsally enough and does not extend posteriorly enough to reach phPIT. The overlap of phPIT–FEF with the IFOF was more than 40% because of the similar patterns of their posterior endpoint terminations, though the cores of these tracts remain inarguably distinct (Fig. 7b cyan vs. dark green). Once again, the apparent inconsistency between these tracts’ pathways and their degree of overlap suggests current white matter taxonomy does not seem to account for connectivity to the phPIT.

Finally, we investigated whether the LIP–FEF connection is a subcomponent of SLF II or SLF III, as suggested by previous diffusion studies^50^. We found that the FEF–LIP tract is part of the most dorso-medial region of SLFII, while SLFIII runs much more ventrally (Fig. 7c, orange vs. light and dark blue streamlines). Quantitative analyses confirmed that more than 80% of phPIT–FEF overlaps with SLFII, while with the SLFIII it is less than half (Fig. 7f). Thus fronto-parietal connection patterns are well explained by current segmentations of anatomical tracts.

A direct comparison with diffusion data from non-human primates^19^ shows that dorsal and ventral attention nodes are directly connected in both species. We found that, similar to the macaque, a vertical temporo-parietal tract anterior to VOF connects phPIT with LIP in humans (Fig. 7g, yellow streamlines), that phPIT and FEF traverses similar white matter volumes as the Arc in humans and passes through the EmC in macaques (cyan streamlines) which stands as a difference between the two species, and that SLFII connects LIP and FEF in both species (orange streamlines; see also Fig. S7).

Overall, we establish that a sub-portion of the pArc likely connects phPIT with parietal attention area LIP and that the most posterior and dorsal part of the Arc fasciculus connects phPIT with frontal attention area FEF. The existence of these vertical pathways connecting attention nodes in both humans and macaques, suggests the extended dorso-ventral attention network as an evolutionarily preserved organizing principle of primate cognition.

## Discussion

The control of endogenous attention has traditionally been attributed, with much support, to a fronto-parietal network^8,9,14,15^. Our results suggest that there is an additional area of attention control, located in a specific part of the temporal lobe, phPIT, and that this area constitutes a node of the endogenous attention control network. While further investigations will need to strengthen this proposal, our finding has important consequences for our understanding of attentional control networks, and the neural architecture of cognition at large.

While it is hard to demonstrate a role in attentional control solely based on unimodal neuroimaging data, within the current multimodal and multispecies framework, area phPIT revealed to share critical properties for attentional control with dorsal attention control areas, with its putative homolog in the macaque monkey, and with theoretical proposals for attention control areas (Supplementary Table 4). We found phPIT, but not TPJ, to be activated during a highly demanding attentive motion discrimination task. There are different reasons why a particular area is recruited by attention. One is to enhance activity in areas encoding the task-relevant features^53–59^. The attentive motion-discrimination task, indeed, recruited visual areas like the MT+ complex, which processes motion information. But phPIT was attention-modulated, yet not motion selective, a property it shared with the dorsal attention control areas in parietal and frontal cortex. Attentional modulation in the absence of feature tuning is a central concept of salience map and feature integration theories of attention^17,60,61^: for neurons to control the spatial focus of attention, they should not be (strongly) tuned to specific features, allowing them to respond to any stimulus falling into their receptive fields. This property has been found in parietal and frontal attention control areas^17^ and, recently, also in area PITd, both at the level of the entire area and the level of individual neurons^10,16^. Importantly, attention modulated area PITd was localized in the macaque monkey with the exact same paradigm^10^ yielding the same pattern of attention modulation in the absence of motion tuning as we have found here in humans and at the very location predicted from global macaque–human inter-species brain mapping^30–32^. These functional similarities suggest that human phPIT may contain the same single-unit properties of a salience map as found in the macaque monkey. Interestingly, previous studies have discussed the possible role in attention of an area located anterior to, and possibly compatible with, phPIT (referred as fusiform);^62,63^. More specifically, fusiform along with other regions, has been shown to have predictive signals that reflect the use of the cued information, thus supporting the hypothesis that this activity may represent maintenance of attention at the cued location^63^. Despite this circumstantial evidence, phPIT role in attentional control has never been stated explicitly and has remained largely uncharacterized. In this context, we believe our work represents a key step forward in that it connects and definitively validates earlier observations. Critically, while previous fMRI studies showed less sustained attentional signals in fusiform^62^, recent fMRI-guided electrophysiological recordings point to strong and sustained responses in macaque PIT (>4 s)^16^. Yet another study suggests that the divide between areas with source and target roles in attention might be less distinct than previously thought, when considering the area representational properties^64^. Interestingly, the authors show that several fronto-parietal regions, but also an area compatible with phPIT location, encode continuous representation of sensory information. This suggests that even in this framework, PIT, LIP, and FEF share important similarities.

Despite its localization in the ventral visual stream, phPIT was activated during a sustained endogenous attention task, but not during stimulation with high-level visual stimuli, such as faces. In this regard also, phPIT was thus more similar to dorsal attention areas like LIP and FEF than to its immediately neighboring areas. Again, these properties are shared with the attention-modulated macaque area PITd, similarly positioned right next to two face areas^10^, with only weak tuning to shape and not to color^16^, again matching theoretical predictions for an attentional control area. Yet PITd and phPIT are ideally positioned within the ventral stream to gather information from a great variety of features^65–70^, including words^71,72^, faces^10,73–75^, and gaze^76,77^, which attract and direct attention^78,79^. phPIT might be thus playing an important role in multiple attention tasks and cognitive computations that require access to this visual information at the interplay between multiple systems, a concept strongly supported by the recently described structural link between the ventral occipito-temporal cortex processing visual words and the attention network^72^. The capability of capitalizing on different types of information is at the heart of attentional function, as clearly expressed, e.g., in feature integration theory^60^, and might explain why an attention control area might be positioned deep inside the temporal lobe.

This location, however, poses challenges. For phPIT to function as a source of attentional control, it would need to negotiate the focus of attention with other areas of attentional control located far away in the parietal and frontal lobes. These functions would be greatly aided by direct anatomical connections. As in the macaque monkey, our work provides evidence for direct connections between phPIT and parietal and frontal attention control areas in humans. These results provide an independent evidence for homology between PITd and phPIT – the areas share a similar functional profile, the same anatomical location and embedding within feature selective cortex, as well as connectivity. Thus the core circuitry of attentional control appears to be evolutionarily preserved.

The strongest evidence for a role of area PITd in attentional control comes from artificial activation and inactivation of neurons in this region in the macaque monkey^16^. Electrical stimulation of area PITd during performance of the attentive motion-discrimination task described in this study has been shown to cause specific changes of behavior as if attention was both drawn to and enhanced for the stimulus located in the portion of the visual field for which the artificially activated neuron was responsible^16^. Both selective performance improvements and impairments were effectively induced by PITd stimulation, reflecting precisely the behavioral changes that would ensue a shift in the focus of attention. Importantly, motion processing was not impaired by any of these highly artificial modulations of activity^16^. Conversely, selective inactivation of the region caused behavioral changes akin to hemispatial neglect^18^. Although similarly direct evidence is lacking in humans, neuropsychological evidence has increasingly pointed to a role of the temporal lobe in attentional control^80,81^. Lesions to a wide range of cortical locations, including posterior parietal cortex^23,82^, angular gyrus^5,83^, supramarginal gyrus^84,85^, dorsolateral and inferior fontal cortices^7,86,87^, as well as TPJ^3,4^ have all been associated with spatial neglect. The more recent shift of research attention to the temporal lobe is beginning to reveal that ventral temporal regions, possibly including phPIT, are implicated in a specific component of neglect referred to as allocentric/object-centered neglect^80,81^. In these studies, patients displayed impaired processing of the inherent left and right side of visual objects independently of their absolute position with respect to the observer^80,81^. While future research will need to determine the exact role of phPIT and neighboring regions in these deficits, these results point to a specific role phPIT might play in the control of object-based attention and in the representation of space in object-centered/allocentric coordinates.

One implication of our work is that, not only damage to gray matter, but also damage to specific white matter regions, should cause neglect-like deficits in attentional control. White matter tracts in humans have indeed been proposed before as important factors determining the severity of neglect^84,88^. It has been shown that anatomical parietal–frontal disconnections play a relevant pathophysiological role in promoting chronic neglect^89,90^. The discovery of an attention node in the temporal lobe posits that a set of connections with vertical orientation may be involved in endogenous attention and neglect. Specifically, our findings imply that damage to the connections between phPIT and dorsal attention areas, namely to the phPIT–LIP bundle and to the phPIT–FEF tract, might specifically impact the ability of the brain to combine information about space and features of the outside world. Here we suggest that a sub-portion of the pArc and the Arc may be critical targets for future clinical investigations, in addition to the better characterized SLF. We also show that a direct correspondence between the attentionally defined vertical tracts and standard anatomical segmentations is currently lacking. The phPIT–LIP tract is traveling partially via a sub-portions of pArc and partially via a sub-portions of TP–SPL. Likewise the phPIT–FEF tract followed the Arc pathway, but resulted in a lower degree of overlap with it, and is potentially compatible with other vertical bundles like the superior fronto-occipital fasciculus^91^. A fine-scale functional characterization of the tracts supporting the extended endogenous attention network is therefore critical to a full characterization of attentional control circuits, their vulnerabilities, and their developmental potential.

The finding of specific dorso-ventral tracts connecting the nodes of the network controlling endogenous attention, has further implications for our understanding of the organization of the visual system. The main organizing principle is the proposed separation into two (or more^92^) major information-processing streams traversing the posterior–anterior axis of the brain^93^. However, the separation of information into parallel streams implied by this framework appears to be at odds with the requirement of visual attention to integrate spatial and featural information^60,94,95^. One possible solution to this binding problem, is the development of shape-selectivity in the dorsal stream^96–98^. Another solution is the existence of vertical connections between the information streams. The fact that we above provide evidence supporting the ascription of specific attentional functions to two vertical tracts, supports the latter idea, providing a putative structural substrate for attentional integration that capitalizes on the full functional repertoire of all streams. The three-node network described here and its vertical connectivity may thus be a general theme, a second organizing principle of the visual system in humans^42,99^ and macaques^19,100^. This principle likely extends from one cognitive function, attention, to others like skilled grasping^101^, language^102^, as well as to high-level mental representations exerted by the default mode network^103^. Taken together, an organizational motif that is shared across networks and species raise the possibility that a macroscale organization might emerge from evolutionary and developmental constraints^103^.

**Fig. 8:**
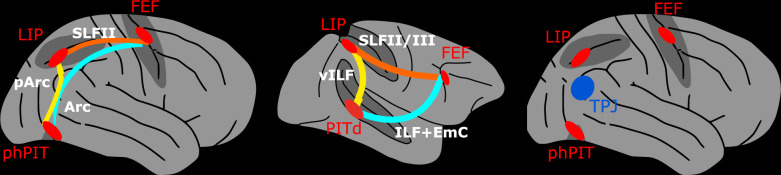
Human extended endogenous attention network. Whole brain model and structural connectivity of the attention network as defined by functional activation (red areas) and structural connectivity in humans (leftmost panel) and macaques for comparison (middle panel). Colored solid lines represent connections and pathways between attention nodes phPIT, LIP, and FEF. The rightmost panel shows the spatial relationship between the extended endogenous attention network (red areas) and the TPJ, the most posterior node of the exogenous attention network (blue area)^9^. FEF Frontal Eye Field, LIP Lateral Intraparietal area, phPIT putative human Posterior Infero-Temporal area, Arc Arcuate Fasciculus, EmC Extreme Capsule, ILF Inferior Longitudinal Fasciculus, pArc posterior Arcuate Fasciculus, SLF Superior Longitudinal Fasciculus, vILF vertical branch of the Inferior Longitudinal Fasciculus.

Our results suggest that the “dorsal” fronto-parietal network includes a ventral node to exert control of endogenous attention (Fig. 8). The proposed model is compatible with the existence of two attentional control networks dedicated to the endogenous and exogenous control of attention, respectively, the latter including TPJ (Fig. 8, rightmost panel);^9^. TPJ has been long considered a core node of the exogenous attention network. However, recent evidence suggests a less distinct separation between the endogenous and exogenous attention network and their dorso-ventral segregation. Besides exogenous attention, TPJ is also involved in endogenous attention through de-activation during cued orienting^20,26^ and in late phases target-processing linked to contextual updating^20–22^. Moreover, the dorsal-ventral dichotomy of attentional control might not fit with the role that the superior parietal lobule might play in exogenous re-orienting to invalidly cued targets^104–106^. Our findings complement these views and do not support earlier speculations about the possible homology between macaque PITd and human TPJ^18^, the only attention control area known in the human temporal lobe to date. In fact, our data indicate that the area corresponding most closely to the macaque attention-control area PITd, is not TPJ, but phPIT, in virtue of both functional and anatomical characteristics. First, the motion discrimination task that originally localized the attentional component of macaque PITd failed to elicit activation in human TPJ. Second, PITd and phPIT share the same anatomical location deep within the temporal lobe, as well as the same location relative to nearby retinotopic^32^ and feature-specialized areas. On the contrary, TPJ is located more dorsally at the intersection between the temporal and parietal lobes and is surrounded by – and perhaps partially overlapping with – high level, multimodal regions involved in complex social cognition^107,108^. Finally, PITd and phPIT showed a similar pattern of direct structural connectivity with fronto-parietal endogenous attention area, while TPJ is thought to form, with exogenous ventral frontal areas, a neuroanatomical substrate segregated from the endogenous attention network, as reflected in the correlation structure of spontaneous activity^29^. Overall, our results provide evidence for a homology between macaque and human PIT and show important functional and anatomical differences between macaque PIT and human TPJ.

In conclusion, our work advances the understanding of the architecture of attentional control by suggesting a three-node cortical network, with each node located in a different cortical lobe, indicating the structural substrate to be associated with this cognitive function. The findings reported here have important implications for the understanding of the mechanisms and functional capabilities of attention, of attentional dysfunctions, and of the evolution of attention systems; they also challenge current cognitive theories of endogenous attention and of functional organization of the primate brain.

## Methods

### fMRI

#### Subjects

Twelve volunteers participated in the fMRI experiment (5 females, 7 males; mean age, 23 years ± 2 years). Sample size and experimental approach was chosen after Kolster and colleagues^32^ who localized phPIT successfully and consistently across subjects (11 participants) by using several tasks and localizers. In the current study, individual subjects were scanned over three days, two of which fully dedicated to the attention task and one to the localizers (see details below). This was critical, because of the small size of phPIT and because of the variability of its location across different subjects. In addition, because of the comparative nature of the current study, before the fMRI sessions each subject was trained on 5 separate days and performed 6 sessions of 120 trials each day. This was done to minimize effects of learning in the data and better compare the current dataset with that in Stemmann and Freiwald^10^, where non-human primates had been also extensively trained before the fMRI sessions. All participants had normal or corrected-to-normal visual acuity, and no history of mental illness or neurological diseases. Prior to the experiment all volunteers were tested on the Freiburg Visual Acuity Test, the Titmus-Test for stereopsis, and the Ishihara-Test for color-vision. Subjects were compensated for their participation in the experiment. The study was approved by the ethics committee of the University of Bremen, and all volunteers gave written consent in accordance with the Helsinki declaration before the experiment.

#### Attention task

We used a motion discrimination task for the study of sustained endogenous spatial attention. Stimuli were presented on a CRT monitor positioned 80 cm in front of the subject’s eyes with a resolution of 1024 × 768 and a refresh-rate of 100 Hz. The eye position was monitored at 60 Hz during all fMRI scanning sessions using a custom-made eye tracker system positioned at the back of the to track pupil position and corneal reflection. Stimuli were generated and presented by a custom made software (Visiko). During scanning, each trial began with a fixation spot (0.2° of visual angle) surrounded by eight possible peripheral saccade targets (0.2° of visual angle) positioned 7.5° from the fixation spot. After 500 ms, a small horizontal bar (length 0.2°, width 0.6°) left or right to the fixation spot cued the behaviorally relevant position (left or right) and disappeared after 500 ms when the random dot stimuli appeared on the screen. Random dot stimuli were presented in circular aperture with 4.5° diameter at a horizontal distance of 4.5° from the center on the horizontal meridian. Dot density was 5 dots/° and motion velocity 6°/s. Motion direction changed randomly every 60 ms, along vectors oriented at random multiples of 20°, until the prolonged motion event of 500 ms occurred at a random time point after at least 30 and at most 60 brief motion events (Fig. 2a). Trials were rated successful if the subject reported the motion direction of the prolonged motion event by a saccade to one of eight peripheral saccade targets. Subjects were required to maintain fixation until the prolonged event (i.e., a translation in direction of motion lasting 500 ms, instead of 60 ms) was detected and discriminated. At this point, subjects were expected to saccade towards the peripheral saccade target. Stimulus presentation, eye movements, and behavioral monitoring were integrated online by custom written software. Therefore, whenever the subject broke fixation before the expected time the trial was aborted and considered as an early selection. The analysis of behavioral performance showed that, on average, subjects broke fixation only in 3.1% of the trials which were considered early selections (Fig. 1c).

The two attentional conditions were organized in two different blocks (“right” and “left” blocks) and separated by 5 s of passive fixation blocks (Fig. 2b). Subjects had to complete seven attention trials successfully before the block would switch from left to passive fixation to right, or viceversa (average block duration ~30 s). In ‘passive fixation’ blocks the trial structure was exactly the same as attention trials, but no attentional cue was displayed and the subjects were tasked to maintain fixation on the central dot while passively viewing the moving stimuli (Fig. 2b). A second type of passive fixation condition, where only the fixation spot was shown and moving dots were not presented, was interleaved during the attention task (Supplementary Fig. 2C). The two attentional conditions were dissociated from saccade planning, since saccades to any of the eight different targets were generated equally frequently in the two blocks. In each attention trial, of the 8 possible motion directions, one was chosen for the target, and a different one was chosen for the distractor. This resulted in five main behavioral outcomes: the subject could saccade into the motion direction displayed by the target (“hit”) or the direction of the distracter (“selection error”) or to 1 of 6 remaining targets (“discrimination error”); the subject could fail to respond to the prolonged event (“missed detection”) or respond before the prolonged event actually occurred (“early selection”). After removing fixation breaks, performance was calculated as the percentage of each response type over the total of responses. Per each participant, we scanned 4 complete runs, each containing the two aforementioned stimulus conditions.

#### Localizers

All subjects were also scanned in two additional localizer experiments. Per each participant, we scanned 1 complete run per localizer, each containing the stimulus conditions specified below. To localize motion-responsive areas, we used a set of full-field optic flow stimuli, changing between inward and outward motion approximately every second, and a full-field static presentation of random dots. Stimuli subtended the entire projection screen. Blocks of 18 s with either optic flow oscillating inward and outward with a frequency of 0.9 Hz or a full-field presentation of static random dots oscillating with a blank screen with a frequency of 0.9 Hz. Moving stimuli were contrasted with static stimuli. It should be noted that the motion conditions we used in this standard localizer^109^ were inward–outward optic flow patterns. These stimuli have the advantage of allowing subjects to fixate most easily. However, they are also visually very powerful and, we suggest, might attract a subject’s attention more than static controls. This might explain a general offset of activation for the motion over the static condition across all ROIs in occipital, temporal parietal, and frontal areas.

To map shape selectivity in phPIT and nearby areas, we used colored pictures of non-face objects, faces, landscapes, and scrambled version of these. The stimuli were presented in four blocks of 18 s in which the image changed within category every 500 ms. Between blocks a 9 s blank screen was presented. Each block was presented three times.

Finally, to more accurately attribute boundaries of functional areas we performed meridian mapping and a center–periphery mapping experiment. The retinotopic mapping procedure to define early visual areas was composed of two alternating 18 s blocks experiment (V and H), separated by 9 s fixation periods with a gray background only. During block type V, a vertical black-and-white checkerboard wedge (20° width) was shown with 4 Hz contrast reversals; during block type H, a horizontal wedge was shown. The data were analyzed by contrasting blocks of vertical checkerboard wedges with blocks of horizontal checkerboards.

For the center periphery mapping, to effectively separate activations resulting from foveal stimuli (fixation spot and bar cue) from activations resulting from the random dot stimuli, we modified the meridian mapping stimulus to alternate not between blocks of horizontal and vertical wedges, but between a foveal, an intermediate, and a peripheral ring displaying the checkerboard pattern, contrast inverting at 4 Hz frequency. The central ring was ranging from 0.8 to 2.3° (to encompass the spatial extent of the bar cue in the attention task); the intermediate ring was ranging from 2.7 to 7.2° (to encompass the spatial extent of the RDS in the attention task) and the peripheral ring was ranging from 7.5 to 12.5°. Each eccentricity was presented for 18 s interleaved with 9 s fixation five times. Per each participant, we scanned 1 complete run of the meridian mapping and 1 of the eccentricity mapping.

#### Scanning procedures

All scanning was performed in a 3 T MR scanner. For the attention task, each subject was scanned on two separate days for up to three runs of 420 volumes (~18 min) each. Functional time series consisted of single-shot echo-planar images (EPI): repetition time (TR) 2.51 s, echo time (TE) 30 ms, field of view 192 × 192 mm and 3 × 3 × 2.7 mm^3^ voxel size (38 slices). In addition, a high-resolution anatomical volume for each subject was obtained (3D-MPRAGE, 256 × 256 matrix, 1 × 1 × 1 mm^3^ voxel size).

#### Data analysis

Imaging data were analyzed using a block design in SPM8 (statistical parametric mapping, Wellcome Department of Imaging Neuroscience; London, UK), under Matlab R2010a. Functional scans were slice-time- and motion- corrected, spatially smoothed with a Gaussian kernel of 7 mm full-width-at-half-max (FWHM) and finally co-registered and warped to SPM8 standard space. Statistical analysis followed the procedures for the monkey functional data in ref. ^10^. Activation during active attention task was contrasted with fixation task (Fig. 2b) and shown at a significance level of *p* < 0.001 FWE (family-wise error). Motion selective, shape-selective, face-selective, and scene-selective areas were localized based on functional contrasts (*p* < 0.00001) and standard Talairach coordinates. Area phPIT was defined based on Talairach coordinates relative to those of MT+^32^ and its location in the posterior end of the inferior occipital sulcus, ventral to the lateral occipital sulcus (Supplementary Table 1). For consistency to the approach used in the macaques, the group analysis was performed as a fixed effects analysis concatenating all scanning sessions of all subjects together. In addition, the group analysis was also performed as a random effect analysis. The results were consistent with the two approaches (see Figs. 2 and 3, Supplementary Fig. 2). Both types of group analyses were conducted in SPM8 standard space after volumetric smoothing. Single subject analyses were displayed in each subject’s native surface space. ROI analyses were performed using a random effect approach using SPM8 and marsbar toolbox version 0.44^110^. We defined ROI using the Glasser atlas combined with a functional characterization of eccentricity and retinotopy. The attention-modulated portion in posterior IT cortex (approximately ventral to motion areas and in between face areas) corresponded for most subjects to areas phPIT and V8 of the Glasser atlas, with some inter-individual differences found (see also ref. ^32^) not fully reflected by the Glasser atlas, as a standard of the average brain. For this analysis, we thus used the retinotopic/eccentricity representation of the visual stimuli of the main experiment (see Supplementary Fig. 4) for subsequent ROI analysis, referring to this region with the more inclusive term PIT. The division of this region into just two areas, PhPIT and V8 is not a generally agreed-upon standard studies (e.g., refs. ^32,35^). Thus in future experiments, attentional modulation will need to be profiled, in individual brains, against detailed retinotopy and functional characterizations including the ones of the current study, but also color stimuli that define V8. For each Glasser area of interest we extracted the centroid and defined a 4 mm sphere around the centroid. We quantified activity in each ROI to determine the responsivity profile of the region to the attention task, the motion, and the face-scene-object localizers. For the attention task and the motion localizer, we ran a one-sided paired *t*-test for each ROI to assess the significance of the differences between the two tested conditions (attended vs. unattended; moving vs. static stimulation). Additionally, we tested whether any given area was significantly activated by static stimuli (one-sided *t*-test). For the shape localizer, we run a one-way analysis of variance (ANOVAs) to test for significance differences between responses to faces, scenes, and objects. Statistical tests were uncorrected for multiple comparisons and significance was set at *p* < 0.05. Results are described in the Results and reported in Fig. 5 and Supplementary Table 2. We quantified the modulation and selectivity profiles of each ROIs by comparing the response difference (attend contralateral vs. attend ipsilateral; or moving vs. static) with response magnitude (average attend contralateral and ipsilateral; or average moving and static), thus providing a profile of the selectivity of each region corrected for the amount of overall response.

### dMRI

#### Diffusion MRI data

We used magnetic resonance diffusion imaging datasets acquired by the Human Connectome Consortium^38–40^. The HCP data were acquired at multiple *b*-values (*b* = 1000, 2000, and 3000 s/mm^2^) and 90 diffusion directions per gradient strength, with 1.25 mm isotropic resolution. Diffusion MRI data were analyzed using the online platform brainlife.io, links to the code are provided in the text. The following software were used: MRtrix version 0.2.12 (Tournier et al.^111^) for tracking, FreeSurfer tools version 6.0.0, Matlab R2019a, Trackvis version 0.6.1 for visualization. Data were preprocessed by WU-Minn HCP Consortiums using Methods that are described in ref. ^38^. All MRI data were oriented in neurological coordinate reference scheme (Left–Anterior–Superior) and the bvecs files were oriented correspondingly. The brainlife.io applications implementing these operations can be found in Supplementary Table 3 (T1 AC-PC Alignment; Register to T1; dMRI Shell Splitting). No additional denoising, eddy current, or head movement correction was applied beyond that performed by the data providers.

The use of HCP dataset had important advantages in the current study. First, it gave us access to high quality data; second, it allowed us to evaluate the statistical evidence supporting the existence of the fascicles of interest, a procedure that benefits from a big data analysis approach and that is particularly important in a field – tractography – where false positives are a matter of concern (the results of validation are shown in Fig. 5e). Third, it strongly favored the development of a data processing pipeline fully accessible as computable applications via open services hosted at brainlife.io, or as static code (links to public github repositories with the source code are included for each step described below and in Supplementary Table 3). This was done in an effort to make the data and services utilized in this work open and available for replication, extension, and reuse^37^.

#### Functional and anatomical MRI: tissue segmentation

Tissue segmentation was critical to interrelate fMRI with dMRI results. The two datasets were interrelated in three different ways. Attentional and visual regions of interests (ROIs) were defined using the Glasser atlas, an atlas obtained via semi-automated neuroanatomical approach, which delineates 180 areas per hemisphere, demarcated by sharp changes in cortical architecture, function, connectivity, and/or topography^31^. This parcellation was internally validated, by confirming that a number of specialized areas (e.g., motion areas, face-selective areas, early retinotopic visual areas) were accurately defined by the Glasser parcellation (Supplementary Figs. 3 and 4). 263 subjects were used in this study and were selected among those used to generate the Glasser atlas parcellation. The atlas was aligned to the individual subject space (Multi-Atlas Transfer Tool, Supplementary Table 3). Three areas of interest (phPIT, LIPd, and FEF) were extracted from the atlas, transformed into volume space, and then inflated to include voxels at the intersection between gray and white matter (ROI Generation Tool, Supplementary Table 3). This method has been previously used in other studies^19,42,112,113^. To further interrelate our fMRI results with the dMRI dataset and check on the effect of ROI choice on tracking results, we functionally defined two additional sets of ROIs. The first additional set was obtained by thresholding fMRI activation during the both attention task and localizers (*p* < 0.0001 for attention, *p* < 0.00001 for localizers). The second additional set was obtained and by generating spherical ROIs (radius = 10) around the peaks of activation (see Talairach coordinates in Supplementary Table 1). These two sets of ROIs were aligned to the individual subject space (Attention ROI Warp, Supplementary Table 3) and used for tracking for a subsample of subjects (*n* = 50). The results from different tracking approaches were compared and gave consistent results (Fig. S7).

#### Tractography

Tracking of potential streamlines was performed using MRtrix 0.2^111^. A white matter tissue mask generated from Freesurfer’s aseg parcellation was segmented and subsequently used as a seed mask for connectome generation. The white matter tissue mask and two ROIs of interests (e.g., LIP and phPIT or LIP and FEF) were used as seed regions for generating single tracts of interest. We used constrained spherical deconvolution (CSD^114^) and probabilistic ensemble tractography^41^ to reduce the possibility that tractography estimates would fail to accurately reproduce the diverse range of white matter architecture and miss a real fascicle (ROI to ROI Ensemble Tractography and Ensemble Tractography; Supplementary Table 3). Specifically, we used four curvature thresholds (minimum radius of curvature 1, 2, 3, 4) and four values of maximum number of harmonics (*L*_max_ = 2, 4, 6, 8). We set other parameters as follows: step size 0.2 mm; maximum length 200 mm; minimum length 5 mm. For each tract and connectome, we generated 200,000 streamlines. Following generation, each subsequent tract and connectome was merged into a single fiber structure used for further analyses.

For each tract, the set of potential streamlines was refined by removing outliers on the basis of length and distance from the core portion of the tract^115^. Specifically, for tracts defined with cortical ROIs, we removed streamlines with length ≥4 SD longer than the mean streamline length in the tract, and position ≥4 SD away from the mean position of the tract (Remove Tract Outliers, Supplementary Table 3). To get a quantitative description of the tracts of interest we measured the number of streamlines, average tract length, and tract volume (number of voxels touched by a streamline × voxel volume) (Tract Statistics, Supplementary Table 3). We then evaluated tract microstructural properties by calculating fractional anisotropy (FA). We used Tract Profiles-App (Supplementary Table 3), which computes a core representation of each tract and extracts metrics of interest along equidistant “nodes” of the tract, allowing for comparison across individuals’ subject tracts.

To evaluate the statistical evidence supporting the existence of the fascicle, we used a Linear Fascicle Evaluation method (LiFE)^42^. The algorithm estimated how much each fascicle in the candidate connectome contributes to predicting the diffusion signal and assigned a weight to each streamline. We then eliminated fascicles with zero weight to create an optimized connectome and optimized tracts (Linear Fascicle Evaluation, Supplementary Table 3). We subsequently applied a virtual lesion method^42,116^ to characterize the strength of evidence supporting the fascicles of interest. “Lesioned” connectome models were generated by excluding the fascicle from the optimized connectome. The prediction accuracy of lesioned models were iteratively compared with those of the optimized connectome (“unlesioned” model). The Earth Mover’s Distance (EMD^46^; see Methods) was used to measure the strength of evidence, because it has been shown to be robust to connection size, volume, and length^42^. The EMD values were measured independently for each subject. An EMD value significantly above zero indicates that there is dMRI data supporting the connection of interest. Significance was tested with Wilcoxon signed-rank tests.

To compare functional tracts with standard anatomical fascicles we segmented the whole brain connectome by using waypoints ROIs that isolate the central portion of the tract where the fibers are coherently bundled together and before they begin diverging towards cortex^51^. Sixty-one major human white matter tracts were segmented and refined by removing the fiber outliers (White matter Tract Segmentation, Supplementary Table 3). We then focused on the second and third branch of Superior Longitudinal Fascicle (SLFII and SLFIII), the Inferior Fronto-Occipital Fasciculus (IFOF, which passes through the human Extreme Capsule^117^), the Arcuate Fasciculus (Arc), the vertical tracts Ventral Occipital Fasciculus (VOF), the posterior Arcuate fasciculus (pArc), and the Temporo-Parietal connection to Superior Parietal Lobule (TP–SPL). To quantify the similarity between attentional tracts and anatomical pathways, and therefore facilitate testing hypotheses regarding potential tract pathways, we calculated the percentage of tract overlap as overlapping volume in the two tracts (unique voxels occupied by streamline coordinates) divided by the total volume of the attention tract. To visualize anatomical tracts endpoints and compare them with the location of attention ROIs, we generated tract endpoint maps (Generate tract endpoint maps, Supplementary Table 3) where we applied a gaussian smoothing kernel of a 7 mm radius to the tract endpoints as decay function for determining which gray matter voxels are “near” an endpoint. Endpoint maps were combined across all subjects and visualized on MNI152 along with attentional ROIs as in ref. ^51^.

### Reporting summary

Further information on research design is available in the Nature Research Reporting Summary linked to this article.

## Supplementary information

Supplementary Information

Reporting Summary

Peer Review File

## Data Availability

Source data are provided with this paper. The full data sets used for and generated by the connectivity analyses are available at 10.25663/BRAINLIFE.PUB.16 for the main text and at 10.25663/BRAINLIFE.PUB.17 for the control analyses presented in the supplementary information. Other data are available from the authors upon request. Source data are provided with this paper.

## Source data

Source Data

## Code availability

Functional MRI analyses have been performed using freely available spm code. Diffusion MRI analyses have been performed on brainlife.io and the code used is available at 10.25663/BRAINLIFE.PUB.16 for the main text and at 10.25663/BRAINLIFE.PUB.17 for the control analyses presented in the supplementary information. Specific references to the code are provided in Supplementary Table 3, which includes the doi and the link to the github repository where the code can be downloaded. Other code is available from the authors upon request.
