# Supplementary material for: The human endogenous attentional control network includes a ventro-temporal cortical node
Source: Nat Commun. 2021 Jan 15;12:360. doi: 10.1038/s41467-020-20583-5 (PMC7810878; doi:10.1038/s41467-020-20583-5)
Supplement: Supplementary file 1 — Supplementary Information [file 41467_2020_20583_MOESM1_ESM.pdf]

Supplementary Information

The human attentional control network includes a ventro-temporal cortical node

Ilaria Sani[a,b*], Heiko Stemmann[c*], Bradley Caron[d], Daniel Bullock[d], Torsten Stemmler[c], Manfred Fahle[c], Franco Pestilli[d,e], Winrich A. Freiwald[a,f]

a Laboratory of Neural Systems, The Rockefeller University, 1230 York Avenue, New York, NY 10065, USA.

b Laboratory of Neurology & Imaging of Cognition, University of Geneva, Chemin de mines 9, 1202, Geneva, CH.

c Institute for Brain Research and Center for Advanced Imaging, University of Bremen, D-28334 Bremen, Germany.

d Department of Psychological and Brain Sciences, Indiana University, Bloomington, IN, USA.

e Department of Psychology, The University of Texas at Austin, Austin, TX 78712

f Center for Brains, Minds & Machines

* These authors equally contributed to this work.

Supplementary Table1.

AREA	Talairach		KOLSTER Talairach	
	**LH**	**RH**	**LH**	**RH**
MT+	-48 -74 6	48 -60 6	-48 -75 3	46 -78 6
phPIT	-42 -73 -11	45 -75 -10	-40 -85 -6	42 -85 -9
LOC	-48 -66 -4	56 -64 -8	-42 -89 -2	40 -91 -3
			-36 -90 4	36 -92 3
PHA	-22 -42 -12	28 -42 -6	n.a.	n.a.
Faces1	-44 -70 -14	48 -74 -12	n.a.	n.a.
Faces2	-42 -48 -18	42 -42 -22	n.a.	n.a.
TPJ	-42 -76 37	57 -64 31	n.a.	n.a.

Supplementary Table 1 – related to figure 3: Average Talairach (MNI) coordinates of the center of seven sets of functionally defined areas. Average Talairach coordinates (MNI) of the center of areas lobe in the left and right hemispheres of the temporal lobe defined thought the attention task (phPIT) and localizers (motion: MT+; object: LOC; places: PHA; faces: FACES1 and 2). For comparison, Talaraich coordinates of the same areas defined in a recent retinotopic experiment are also shown (left columns)[1]. MT+: middle temporal complex; phPIT, putative human posterior infero-temporal area; LOC: latera occipital complex; PHA: parahippocampal area; Faces1-2: face area 1-2; TPJ: temporo parietal junction.

Supplementary Table2.

	V1	V2	V3	V3A	V3B	V4	V4t	MT	MST	FST	**PIT**	FFC	PHA1	PHA2	PHA3	LO1	LO2	LO3	LIPv	LIPd	IPS1
**Attention**	0.54	0.54	0.28	0.11	0.08	0.36	**0.02**	0.06	0.17	0.08	**0.02**	**0.04**	**0.53**	0.23	0.43	**0.01**	0.06	0.14	0.19	0.29	0.34
**Motion (10^{-3})**	**0.00**	**0.00**	**0.00**	**0.00**	**0.01**	**0.04**	**0.00**	**0.00**	**0.00**	**0.00**	**0.01**	**0.10**	**2.47**	**1.24**	**5.84**	**0.00**	**0.11**	**0.00**	**0.32**	**3.33**	**0.41**
**Static (10^{-2})**	**0.00**	**0.00**	**0.00**	0.17	**0.00**	**0.00**	0.43	0.57	0.75	0.13	**0.00**	0.10	0.45	0.84	0.31	**0.00**	**0.01**	0.15	0.61	0.87	0.54
**Shapes**	0.63	0.63	0.58	0.37	0.61	0.83	**0.00**	**0.01**	**0.03**	**0.02**	0.62	**0.03**	**0.03**	**0.02**	0.09	0.89	**0.00**	0.92	0.55	0.76	0.88

Supplementary Table 2 – related to figure 5. p-values for the statistics testing significance of response differences in the attention task (attended vs. unattended; one-sided paired t-test, uncorrected for multiple comparisons), motion localizer (moving vs. static stimuli; one-sided paired t-test, uncorrected for multiple comparisons), responsivity to static stimuli (one-sided t-test, uncorrected for multiple comparisons) and shape localizer (faces, scenes, objects; one-way ANOVA uncorrected for multiple comparisons). Significant effects are indicated in bold ($p<0.05$). Please note p-values < 10^{-5} (for motion), 10^{-4} (for static), 10^{-2} (for shapes) are indicated as 0. V1-2-3-3A-3B-4: visual areas 1-2-3-3A-3B-4; V4t: visual area 4 transition; MT+: middle temporal: MST: middle superior temporal; FST: fundus superior temporal; phPIT: putative human posterior infero-temporal area; FCC: fusiform face complex; PH1-2-3, para-hippocampal area 1-2-3; LO1-2-3: lateral occipital areas 1-2-3; LIPv: ventral latera intraparietal area; LIPd: dorsal latera intraparietal area; IPS1: intra parietal sulcus 1; other conventions as in Supplementary Table 1.

Supplementary Table3.

Application	Github repository	Open Service DOI
**DATA PREPARATION**		
Bvec Normalization	https://github.com/brain-life/app-datanormalize	https://doi.org/10.25663/bl.app.4
T1 AC-PC Alignment	https://github.com/brain-life/app-acpcART	https://doi.org/10.25663/bl.app.16
Register to T1	https://github.com/brainlife/app-dtiinit	https://doi.org/10.25663/bl.app.3
dMRI Shell Splitting	https://github.com/brain-life/app-splitshells	https://doi.org/10.25663/bl.app.17
NODDI Fit via Amico	https://github.com/brain-life/app-noddi-amico	https://doi.org/10.25663/bl.app.35
**ROI**		
Multi-Atlas Transfer Tool	https://github.com/faskowit/app-multiAtlasTT	https://doi.org/10.25663/bl.app.23
ROI Generation Tool	https://github.com/brain-life/app-roiGenerator	https://doi.org/10.25663/bl.app.37
**TRACTOGRAPHY**		
ROI to ROI Ensemble Tractography	https://github.com/brain-life/app-roi2roitracking	https://doi.org/10.25663/bl.app.34
Ensemble Tractography	https://github.com/brain-life/app-ensembletracking	https://doi.org/10.25663/bl.app.33
Remove Tract Outliers	https://github.com/brainlife/app-AFQclean	https://doi.org/10.25663/bl.app.11
Tract Profiles	https://github.com/brain-life/app-tractanalysisprofiles	https://doi.org/10.25663/bl.app.43
Tract Statistics	https://github.com/kitchell/app-classifiedfibertractstats	https://doi.org/10.25663/bl.app.12
Linear Fascicle Evaluation	https://github.com/brainlife/app-life	https://doi.org/10.25663/bl.app.1
White matter Tract Segmentation	https://github.com/brain-life/app-wmaSeg	https://doi.org/10.25663/bl.app.41
Generate tract endpoint maps	https://github.com/brainlife/app-endpointMapGeneration/tree/1.0	https://doi.org/10.25663/brainlife.app.194
**CONTROLS**		
Attention ROI Warp	https://github.com/brainlife/Attention_ROI_warp	https://doi.org/10.25663/brainlife.app.168

Supplementary Table 3 – related to figure 6 and 7. Description and web-links to the open source code and open cloud services used in the creation and analysis of the diffusion MRI dataset.

Supplementary Table 4.

CONTROL PROPERTY	Theoretical proposals [2]	FEF (2)	LIP (3)	phPIT (4)	TPJ (5)	V1 (6)
Activation during prolonged endogenous attention	✓	✓	✓	✓	x	x
Independence of specific visual features	✓	✓	✓	✓	✓	x
Causal relationship with attentive behavior/state	✓	✓	✓	✓	n.a.	x
Sustained neuronal response for attention signals	✓	✓	✓	✓	n.a.	x
Neuropsychological evidence	n.a.	✓	✓	✓	✓	x

Supplementary Table 4– related to figure 3, 4, and discussion. A checklist of functional properties required to define an attention control area is shown for dorsal attention nodes FEF (frontal eye fields) and LIP (lateral intra-parietal area), for phPIT (putative human posterior infero-temporal area)), and for TPJ (temporo parietal junction) and V1 (visual area 1) for comparison. Example references are provided below: (1)[2]; (2)[2–7] (3)[4,5]; (4)[1,8–12] (5)[13,14] (6)[15–18].

Supplementary Figure 1

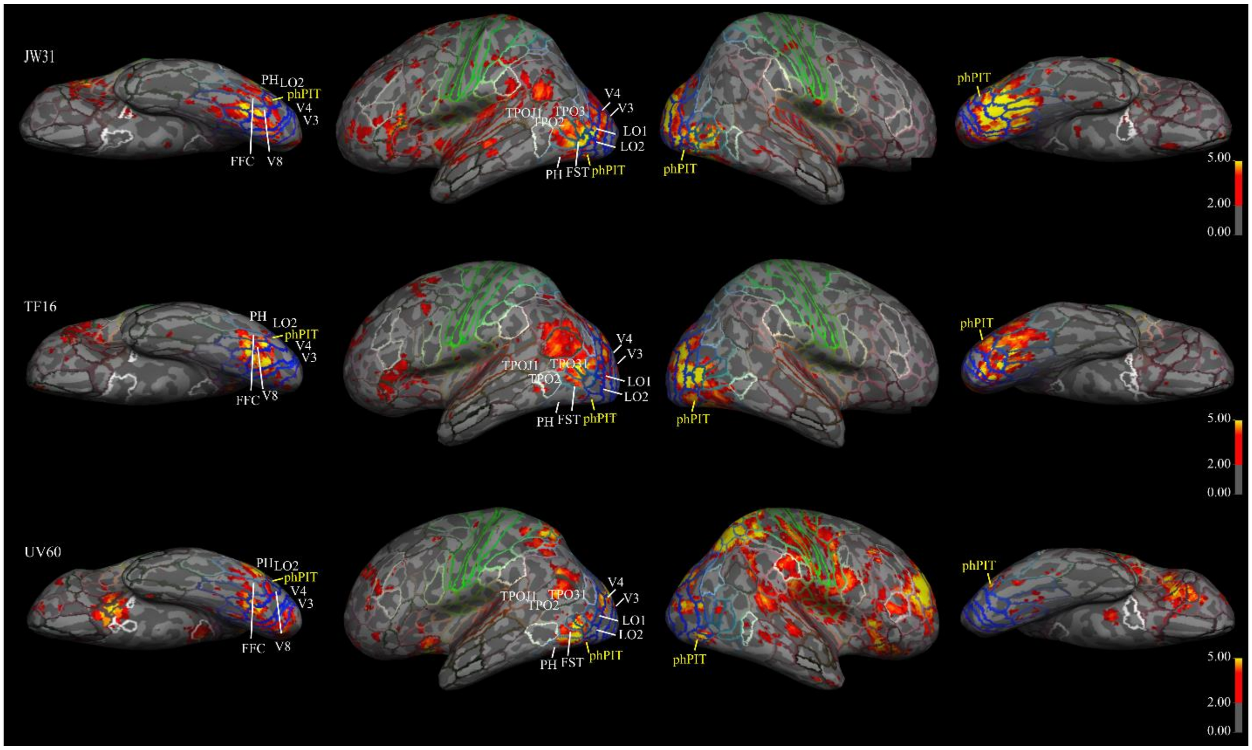

Supplementary Figure 1 – related to Figure 3. Activation for the attentive motion discrimination for three individual subjects. Statistical parametric maps of the contrast 'attend contralaterally versus ipsilaterally' overlaid on the inflated brains of three single subjects. Task-related activations (yellow/red) are shown on lateral and inferior views of the brain and superimposed on the Glasser atlas parcellation [19]. As expected, attention modulated early visual areas like V3, V4, and motion areas MT, MST and FST. Surprisingly attention, activated phPIT and V8 area. The color-bar shows T-values task-related activations. FFC: fusiform face area; FST: fundus superior temporal area; LO1-2: lateral occipital areas 1-2; PH: basal temporo-occipital area; phPIT: putative human posterior infero-temporal area; TPOJ-1-2-3: temporo parietal occipital junction 1-2-3; V2-3-8: visual areas 2-3-8.

Supplementary Figure 2

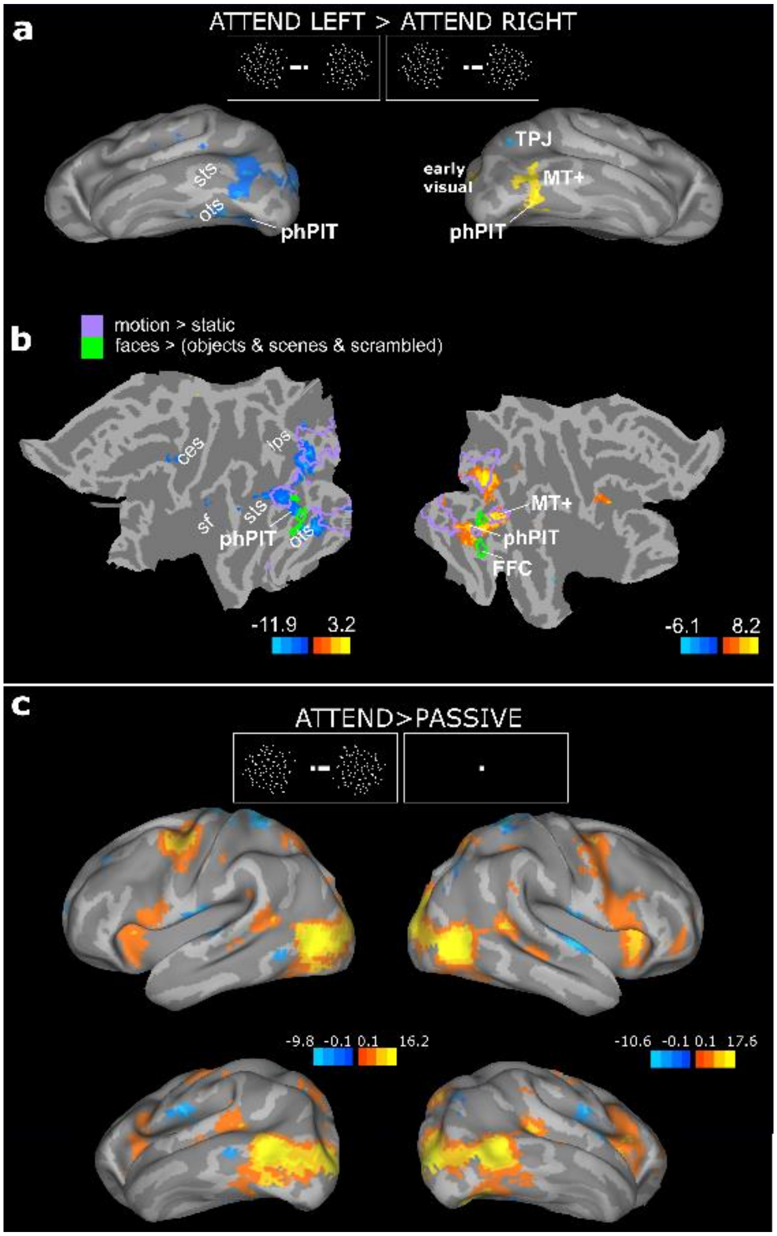

Supplementary Figure 2 – related to Figure 3-4. phPIT functional profile random effect group analysis. **a.** Statistical parametric maps of the contrast ‘attend left versus right’ for the random effect group analysis overlaid on the inferior views of the average human inflated brain. **b.** Statistical parametric maps of the contrast ‘attend left versus right’ for the random effect group analysis overlaid on flat map of the left and right hemispheres; solid lines show visual selectivity for motion (purple) and faces (green). The color-bar shows T-values task-related activations and inactivations. c. Statistical parametric maps of the contrast ‘attention (ATTEND) vs. passive fixation (PASSIVE)’

overlaid on the lateral and inferior views of the inflated average human brain. The color-bar shows T-values task-related activations (yellow/red) and inactivation (blue). ces: central sulcus; ips: intra-parietal sulcus; ots: Occipito-temporal sulcus; sf: Sylvian Fissure; sts: superior temporal sulcus; FFC: fusiform face area; MT+: middle temporal area; phPIT: putative human posterior infero-temporal area; TPJ: temporo-parietal junction; other conventions as in Supplementary Table 1-2.

Supplementary Figure 3

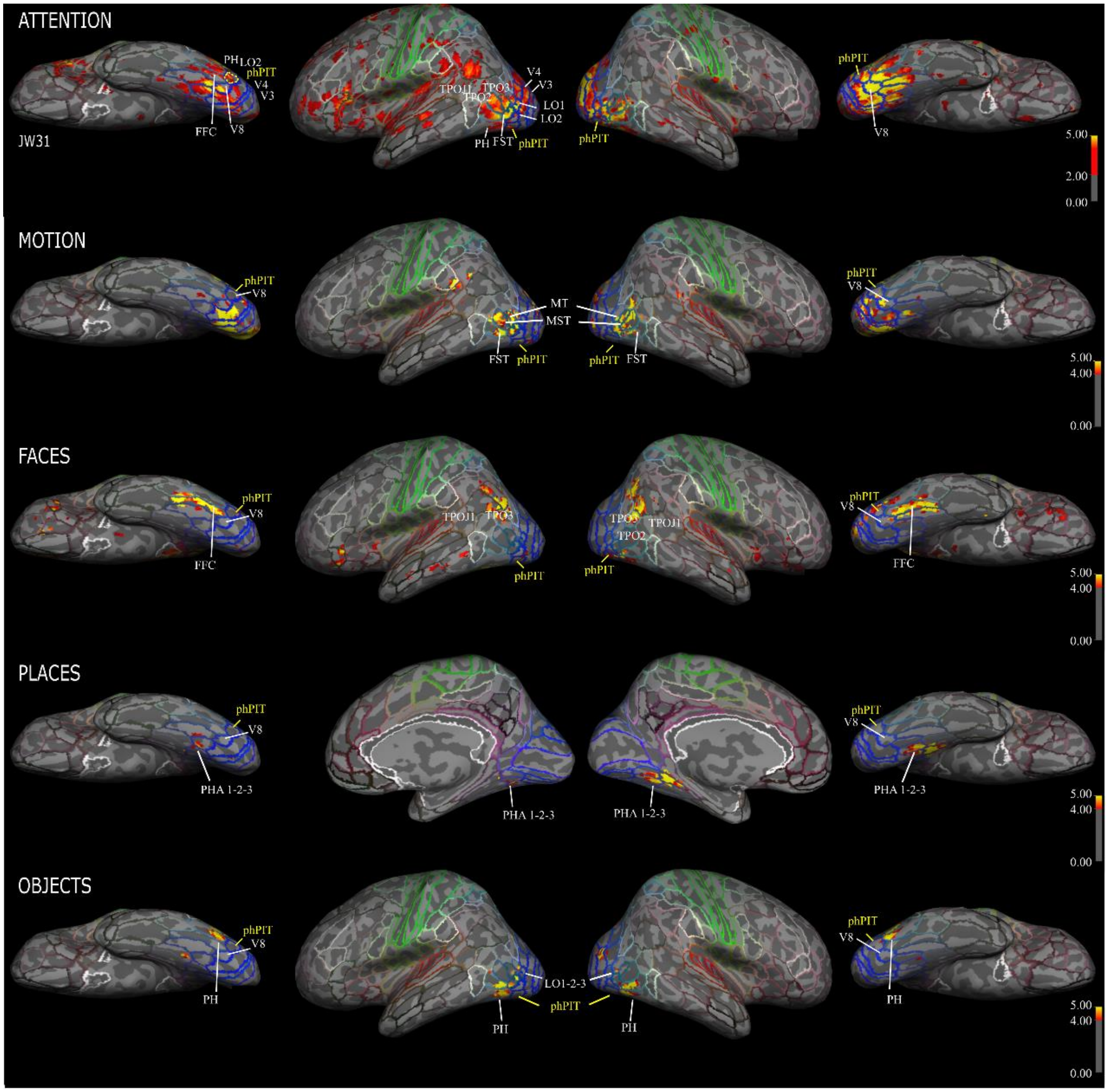

Supplementary Figure 3 – related to Figure 3-4. Individual subject functional characterization of phPIT. Statistical parametric maps of attention and four different localizers overlaid on the inflated brain of a single subject. Task-related activations (yellow/red) are shown on both lateral and inferior views and superimposed on the Glasser atlas parcellation [19]. Early visual areas like V3, V4, and motion areas MT, MST and FST were modulated by attention, but also strongly activated by moving stimuli, the task relevant dimension, while being unresponsive to other higher order visual stimuli. Higher order visual areas like FFC, PPA, LOC and PH[19] were activated by faces, places, and

objects respectively, but not by attention. Critically, phPIT and area V8, were activated by attention but were not selective for other visual stimuli suggesting a general role in attention. The color-bar shows T-values task-related activations. FFC: fusiform face area; FST: fundus superior temporal area; LO1-2: lateral occipital areas 1-2; MT: middle temporal area; MST: middle superior temporal area; PH: basal temporo-occipital area; phPIT: putative human posterior infero-temporal area; TPOJ-1-2-3: temporo parietal occipital junction 1-2-3; V8: visual area 8.

Supplementary Figure 4

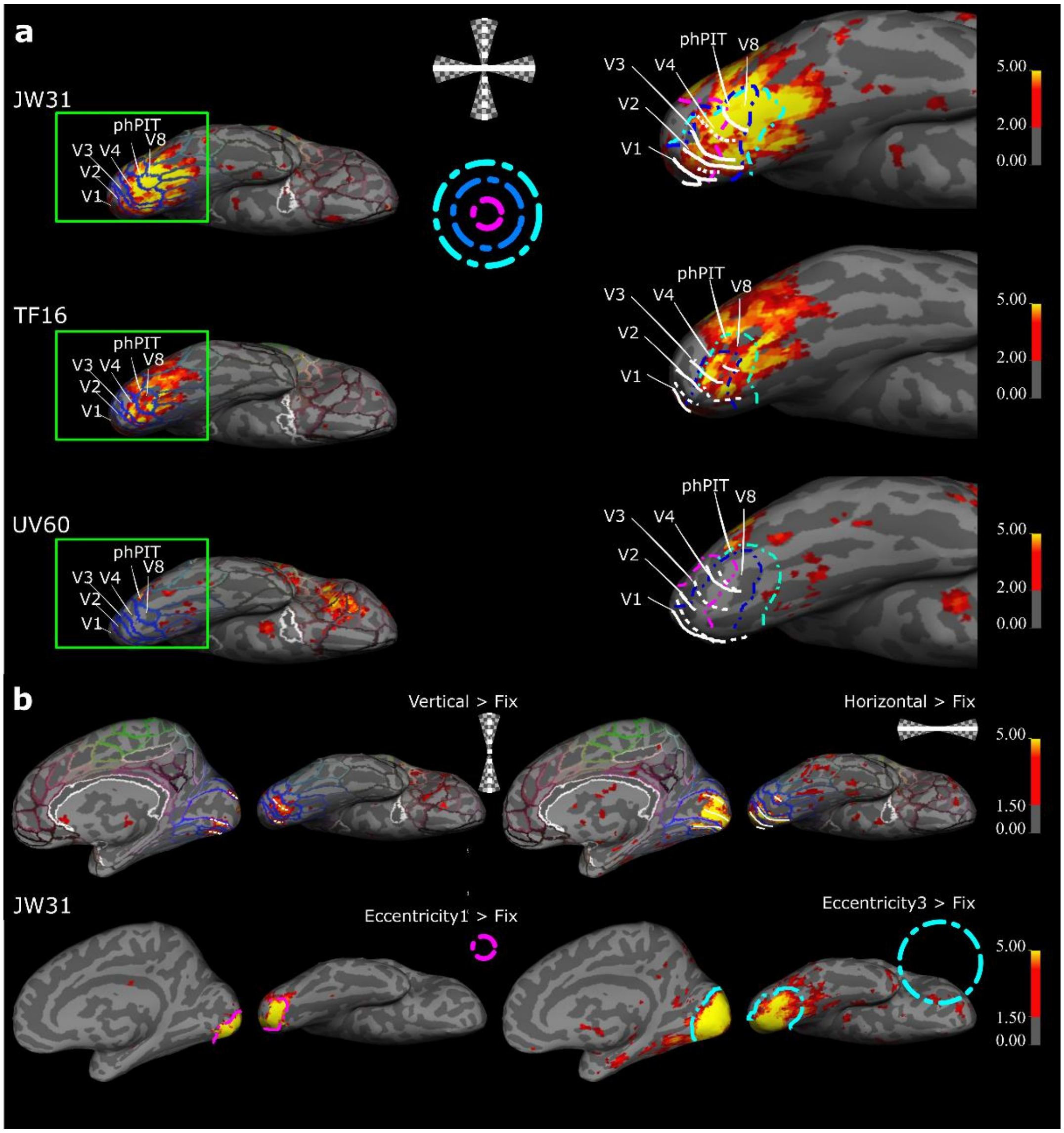

Supplementary Figure 4 – related to Figure 3. Individual subject retinotopic and eccentricity characterization of attentional activation in phPIT. A. Statistical parametric maps of attention superimposed with meridians and eccentricity boundaries are overlaid on the inflated brain of three single subjects. Task-related activations (yellow/red) are shown on the inferior views and superimposed on the Glasser atlas parcellation[19] (first column) and with retinotopic and eccentricity mapping (second column – enlarged view). Full and dotted white lines indicate horizontal and vertical meridians respectively; colored dashed lines show positions of central, intermediate and peripheral eccentricity ridges. The combination of retinotopic and eccentricity mapping better clarified the separation between

the infero-temporal activation and that of early visual areas, and of other specialized areas located more anteriorly in the temporal lobe. In individual subjects we were able to identify the attentional activation in the infero-temporal cortex as the most anterior and ventral region possessing a retinotopic organization, but not strong motion selectivity, nor complex object selectivity. In Glasser nomenclature phPIT corresponded to phPIT and V8 areas. **B.** Statistical parametric maps of retinotopic (top, vertical and horizontal wedge) and eccentricity (bottom, inner and outer ring) for a representative subject. The color-bar shows T-values task-related activations. phPIT: putative human posterior infero-temporal area; V1-2-3-84-: visual areas 1-2-3-4-8.

Supplementary Figure 5

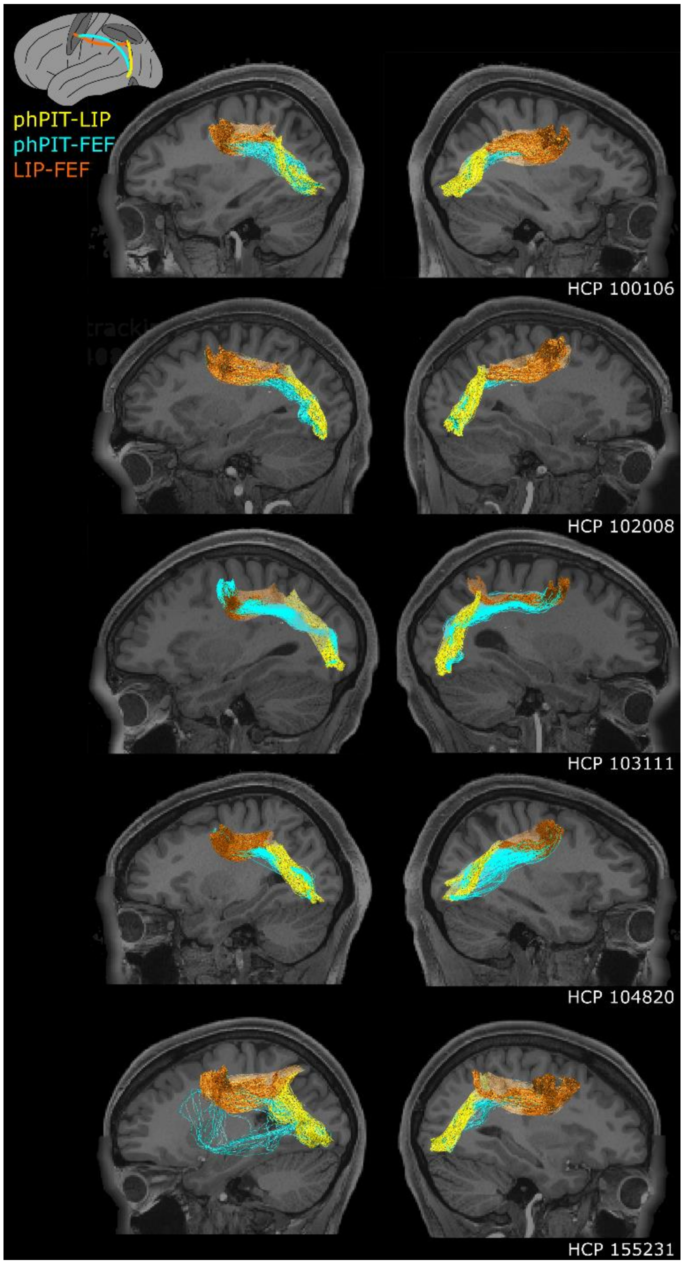

Supplementary Figure 5 – related to Figure 5. Core of the tract. Sagittal-view of phPIT-to-LIP (yellow), phPIT-to-FEF (cyan), and LIP-to-FEF (orange) connections overlaid on T1 image for five example subjects from the HCP. The core of the tract was consistent across subjects. For about 20% of the subjects the phPIT-to-FEF showed some branching (lowest row). FEF: frontal eye field; LIP: lateral intra parietal; phPIT: putative human posterior infero-temporal area. Source data are available at https://doi.org/10.25663/BRAINLIFE.PUB.17.

Supplementary Figure 6

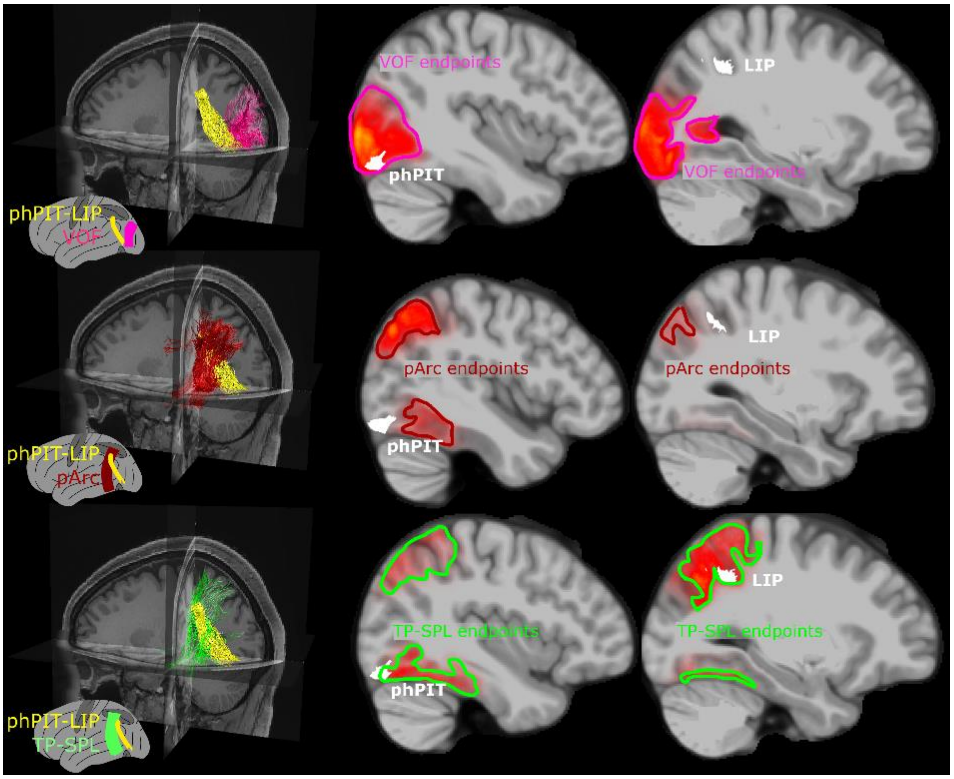

Supplementary Figure 6 – related to Figure 7. An unreported sup-portion of the pArct supports the endogenous attention network. Different views of phPIT-to-LIP connections (yellow) and nearby anatomical tracts VOF (pink), pArc (dark red) and TP-SPL (green) overlaid on T1 image for a single example subject from the HCP (101006). phPIT-to-LIP pathway departs from that of traditionally segmented tracts. Right panels show density mapping of the superior and inferior cortical endpoints for the anatomical tracts. Density projections are summed across 1000 HCP subjects; darker coloring of the heat map corresponds to higher densities. White areas correspond to the ROIs used to generate the attention tracts. LIP, Lateral Intraparietal area; phPIT, putative human Posterior Infero-Temporal area; pArc, posterior Arcuate Fasciculus; TP-SPL; Temporo-Parietal connection to the Superior Temporal Lobule; VOF, Vertical Occipital Fasciculus. Source data are available at https://doi.org/10.25663/BRAINLIFE.PUB.17.

Supplementary Figure 7 – related to Figure 5. Tracking results with control ROIs.

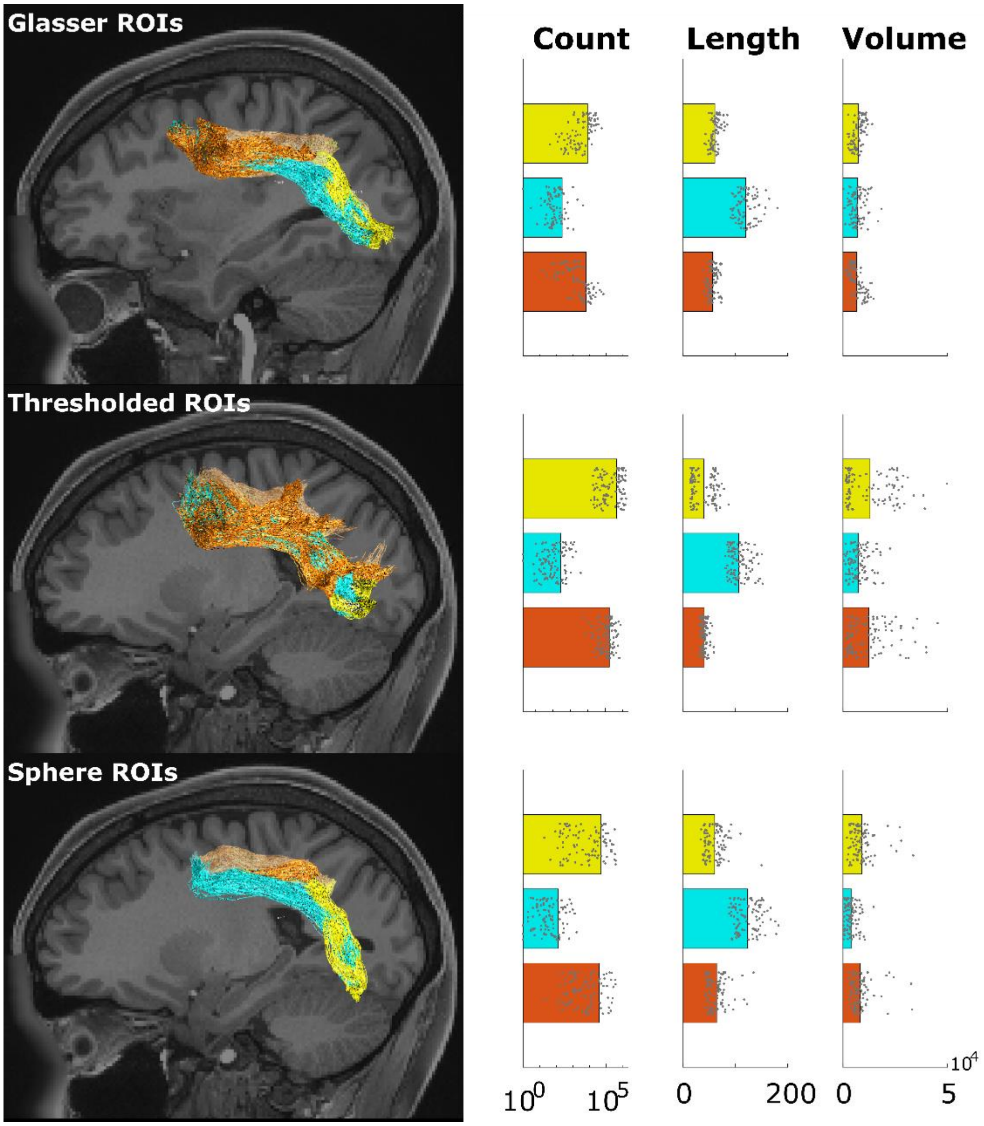

Supplementary Figure 7 – related to Figure 5. Tracking results with control regions of interest (ROIs). Left column shows sagittal-views of phPIT-to-LIP (yellow), phPIT-to-FEF (cyan), and LIP-to-FEF (orange) connections overlaid on T1 image for an example subjects from the HCP. The core and pathway of the tracts were consistent even when tracking was performed with Glasser ROIs, thresholded ROIs and sphere ROIs (see Methods). Right column shows average streamline number, tract length (mm), tract volume (mm^3) are shown for each functional tract for the three different tracking Methods. Data are expressed as mean across 50 subjects and collapsed across hemispheres; gray points represent the values for each individual subject and hemisphere. FEF: frontal eye field; LIP: lateral intra parietal; phPIT: putative human posterior infero-temporal area. Source data are provided as a Source Data file.

References

1. Kolster, H., Peeters, R. & Orban, G. A. The retinotopic organization of the human middle temporal area MT/V5 and its cortical neighbors. *J. Neurosci.* **30,** 9801–9820 (2010).

2. Fecteau, J. H. & Munoz, D. P. Salience, relevance, and firing: a priority map for target selection. *Trends Cogn. Sci. (Regul. Ed.)* **10,** 382–390 (2006).

3. Rossi, A. F., Bichot, N. P., Desimone, R. & Ungerleider, L. G. Top down attentional deficits in macaques with lesions of lateral prefrontal cortex. *J. Neurosci.* **27,** 11306–11314 (2007).

4. Kastner, S. & Ungerleider, L. G. Mechanisms of visual attention in the human cortex. *Annu. Rev. Neurosci.* **23,** 315–341 (2000).

5. Corbetta, M. & Shulman, G. L. Control of goal-directed and stimulus-driven attention in the brain. *Nat. Rev. Neurosci.* **3,** 201–215 (2002).

6. Bichot, N. P., Heard, M. T., DeGennaro, E. M. & Desimone, R. A Source for Feature-Based Attention in the Prefrontal Cortex. *Neuron* **88,** 832–844 (2015).

7. Moore, T. & Armstrong, K. M. Selective gating of visual signals by microstimulation of frontal cortex. *Nature* **421,** 370–373 (2003).

8. Stemmann, H. & Freiwald, W. A. Evidence for an attentional priority map in inferotemporal cortex. *Proc. Natl. Acad. Sci. USA* **116,** 23797–23805 (2019).

9. Stemmann, H. & Freiwald, W. A. Attentive motion discrimination recruits an area in inferotemporal cortex. *J. Neurosci.* **36,** 11918–11928 (2016).

10. Kolster, H., Janssens, T., Orban, G. A. & Vanduffel, W. The retinotopic organization of macaque occipitotemporal cortex anterior to V4 and caudoventral to the middle temporal (MT) cluster. *J. Neurosci.* **34,** 10168–10191 (2014).

11. Bogadhi, A. R., Bollimunta, A., Leopold, D. A. & Krauzlis, R. J. Spatial attention deficits are causally linked to an area in macaque temporal cortex. *Curr. Biol.* **29,** 726–736.e4 (2019).

12. Bogadhi, A. R., Bollimunta, A., Leopold, D. A. & Krauzlis, R. J. Brain regions modulated during covert visual attention in the macaque. *Sci. Rep.* **8,** 15237 (2018).

13. Doricchi, F., Macci, E., Silvetti, M. & Macaluso, E. Neural correlates of the spatial and expectancy components of endogenous and stimulus-driven orienting of attention in the Posner task. *Cereb. Cortex* **20,** 1574–1585 (2010).

14. Shulman, G. L., Astafiev, S. V., McAvoy, M. P., d Avossa, G. & Corbetta, M. Right TPJ deactivation during visual search: functional significance and support for a filter hypothesis. *Cereb. Cortex* **17,** 2625–2633 (2007).

15. Serences, J. T. & Boynton, G. M. Feature-based attentional modulations in the absence of direct visual stimulation. *Neuron* **55,** 301–312 (2007).

16. Buracas, G. T. & Boynton, G. M. The effect of spatial attention on contrast response functions in human visual cortex. *J. Neurosci.* **27,** 93–97 (2007).

17. Maunsell, J. H. R. & Cook, E. P. The role of attention in visual processing. *Philos. Trans. R. Soc. Lond. B, Biol. Sci.* **357,** 1063–1072 (2002).

18. Reynolds, J. H. & Chelazzi, L. Attentional modulation of visual processing. *Annu. Rev. Neurosci.* **27,** 611–647 (2004).

19. Glasser, M. F. *et al.* A multi-modal parcellation of human cerebral cortex. *Nature* **536,** 171–178 (2016).
