# Supplementary material for: The human endogenous attentional control network includes a ventro-temporal cortical node
Source: Nat Commun. 2021 Jan 15;12:360. doi: 10.1038/s41467-020-20583-5 (PMC7810878; doi:10.1038/s41467-020-20583-5)
Supplement: Supplementary file 2 — Reporting Summary [file 41467_2020_20583_MOESM2_ESM.pdf]

nature research

Corresponding author(s): Ilaria Sani

Last updated by author(s): Oct 22, 2020

Reporting Summary

Nature Research wishes to improve the reproducibility of the work that we publish. This form provides structure for consistency and transparency in reporting. For further information on Nature Research policies, see Authors & Referees and the Editorial Policy Checklist .

Statistics

For all statistical analyses, confirm that the following items are present in the figure legend, table legend, main text, or Methods section.

n/a	Confirmed	
☐	☒	The exact sample size (n) for each experimental group/condition, given as a discrete number and unit of measurement
☐	☒	A statement on whether measurements were taken from distinct samples or whether the same sample was measured repeatedly
☐	☒	The statistical test(s) used AND whether they are one- or two-sided<br>*Only common tests should be described solely by name; describe more complex techniques in the Methods section.*
☒	☐	A description of all covariates tested
☐	☒	A description of any assumptions or corrections, such as tests of normality and adjustment for multiple comparisons
☐	☒	A full description of the statistical parameters including central tendency (e.g. means) or other basic estimates (e.g. regression coefficient) AND variation (e.g. standard deviation) or associated estimates of uncertainty (e.g. confidence intervals)
☐	☒	For null hypothesis testing, the test statistic (e.g. F, t, r) with confidence intervals, effect sizes, degrees of freedom and P value noted<br>*Give P values as exact values whenever suitable.*
☒	☐	For Bayesian analysis, information on the choice of priors and Markov chain Monte Carlo settings
☒	☐	For hierarchical and complex designs, identification of the appropriate level for tests and full reporting of outcomes
☒	☐	Estimates of effect sizes (e.g. Cohen's d, Pearson's r), indicating how they were calculated

Our web collection on statistics for biologists contains articles on many of the points above.

Software and code

Policy information about availability of computer code

Data collection	A custom made eye tracking system was used to control and record eye movements during experiments. The visual stimuli were generated by a custom made presentation software 'Visiko', which also recorded eye and behavioral data. Functional MRI data was acquired in a Siemens Allegra 3T system.
Data analysis	Functional MRI data were analyzed using SPM8 (statistical parametric mapping, Wellcome Departement of Imaging Neuroscience; London, UK) under Matlab R2010a. Marsbar (region of interest toolbox for SPM version 0.44). was used for ROI analysis. Data visualization was done in caret5 (http://www.nitrc.org/projects/caret/). Diffusion MRI data were analyzed using the online platform brainlife.io, links to the code are provided in the text. The following software were used: MRtrix version 0.2.12 (Tournier et al., 2012) for tracking, FreeSurfer tools version 6.0.0, Matlab R2019a, Trackvis version 0.6.1 for visualization. The datasets, along with their provenance (i.e. the pipeline) and the links to the applications used to generate them are available at https://doi.org/10.25663/BRAINLIFE.PUB.16 for the main diffusion analyses and at https://doi.org/10.25663/BRAINLIFE.PUB.17 for the control analyses. Individual applications within the pipeline are explained in the method sections and individual links to the onlie Apps and to the correspondent github repository are also provided in supplementary table 3.

For manuscripts utilizing custom algorithms or software that are central to the research but not yet described in published literature, software must be made available to editors/reviewers. We strongly encourage code deposition in a community repository (e.g. GitHub). See the Nature Research guidelines for submitting code & software for further information.

Data

Policy information about availability of data

All manuscripts must include a data availability statement. This statement should provide the following information, where applicable:

- Accession codes, unique identifiers, or web links for publicly available datasets
- A list of figures that have associated raw data
- A description of any restrictions on data availability

Source data for fMRI figures are enclosed to the manuscript as an excell file. For diffusion MRI analyses data source for the main experiment are available here:

https://doi.org/10.25663/BRAINLIFE.PUB.16. Data source for the control experiment are available here https://doi.org/10.25663/BRAINLIFE.PUB.17. Other data potentially useful to the reader will be available form the authors upon requests.

Field-specific reporting

Please select the one below that is the best fit for your research. If you are not sure, read the appropriate sections before making your selection.

☒ Life sciences ☐ Behavioural & social sciences ☐ Ecological, evolutionary & environmental sciences

For a reference copy of the document with all sections, see nature.com/documents/nr-reporting-summary-flat.pdf

Life sciences study design

All studies must disclose on these points even when the disclosure is negative.

Sample size	Functional data during performance of the attention task (as well as for the localizers) were acquired from 12 subjects. Sample size and experimental approach was chosen after Kolster and colleagues (2010) who localized phPIT successfully and consistently across subjects (11 participants) by using several tasks and localizers. Further details about sample size are provided in the method section.
Data exclusions	No data were excluded from the analyses
Replication	Analysis were performed at the population level and results were then replicated across individuals. The variations in individual subjects responses was incorporated into statistical testing. The variability in responses across subjects are shown in several supplementary figure and are indicated in each relevant plot with standard errors. Moreover, we related the results to publicly available atlases and previous literature: two independent groups have found consistent results in a different animal species (macaques) and another group isolated the same area with a different approach in humans (Kolster et al., 2010). Code/data/stimuli will available upon request to allow further reproducibility.
Randomization	Randomization procedures were not applied because the study focuses on one group of participants
Blinding	Blinding procedures were not applied because the study focuses on one group of participants

Reporting for specific materials, systems and methods

We require information from authors about some types of materials, experimental systems and methods used in many studies. Here, indicate whether each material, system or method listed is relevant to your study. If you are not sure if a list item applies to your research, read the appropriate section before selecting a response.

Materials & experimental systems

n/a	Involved in the study	
☒	☐	Antibodies
☒	☐	Eukaryotic cell lines
☒	☐	Palaeontology
☒	☐	Animals and other organisms
☐	☒	Human research participants
☒	☐	Clinical data

Methods

n/a	Involved in the study	
☒	☐	ChIP-seq
☒	☐	Flow cytometry
☐	☒	MRI-based neuroimaging

Human research participants

Policy information about studies involving human research participants

Population characteristics	Twelve volunteers participated in the fMRI experiment (4 females, 6 males; mean age, 23 years +/- 2 years). All participants had normal or corrected-to-normal visual acuity, and no history of mental illness or neurological diseases. Prior to the experiment all volunteers were tested on the Freiburg Visual Acuity Test, the Titmus-Test for stereopsis, and the Ishihara-Test for color-vision.
Recruitment	All subjects were recruited from students of psychology and biology at the University of Bremen. The recruitment of young students was particularly important given the challenging nature of the task, which involved the use of eye movements within the scanner in a multi-session experiment. The recruitment of college students is common in neuroimaging studies and we do not foresee any specific bias in the results.
Ethics oversight	The study was approved by the ethics committee of the University of Bremen, and all volunteers gave written consent in accordance with the Helsinki declaration before the experiment.

Note that full information on the approval of the study protocol must also be provided in the manuscript.

Magnetic resonance imaging

Experimental design

Design type	Rapid Serial Visual Presentation (RSVP) task; motion discrimination task; block design
Design specifications	Each participant performed the task for 18 minutes in alternating blocks; each block consisted in 7 successful trials and approximately 30s; task blocks were separated by 5s of passive fixation
Behavioral performance measures	Subject saccades to visual targets were recorded; the task had 5 main behavioral outcomes; performance was assessed by calculating the mean and standard deviation across subjects;

Acquisition

Imaging type(s)	functional MRI; diffusion MRI from Human Connectome Project
Field strength	3T
Sequence & imaging parameters	Functional time series consisted of single-shot echo-planar images (EPI): repetition time (TR) 2.51s, echo time (TE) 30ms, flip angle 85 deg, field of view 192x192mm and 3 x 3 x 2.7 mm3 voxel size (38 slices).
Area of acquisition	whole brain scan

Diffusion MRI ☒ Used ☐ Not used

Parameters: We used data from HCP; The HCP data were acquired at multiple b-values (b = 1000, 2000 and 3000 s/mm2) and 90 diffusion directions per gradient strength, with 1.25 mm isotropic resolution.

Preprocessing

Preprocessing software	Functional scans were slice-time- and motion- corrected, spatially smoothed with a Gaussian kernel of 7mm full-width-at-half-max and finally co-registered and warped to SPM8 standard space. Diffusion data were preprocessed by WU-Minn HCP 543 Consortiums using methods that are described in (Sotiropoulos et al., 2013).
Normalization	The standard normalization built-in procedure of SPM8 was used.
Normalization template	ICBM template
Noise and artifact removal	A 6 DOF (degree of freedom) rigid body motion correction was applied. A correction of heart rate or respiration has not been applied.
Volume censoring	No volume censoring was applied.

Statistical modeling & inference

Model type and settings	A fixed effects model analysis was used
Effect(s) tested	An ANOVA revealed an increased activity in motion selective cortical areas as well as in areas of the frontal and parietal lobe in trials in which the stimulus in the contra lateral hemifield was attended. Intriguingly also a temporal area showed an increased activity for this condition

Specify type of analysis: ☐ Whole brain ☐ ROI-based ☒ Both

Anatomical location(s): The Glasser atlas (Glasser et al., 2016) was used for brain parcellation.

Statistic type for inference (See Eklund et al. 2016)	Voxel-wise inference - uncorrected p-values.
Correction	Activation during active attention task was contrasted with fixation task and shown at a significance level of p<0.001 FWE (family-wise error)

Models & analysis

n/a	Involved in the study
☒	☐ Functional and/or effective connectivity
☒	☐ Graph analysis
☒	☐ Multivariate modeling or predictive analysis
