# Supplementary material for: The human endogenous attentional control network includes a ventro-temporal cortical node
Source: Nat Commun. 2021 Jan 15;12:360. doi: 10.1038/s41467-020-20583-5 (PMC7810878; doi:10.1038/s41467-020-20583-5)
Supplement: Supplementary file 3 — Peer Review File [file 41467_2020_20583_MOESM3_ESM.pdf]

Editorial Note: Parts of this Peer Review File have been redacted as indicated to maintain the confidentiality of unpublished data.

Reviewers' comments:

Reviewer #1 (Remarks to the Author):

The functional characterisation of human brain areas is making slow signs of progress using the combination of direct electrical recordings in non-human primates and advanced neuroimaging in humans.
Within that context, Sani et al. used multimodal imaging to localise in humans an attentional area well known in non-human primates, the dorsal portion of posterior inferotemporal cortex (PITd). To do so, Sani et al. used the same attentional paradigm used in macaques and n= 10 humans to define homologies and further used white matter connectivity to confirm homologies in term of connectivity.
Overall I think this is a valuable paper, which, after substantial revision, will become a reference in the field of attention, neuroimaging, clinical neuroanatomy and comparative anatomy.

General comment.
The use of the term "attention control" as a function, is confusing. Decades of work on attention dissociated attention into spatial attention, feature-specific attention, endogenous, exogenous, preparative. The authors need to clarify exactly the functions they intend to map onto the brain.

The use of 10 participants is really small as a sample compared to other studies and should be explained/acknowledged.

Methods comments
Part of the argument of the authors is to demonstrate that the region reported is attention specific. Being attention specific (not tested in the study) is different from being motion and shape independent (actually tested in the study). Why doesn't another attention paradigm not using motion activate the dorsal portion of posterior inferotemporal cortex? Would it be possible that the dorsal part of the posterior inferotemporal cortex would be specific to the attention to motion? Besides recent work published by Chen, Wasserman et al. Nature Communications 2020 "The visual word form area (VWFA) is part of both language and attention circuitry" stress that a region can be located at the interplay between two systems. Hence, the mapping of function to brain regions is not systematically a 1 to 1. I struggled with this point in the manuscript.

Discussion/interpretation comments.
"The control of endogenous attention has traditionally been attributed, with much support, to a fronto-parietal network (Corbetta et al., 2008; Gottlieb et al., 1998; Kastner and Ungerleider, 2000; Thompson and Bichot, 2005). Our results suggest that there is a novel area of attention control, located in a specific part of the temporal lobe, phPIT, and that this area forms a node in the endogenous attention control network"
This is inaccurate. The areas reported by the authors has been discussed by many authors including Corbetta and Shulman and rejected based on information derived from electrophysiology "Areas in the occipital lobe (fusiform and MT+) respond transiently to the cue, whereas areas in the dorsal posterior parietal cortex along the intraparietal sulcus (IPs) and in the frontal cortex (at or near the putative human homologue of the FRONTAL EYE FIELD, FEF) show a more sustained response." (Corbetta and Shulman 2002). Further, an area matching phPIT has been reported in Corbetta et al. Nature Neuroscience (2005).

"A previously uncharacterized vertical pathway connects phPIT and dorsal attention area LIP". There is already a confusion between the anterior portion of the vertical occipital fasciculus and the posterior portion of the posterior segment of the arcuate fasciculus. I would suggest the authors to describe this connection as a sub-portion of the posterior segment of the arcuate fasciculus. We want to avoid an unnecessary complexification of neuroanatomy.

Concerning the discussion of the anatomy, the description of the occipital projections of the second branch of the superior longitudinal fasciculus / superior fronto-occipital faciculus described by

Forkel et al. "The anatomy of fronto-occipital connections from early blunt dissections to contemporary tractography" in Cortex 2014 might be of interest for the interpretation of the findings.

Additional analyses suggestion

Since the authors found a homology between humans and macaque, it would be interesting to make some comparisons of the connectome of the phPIT between the two species.

Reviewer #2 (Remarks to the Author):

In this ms the authors report the presence of a new attentional control node in the human inferotemporal cortex. Although a functionally and anatomically similar area has been recently described in the monkey brain, this study provides the first fMRI evidence in humans. This inferotemporal area is likely to be involved in representing space in object-centered / allocentric coordinates. The study provides a fine picture of the anatomical localization of this new area and of its anatomical connections with the other major parietal and frontal nodes of the attentional hub. I have no major technical or methodological objections. In what follows I have listed a number of minor point-to-point suggestions and a request for an additional analysis of fMRI data.

Minor points.

Abstract, page 2: some parts of the text could be toned down a bit. Line 19: "Here we challenge this notion .." , could be changed in " Here we expand this notion..".
I would suggest to shorten and separate in two parts the third sentence like this:
"By combining a demanding behavioral paradigm with functional neuroimaging and diffusion tractography, we show that like fronto-parietal attentional areas, the human posterior inferotemporal cortex exhibits significant attentional modulatory activity. This area is functionally distinct from surrounding inferotemporal areas and is directly connected to parietal and frontal attentional regions.

Page 4 line 60 – 61 : The role of TPJ in contextual updating was initially suggested in Doricchi et al. (2010; see also Macaluso and Doricchi, 2013), who provided evidence for the different roles of that the left and right TPJ play in this process: the left TPJ is activated by valid targets that "match" the position pointed by the cue, the right TPJ by invalid targets that "mismatch" the position that was initially pointed by the cue.

Page 4 line 68 – 69 "…human TPJ activity is actually reduced during endogenous attention." : no reference is provided here. The two fMRI studies that showed reduction of TPJ during orienting of endogenous attention are Shulman et al. (2007) and Doricchi et al. (2010). The first study shows that in a RSVP task, the more the moment of target occurrence approaches the more the right TPJ is deactivated. The second study shows that during orienting of spatial attention with endogenous cues, the more cue are predictive of target location the more TPJ is deactivated.

Lines 391-393: "Although similarly direct evidence is lacking in humans, neuropsychological evidence has increasingly pointed to a role of the temporal lobe in attentional control (Karnath et al., 2001)" : I would reccommend caution in reporting this statement because: a) the area initially highlighted by Karnath et al. (2001) was in the central sector of the Superior Temporal Gyrus: this area is faraway from the phPIT and is anterior to the TPJ. Since the authirs have well functionally differentiated their phPIT area fromthe TPJ, I see really no reason to make an analogy between the phPIT and the central STG; b) ensuing anatomical investigations have systematically failed to replicate Karnath et al. findings.

Line 395: The study by Doricchi and Tomaiuolo (2003) is the first study that pointed out the role of Superior Longitudinal Fasciculus disconnection in spatial neglect (not the SMGyrus). This original

finding was confirmed two years later by the neurosurgical inactivation study by Thiebaut de Schotten et al. (2005). This reference should be reported later on, on line 407 were the role of white matter disconnection is currently quoted in the paper.

Line 207 : very minor typo " .. operated by (Glasser et al., 2016) " should be probably rewritten as " .. operated by Glasser et al. (2016) "

Line 213: other minor typo "(LiFE, (Caiafa and Pestilli, 2017; Pestilli et al., 2014)) " should be probably rewritten as "(LiFE: Caiafa and Pestilli, 2017; Pestilli et al., 2014) "

Lines 442-460: In these lines the authors give emphasis to the anatomical and functional differentiation between the TPJ and PITd. In doing this they define the TPJ a "core node" of the ventral exogenous network. Nonetheless, views on the role of the TPJ are rapidly changing. First, this area is also involved in endogneous orienting, as it gets da-activated during cued orienting (see above-mentioned references: Shulman et al., 2007, Doricchi et al., 2009. Second, there is growing evidence that the TPJ is involved in late phases target-processing linked to contextual updating (references: Doricchi et al., 2010; Geng and Vossel, 2013; Macaluso and Doricchi, 2013). Third, the dorsal-ventral dichotomy of attentional control might not fit with the role that the superior parietal lobule might play in exogenous re-orienting to invalidly cued targets (Ptak and Schnider 2011; Vandenberghe et al., 2012; Dragone et al., 2015). In this part of the discussion section, the authors might wish to offer a more articulated view of the functional role of the TPJ.

Major point.

Page 10 lines 162-164: I wonder if finer grain results could be obtained by supplementing the "attend contralateral" vs "attend ipsilateral" contrast with "attend left" vs "attend right" contrasts run separately for trials that required the same directional leftward or righward saccades. This type of contrast would allow, for example, to investigate whether the phPIT areas in each hemisphere show preferential responses for one of the two hemispaces and, in each area, whether there is a preference for attending leftward vs rightward motion or, put in object-centered terms, toward the left or right side of the lateral clouds of dots. Could the authors please address this proposal and eventually enclose these supplementary analyses in their revision of the manuscript ?

References.

de Schotten, M. T., Urbanski, M., Duffau, H., Volle, E., Lévy, R., Dubois, B., & Bartolomeo, P. (2005). Direct evidence for a parietal-frontal pathway subserving spatial awareness in humans. Science, 309(5744), 2226-2228.

Doricchi, F., Macci, E., Silvetti, M., & Macaluso, E. (2010). Neural correlates of the spatial and expectancy components of endogenous and stimulus-driven orienting of attention in the Posner task. Cerebral Cortex, 20(7), 1574-1585.

Dragone A, Lasaponara S, Silvetti M, Macaluso E, Doricchi F. 2015.
Selective reorienting response of the left hemisphere to in- valid visual targets in the right side of space: relevance for the spatial neglect syndrome. Cortex. 65:31–35.

Macaluso, E., & Doricchi, F. (2013). Attention and predictions: control of spatial attention beyond the endogenous-exogenous dichotomy. Frontiers in human neuroscience, 7, 685.

Ptak R, Schnider A. 2011. The attention network of the human brain: relating structural damage associated with spatial neg- lect to functional imaging correlates of spatial attention. Neuropsychologia. 49(11):3063–3070.

Shulman, G. L., Astafiev, S. V., McAvoy, M. P., d'Avossa, G., & Corbetta, M. (2007). Right TPJ deactivation during visual search: functional significance and support for a filter hypothesis. Cerebral Cortex, 17(11), 2625-2633.

Vandenberghe R, Molenberghs P, Gillebert CR. 2012. Spatial atten- tion deficits in humans: the critical role of superior compared to inferior parietal lesions. Neuropsychologia. 50(6):1092–1103.

Reviewer #3 (Remarks to the Author):

Sani et al sought to identify a ventral/temporal node to the human endogenous attention network, which is typically characterized as consisting of primarily parietal and frontal regions. This work is motivated by several recent studies identifying a similar region in macaque monkeys (most by the authors), PITd. The authors conducted a human fMRI study in which participants endogenously attended to a rapidly-changing moving dot pattern on either the left or right side of fixation and made a saccadic response to one of 8 target stimuli when they detected an extended motion pulse (an identical task to that used to identify this region in macaques). They performed some standard block-design analyses, along with some classic localizers, to identify a cluster of voxels in the ventral temporal cortex, overlapping with a previously-defined region phPIT (Kolster et al 2010), that responds more strongly when attending contralateral than ipsilateral, and does not have strong sensory responses during face- or motion-localizer tasks, mirroring results from macaques (Stemmann & Freiwald, 2016). They went on to use the large-scale diffusion imaging dataset acquired by the human connectome project to establish fiber tracts connecting each of the 3 nodes (phPIT, FEF, and what they call LIP). Altogether, the evidence seems strong that there is indeed a region of the temporal cortex that is active when participants attend contralaterally, and that this region seems to be connected to other regions thought to comprise the human attentional control network, in line with recent macaque studies.
First, I have to declare that I have little to no background in diffusion imaging methods/analysis, so my review is based solely on the functional imaging aspects of the manuscript. That said, I think the authors are making a compelling case, and the data seems to support their conclusions. A new ventral node of the human attention network is a novel discovery, and will likely be of broad interest to the readership of Nature Communications. However, for the manuscript to be suitable for publication, it needs to substantially expand the functional imaging results and methods sections (at present, I don't think I could reproduce this result based on the manuscript alone), and the authors should clarify the basis for their conclusions that phPIT is a 'control' region. One of my comments below may require additional data – I understand that in the present climate data acquisition might be impossible, so I defer to the judgment of the editor(s) as to whether such data is truly necessary to address my concerns.
Major comments:
1. The report does a nice job of illustrating a focal patch of activation during endogenous contralateral attention in phPIT, but I'd like to see a more thorough examination of the human neuroimaging data, in line with what was reported in Stemmann & Freiwald, 2016. Specifically, can the authors show: (a) something like a bar graph of attend contra vs attend ipsi for a similar set of ROIs (V1-hV4, V3AB, MT+/MST, IPS0-3, etc), (b) activation in each ROI during each localizer, and (c) AMIs for each ROI (can be easily derived from (a)). Basically – is there evidence that this region is really unique among the other regions that will also likely show evidence of attentional modulation? This can only be shown via direct comparison of response properties across regions, as the authors showed previously.
2. At several points in the discussion, the authors describe phPIT as an attentional 'control' region. I'm not sure this conclusion is justified based on what seems to be two lines of evidence: stronger activation when attend contra > attend ipsi, and lack of obvious response to particular localizers (but see comment 1). For example, across many previous reports V1 also shows strong attentional modulation, and does not necessarily have strong preference to one or another type of visual stimulus. What basis, in their own data, do the authors have for inferring a 'control' function of this region, rather than showing that it is attentionally modulated, like much of extrastriate visual cortex in humans? I understand the link to macaque studies (especially Boghadi et al 2019 and Stemmann & Freiwald 2019), but this study itself seems to just be showing attentional modulation of this region, rather than a control role. I'll mention that I'm quite convinced by the authors'

arguments throughout that this region is a much more likely homolog to macaque PITd than human TPJ, though I'm still not sure I'm convinced there's a direct 'control' role demonstrated in the present study. The authors may also wish to consider other recent studies investigating the extent to which parietal and frontal 'control' regions may also carry representations of attended information (e.g., Ester et al, 2016).

3. Boghadi et al 2018 & 2019 describe a similar-seeming area, which they call FST/IPa, in macaques. The authors cite these studies as describing PITd, though the authors of the original studies seem to make an effort to focus on their particular region/nomenclature. Can the authors clearly state how the FST/IPa regions examined in these previous studies relate to the PITd region discussed/studied by the authors?

4. The authors cite a separate report (Kolster et al, 2010) describing the retinotopic organization of area phPIT with respect to other retinotopic regions in the human visual system. However, there is a tremendous amount of variability between participants (this can be seen in the location of phPIT in the supplemental figures of the present study as well) – so ideally, the authors would be able to present the attentional task activations alongside at least a few individual participants' retinotopic maps. Given the current public health crisis, I imagine acquiring new human imaging data is impossible. I think this remains an important point to address, but will leave it to the editor(s) to determine whether this concern is justified.

Minor comments:

5. I think the authors can/should more clearly delineate which aspects of the report come from their sample of participants (I understand the functional MRI data do), and which come from the HCP dataset. How are these interrelated? There are hints throughout the methods but I'm still not entirely sure I understand. Is there any reason they did not acquire diffusion data on this sample?

6. Functional data – alignment across subjects: for figures in which data is aggregated across subj (Figs 3 & 4), how is the data aligned? It seems as though analyses were conducted in SPM8 standard space after volumetric smoothing (implied on line 513). Lately it seems like aggregating data across subjects for visual system mapping is more effective using surface-based coregistration (e.g., using Freesurfer to warp surfaces together rather than volumes). I wonder if the authors have tried this? Indeed, for the single subject analyses (Supp Figs), it appears the analyses were performed/displayed in each subject's native surface space. More clarity here and throughout would help the reader understand the analyses performed.

7. Fig. 3B: did the authors conduct 2 GLMs, one for each hemisphere? (e.g., for the LH, contrast attend right > attend left; for RH, attend left > attend right)? The different color bars for each hemisphere suggest this is the case. I'll also note that the title of this panel (ATTEND LEFT > ATTEND RIGHT) must be incorrect, or must only apply to the right side. Otherwise, we'd expect to see one hemisphere in cool colors and the other in warm colors.

8. fMRI data presentation throughout is a bit confusing. In the methods (line 515), the authors mention using $p < 0.001$ FWE to define significant voxels. But it seems like different thresholds are used throughout – especially Fig. 3B and Fig. 4A/B. Moreover, I don't understand what's presented on the colorbars – T-values? Why isn't the full range of the colorbars used (e.g., no dark orange in Fig 3B)? And the colorbar in Fig. 4B doesn't show a lower bound for each direction.

9. fMRI methods – the authors refer to their previous publication (Stremmann & Freiwald, 2016), but this is a single-subject fixed-effects report – typically random effects studies employ somewhat different procedures. The authors should add sufficient detail to understand how data was aggregated (& aligned, see above) across participants, and how statistics were performed.

10. Task – I'm a bit confused by the task design and stimuli. During the 'passive fixation' intervals described by the authors, is there still a RDK stimulus on the screen? (suggested by legend in Fig. 3A; Fig. 2B) Or, is there a blank fixation screen (as suggested by the first panel of Fig. 2A)? Moreover, when is the eye movement required to be made? During the stimulus presentation sequence after a target is detected? Perhaps the authors could show an example trial's eye trace measured during scanning beneath the event timing graph in Fig. 2B.

11. Eyetracking – there are essentially no details provided for how eyes were tracked inside the scanner – this should be substantially elaborated, especially because it appears that the task timing was contingent upon correct behavioral performance (wait for 7 'hits' to move on to next block).

12. Additional methods details: the authors should provide further details about (a) functional localizer tasks (stimulus size, duration, block properties, task, etc) – there's only 5 lines to account for at least 2 tasks now. Additionally, the reporting about how many runs are conducted is somewhat confusing – were 3 runs of the main task acquired per scanning session? Or across both

sessions?
13. In Supplementary Fig. 2, I wonder why the authors see no object-selective voxels? I understand this is an example participant, but is this result common?
14. The three-node structure of the attention network the authors describe reminds me of a talk I recently saw by Randy Buckner, in which he described a 3-node (temporal/parietal/frontal) motif for resting state networks in humans and macaques. I haven't read it yet, but I believe this is all summarized in Buckner & DiNicola, 2019, Nature Reviews Neuroscience. I only mention this in case the authors might be interested in this framework – the similarity is somewhat striking though!

Reviewer #4 (Remarks to the Author):

Sani et al: The human attentional control network includes a ventro-temporal cortical node

This is an interesting and ambitious study that present a novel idea. The authors' laboratory have previously argued that a region in the dorsal part of the posterior inferotemporal (PITd) cortex, in the ventral bank of the posterior superior temporal sulcus, with a role in exerting attentional control. This was an important observation because for many years it has been widely believed that the cortical centres exerting attentional control are in the parietal and frontal cortex. The authors build on their prior work in macaques and test the applicability of these ideas to the understanding of the human brain. Using the same cognitive task they identify a posterior temporal cortical region in the human brain with a similar activation profile. Second, they show that the topological relationship between that area and adjacent areas concerned with face processing and motion processing is similar in humans and macaques. Finally they show that the connectional anatomy of the human region, as inferred from diffusion weighted imaging, is similar to the connectional anatomy of the macaque PITd region.

The study is convincing as it currently stands. The following minor points might be considered in a revised version of the manuscript.

1 An argument is made that putative human PIT (phPITd) has activity that increases with attentional demand but not with visual motion per se. Would it be useful to show the visual motion onset related activity in the passive viewing condition in the phPITd region? This might be done by extracting effects timelocked to motion onset versus some other trial time point from a region of interest in the phPIT.

2 Diffusion weighted imaging is used to estimate connectivity between the phPIT and the lateral intraparietal area (LIP) in posterior parietal cortex and frontal eye field (FEF). The authors conclude that like macaque PIT, the phPIT has a high probability of connectivity with LIP and FEF. Would it be useful to supplement this demonstration with evidence of connections that both phPIT and macaque PITd lack? For example, while PITd in macaque is connected with LIP and FEF are there areas that adjacent brain regions are connected to but to which PITd is not connected? For example, does PITd have the same connections with extrastriate visual areas as MT+? If not, then is the same true of human phPIT? In other words, does phPIT have both the connections that resemble macaque PITd and does it lack the connections that macaque PITd lacks compared to its neighbours? An approach analogous to this one – examining both connections that ae present and connections that are absent – was used by Mars and colleagues (PNAS, 2013) when identifying a homologue of human TPJ in the macaque on the basis of MRI-derived estimates of connectivity.

3 Figure 7. I realise that this figure is diagrammatic and that every last details is not to be over interpreted. Nevertheless, human FEF is shown in two different locations in the panels on the left and right of figure 7. Is that intentional? On the left FEF is shown in a ventral location but on the right it is in a dorsal location. On the left it is the caudal bank of the precentral sulcus. On the right it is in the fundus of the precentral sulcus. The figure suggests that human precentral sulcus is one very big sulcus that spans the dorsoventral extent of the frontal lobe but, as I am sure the authors know, the human superior and inferior precentral sulci are usually separate sulci that do not join (Germann et al., J. Comp. Neurol., 2005).

RESPONSE TO REVIEWERS

We thank the Editor and all the reviewers for their careful evaluation and highly constructive suggestions on our manuscript. All reviewers were very positive about our work, and we would like to thank them for that and for their suggestions. We made a substantial revision and were able to address their concerns and questions. We are convinced the manuscript has greatly improved, thanks to all the suggestions we received.

Below, we describe our point-by-point responses to the comments. All changes to the text of the manuscript have been marked in yellow to help the review process.

Reviewer #1 (Remarks to the Author):

The functional characterisation of human brain areas is making slow signs of progress using the combination of direct electrical recordings in non-human primates and advanced neuroimaging in humans.
Within that context, Sani et al. used multimodal imaging to localise in humans an attentional area well known in non-human primates, the dorsal portion of posterior inferotemporal cortex (PITd).
To do so, Sani et al. used the same attentional paradigm used in macaques and n= 10 humans to define homologies and further used white matter connectivity to confirm homologies in term of connectivity.
Overall I think this is a valuable paper, which, after substantial revision, will become a reference in the field of attention, neuroimaging, clinical neuroanatomy and comparative anatomy.

We thank the Reviewer for this very positive characterization of our work and for the constructive comments on our manuscript.

General comment.
The use of the term "attention control" as a function, is confusing. Decades of work on attention dissociated attention into spatial attention, feature-specific attention, endogenous, exogenous, preparative. The authors need to clarify exactly the functions they intend to map onto the brain.

We thank the Reviewer for suggesting a potential source of confusion in the terminology. We clarify early on in the title, in the abstract, in the introduction and in the schematic introductory figure that we are investigating endogenous attention. We more extensively

explain in the method section that we used a motion discrimination task for the study of sustained endogenous spatial attention

Changes to the text: *page 1-3 (title, abstract, introduction) and page 29 (method section) of the revised manuscript*.

The use of 10 participants is really small as a sample compared to other studies and should be explained/acknowledged.

We thank the reviewer for pointing to the need for clarification. In the current study, we followed the approach by Kolster and colleagues (2010) who localized phPIT successfully and consistently across subjects. In their study they enrolled 11 right-handed healthy human volunteers. Despite using a small sample compared to other studies, participants were scanned with several tasks and localizers. This was critical for their study, because of the small size of phPIT and because of the variability of its location across different subjects. Likewise, here we scanned 10 participants (12 in the revised version). Each participant was scanned in three sessions (days): for two sessions subjects were scanned during the performance of the attention task for two runs per day (approximately 18 min measurement each run); then subjects performed two additional localizers (motion, face-objects-place localizer; one run per subject per each localizer). To further strengthen the within subject localization of PIT, in the reviewed version, we also added the analysis of a retinotopy and an eccentricity experiment. In sum, to achieve the goals of our study, it was critical not so much to have a large sample size, but rather to define for each subject the spatial location of attention-modulated areas relative to feature-selectivity maps. We now clarify this approach in the manuscript.

Furthermore, an intensive training-scanning protocol had to be implemented, and its feasibility relied on a smaller sample size: because of the comparative nature of the current study, before the fMRI sessions, all subjects have been trained on performing the attention task. They were trained on 5 separate days and performed 6 sessions of 120 trials each day. This was done to minimize effects of learning in the data and better compare the current dataset with that in Stemmann and Freiwald 2016, where non-human primates had been also extensively trained before the fMRI sessions.

We would like to point out that other studies have successfully used similar sample size and approach for comparative imaging of the temporal lobe (e.g. Lafer-Sousa, Conway, and Kanwisher J.Neurosci. 2016), as well as for more standard investigations of the attentive function where the sample size was as low as 4 (Pestilli et al., Neuron 2011; Liu, Pestilli, and Carrasco Neuron 2005).

Change to the text: In response to the reviewer comment, we now better explain this anatomically informed approach in the Methods section and highlight the number of runs

across scans. We have also added and new figure with the retinotopy/eccentricity experiments (Supplementary Figure 3) to show that we have good quality of results within each subject.

Pages 29 of the revised manuscript

Methods comments
Part of the argument of the authors is to demonstrate that the region reported is attention specific. Being attention specific (not tested in the study) is different from being motion and shape independent (actually tested in the study). Why doesn't another attention paradigm not using motion activate the dorsal portion of posterior inferotemporal cortex? Would it be possible that the dorsal part of the posterior inferotemporal cortex would be specific to the attention to motion? Besides recent work published by Chen, Wasserman et al. Nature Communications 2020 "The visual word form area (VWFA) is part of both language and attention circuitry" stress that a region can be located at the interplay between two systems. Hence, the mapping of function to brain regions is not systematically a 1 to 1. I struggled with this point in the manuscript.

We agree with the reviewer and thank her/him for offering us an opportunity to better explain this concept. The current study demonstrates that phPIT is activated by attention and yet is not activated by specific categories of stimuli, like faces, scenes, or motion –here the property to be discriminated during the attention task. This suggests that (1) differently from early visual areas, PIT attentional signals shown here are not a modulations of the task-relevant dimension, but more likely general attention control signals, (2) PIT may subserve other visual dimensions and categories, because it is not tuned to any particular one. This in turn suggests that another attention task would as well activate PIT. This prediction is supported by two recent independent studies in the non-human primate (Stemmann and Freiwald, 2016; Bogadhi et al., 2018). In these fMRI studies, PIT was activated by multiple attention tasks, not only involving motion but also color and orientation. Consistently, PIT single neurons show strong attentional signals across attentive motion and color discrimination tasks (Stemmann and Freiwald 2019).

We, in sum, believe that PIT is not solely involved in attentive motion processing, but rather plays a more general role in attention, and possibly in other cognitive functions. Its vicinity to specialized areas like the VWFA (whose center is less than 20 mm away from PIT) strongly suggests PIT is in the perfect location to get access to the most relevant visual information needed for behavior and that it is located at the interplay between multiple systems. The connectivity with dorsal attention areas shown in Chen, Wasserman et al. Nature Communications 2020, might indeed be shared with or even intercept that of PIT.

Change to the text: We now better address this point in the discussion, where we take an opportunity to draw a link between the current study and recent evidence from Chen et al., 2019

Page. 23-24 of the revised manuscript

Discussion/interpretation comments.
"The control of endogenous attention has traditionally been attributed, with much support, to a fronto-parietal network (Corbetta et al., 2008; Gottlieb et al., 1998; Kastner and Ungerleider, 2000; Thompson and Bichot, 2005). Our results suggest that there is a novel area of attention control, located in a specific part of the temporal lobe, phPIT, and that this area forms a node in the endogenous attention control network"
This is inaccurate. The areas reported by the authors has been discussed by many authors including Corbetta and Shulman and rejected based on information derived from electrophysiology "Areas in the occipital lobe (fusiform and MT+) respond transiently to the cue, whereas areas in the dorsal posterior parietal cortex along the intraparietal sulcus (IPs) and in the frontal cortex (at or near the putative human homologue of the FRONTAL EYE FIELD, FEF) show a more sustained response." (Corbetta and Shulman 2002). Further, an area matching phPIT has been reported in Corbetta et al. Nature Neuroscience (2005).

We thank the reviewer for pointing out this imprecision. We now clarify in the discussion (see *changes to the text at page 23 of the revised manuscript*) that previous studies have discussed the possible role in attention of an area located anterior to, and possibly compatible with, phPIT (referred as fusiform). More specifically, fusiform along with other regions, has been shown to have predictive signals that reflect the use of the cued information, thus supporting the hypothesis that this activity may represent maintenance of attention at the cued location (Sapir et al., 2005). Despite this circumstantial evidence, PIT role in attentional control has never been stated explicitly and has remained largely uncharacterized. In this context, we believe our work represents a key step forward in that it connects and definitively validates earlier observations. Critically, while previous fMRI studies showed less sustained attentional signals in MT+ and Fusiform (Corbetta et al., 2000), recent fMRI-guided electrophysiological recordings points to extremely strong and sustained responses in macaque PIT (> 4s; Stemmann and Freiwald, 2019). Current evidence from our lab further strengthen this idea by showing that PITd sustained response happens, both in presence and absence of visual stimulation [Redacted]. This evidence is instrumental to corroborate the role of PIT in attentional control.

Redacted

"A previously uncharacterized vertical pathway connects phPIT and dorsal attention area LIP". There is already a confusion between the anterior portion of the vertical occipital fasciculus and the posterior portion of the posterior segment of the arcuate fasciculus. I would suggest the authors to describe this connection as a sub-portion of the posterior segment of the arcuate fasciculus. We want to avoid an unnecessary complexification of neuroanatomy.

We thank the reviewer for the thoughtful comment. Indeed, there is ongoing research on the definition and clarification of the posterior vertical tracts in the human brain. We note that in addition to the pArc and VOF, other tracts connecting the dorsal and ventral posterior human cortex have been described. These tracts comprise of the TP-SPL, MdLF-SPL, MDLF-Ang, TP (see for example Bullock et al., 2019) and sFOF (Forkel et al. 2014) as suggested by the reviewer in the comment below. Our group has been working to clarify the literature in regard to all these tracts. The current tendency of clustering multiple tracts into either the VOF (which by definition should be limited to within the Occipital lobe) or the pArc (which has terminations biased toward the lateral portion of the dorsal and temporal cortex only) might need to be revised. Indeed, cortical function can be subdivided into multiple functional and anatomical regions in the posterior dorsal and lateral cortex. We believe the white matter might also follow a similar finer grain subdivision.

In addition to this need for a finer-grained subdivision of the tracts, we note that the tract at stake has clearly distinct properties from the previously reported ones:

(1) Tract location is only partially overlapping with other tracts (mostly the pArc and TP; Figure 7A). Indeed, the tract of interest fills in a portion of the white matter not currently assigned to any of the other tracts. Below we reproduce figure 2b from Bullock et al., BSAF 2019. We have added in yellow (dashed line) the trajectory of the tract we report to evidence that it is close by but different than the previously reported tracts.

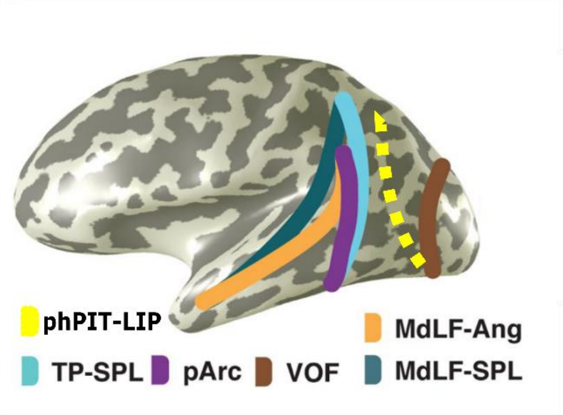

(2) The tract trajectory is differently oriented than the other tracts. As the evidence in Supplementary Figures 6 implies, the tract of interest runs vertical and oblique from lower-posterior to superior-anterior. This is a different orientation than previously reported tracts that run anterior-inferior to posterior-superior.

<u>Changes to the text:</u> In response to the reviewer comment and to avoid complexification, we state that given the percentage of overlap with the pArc our tract likely constitutes an unreported brunch of the pArc - *page 19 and related figures of the revised manuscript*.

For completeness, we have also updated the Discussion section describing the relation to other tracts. We added a sentence in the discussion describing the tract of interest precisely how the reviewer suggests: “the phPIT-LIP tract travels partially via a sub-portion of the posterior segment of the arcuate fasciculus and partially via a sub-portion of the TP-SPL” - *Page 26 of the revised manuscript*.

Concerning the discussion of the anatomy, the description of the occipital projections of the second branch of the superior longitudinal fasciculus / superior fronto-occipital faciculus described by Forkel et al. "The anatomy of fronto-occipital connections from early blunt dissections to contemporary tractography" in Cortex 2014 might be of interest for the interpretation of the findings.

We thank the reviewer for suggesting the intriguing hypothesis that the phPIT-FEF connection described here might also be compatible with the superior fronto-occipital fasciculus (sFOF; Forkel et al., 2014). sFOF runs between occipital and frontal lobes, lateral and dorsal to the corpus callosum, and likely represents a longer branch of the SLF system (Forkel et al., 2014). The presence of this tract has been hard to consistently define in healthy human subjects because of its location in an area with high density of crossing fibers (Forkel et al., 2014). This is reflected in the properties of the connection defined here, which is on average weaker than the others (Figure 6E) and shows in some subjects some branches (Figure S5).

Changes to the text: In response to the reviewer's comment to this and the previous point we added a paragraph in the Discussion relating current results to existing tracts and nomenclature. We now include sFOF as a possible pathway for phPIT-FEF connection *Page 26 of the revised manuscript*.

Additional analyses suggestion

Since the authors found a homology between humans and macaque, it would be interesting to make some comparisons of the connectome of the phPIT between the two species.

Redacted

Redacted

Reviewer #2 (Remarks to the Author):

In this ms the authors report the presence of a new attentional control node in the human inferotemporal cortex. Although a functionally and anatomically similar area has been recently described in the monkey brain, this study provides the first fMRI evidence in humans. This inferotemporal area is likely to be involved in representing space in object-centered / allocentric coordinates. The study provides a fine picture of the anatomical localization of this new area and of its anatomical connections with the other major parietal and frontal nodes of the attentional hub. I have no major technical or methodological objections. In what follows I have listed a number of minor point-to-point suggestions and a request for an additional analysis of fMRI data.

We thank the reviewer for appreciating the conceptual, as well as the technical and methodological value of the current work. We addressed all the reviewer's concerns. As a result, the current work has now acquired a stronger connection with previous literature.

Minor points.

Abstract, page 2: some parts of the text could be toned down a bit. Line 19: "Here we challenge this notion .." , could be changed in " Here we expand this notion..".
I would suggest to shorten and separate in two parts the third sentence like this:
"By combining a demanding behavioral paradigm with functional neuroimaging and diffusion tractography, we show that like fronto-parietal attentional areas, the human posterior inferotemporal cortex exhibits significant attentional modulatory activity. This area is functionally distinct from surrounding inferotemporal areas and is directly connected to parietal and frontal attentional regions.

We thank the reviewer for this suggestion and modified the abstract accordingly.
- page 2 of the revised manuscript -

Page 4 line 60 – 61 : The role of TPJ in contextual updating was initially suggested in Doricchi et al. (2010; see also Macaluso and Doricchi, 2013), who provided evidence for the different roles of that the left and right TPJ play in this process: the left TPJ is activated by valid targets that "match" the position pointed by the cue, the right TPJ by invalid targets that "mismatch" the position that was initially pointed by the cue.

Page 4 line 68 – 69 "...human TPJ activity is actually reduced during endogenous attention." : no reference is provided here. The two fMRI studies that showed reduction of TPJ during orienting of endogenous attention are Shulman et al. (2007) and Doricchi et al. (2010). The first study shows that in a RSVP task, the more the moment of target occurrence approaches the more the right TPJ is deactivated. The second study shows that during orienting of spatial attention with endogenous cues, the more cue are predictive of target location the more TPJ is deactivated.

We thank the reviewer and added the original citations. We believe these additions made the introduction more complete and better connected to the previous literature.
- page 4 of the revised manuscript -

Lines 391-393: "Although similarly direct evidence is lacking in humans, neuropsychological evidence has increasingly pointed to a role of the temporal lobe in attentional control (Karnath et al., 2001)" : I would reccommend caution in reporting this statement because: a) the area initially highlighted by Karnath et al. (2001) was in the central sector of the Superior Temporal Gyrus: this area is far away from the phPIT and is anterior to the TPJ. Since the authirs have well functionally differentiated their phPIT area fromthe TPJ, I see really no reason to make an analogy between the phPIT and the central STG; b) ensuing anatomical investigations have systematically failed to replicate Karnath et al. findings.

We thank the reviewer for this suggestion. To support the idea that there has been interest in investigating the role of the temporal lobe in neglect syndrome we now point to two references that targeted an area very close to PIT: Aiello et al., 2012; Verdon et al., 2010.
- page 25 of the revised manuscript -

Line 395: The study by Doricchi and Tomaiuolo (2003) is the first study that pointed out the role of Superior Longitudinal Fasciculus disconnection in spatial neglect (not the SMGyrus). This original finding was confirmed two years later by the neurosurgical inactivation study

by Thiebaut de Schotten et al. (2005). This reference should be reported later on, on line 407 were the role of white matter disconnection is currently quoted in the paper.

We thank the reviewer and now properly acknowledge original citations.
- page 25 of the revised manuscript -

Line 207 : very minor typo " .. operated by (Glasser et al., 2016) " should be probably rewritten as " .. operated by Glasser et al. (2016) "

Line 213: other minor typo "(LiFE, (Caiafa and Pestilli, 2017; Pestilli et al., 2014)) " should be probably rewritten as "(LiFE: Caiafa and Pestilli, 2017; Pestilli et al., 2014) "

Following the reviewer suggestion, we corrected the typos in the reviewed version.

Lines 442-460: In these lines the authors give emphasis to the anatomical and functional differentiation between the TPJ and PITd. In doing this they define the TPJ a "core node" of the ventral exogenous network. Nonetheless, views on the role of the TPJ are rapidly changing. First, this area is also involved in endogneous orienting, as it gets da-activated during cued orienting (see above-mentioned references: Shulman et al., 2007, Doricchi et al., 2009. Second, there is growing evidence that the TPJ is involved in late phases target-processing linked to contextual updating (references: Doricchi et al., 2010; Geng and Vossel, 2013; Macaluso and Doricchi, 2013). Third, the dorsal-ventral dichotomy of attentional control might not fit with the role that the superior parietal lobule might play in exogenous re-orienting to invalidly cued targets (Ptak and Schnider 2011; Vandenberghe et al., 2012; Dragone et al., 2015). In this part of the discussion section, the authors might wish to offer a more articulated view of the functional role of the TPJ.

We thank the reviewer and added a section where we offer a more articulated view on the functional properties of TPJ. Following the reviewer suggestion, we describe TPJ roles in endogenous orienting and late phases target processing, as well as the role in exogenous re-orienting of the parietal lobule.
- page 27-28 of the revised manuscript -

Major point.

Page 10 lines 162-164: I wonder if finer grain results could be obtained by supplementing the "attend contralateral" vs "attend ipsilateral" contrast with "attend left" vs "attend right" contrasts run separately for trials that required the same directional leftward or rightward saccades. This type of contrast would allow, for example, to investigate whether the phPIT

areas in each hemisphere show preferential responses for one of the two hemispaces and, in each area, whether there is a preference for attending leftward vs rightward motion or, put in object-centered terms, toward the left or right side of the lateral clouds of dots. Could the authors please address this proposal and eventually enclose these supplementary analyses in their revision of the manuscript?

We thank the reviewer for this extremely interesting suggestion. We agree this would be an important conceptual point to be addressed. However, the goal of the current study was to establish the role of phPIT in attentional control and, despite the importance of the dichotomy between space-centered vs. object-centered representation, it falls beyond the current scope. The experimental approach was indeed specifically designed to achieve this goal and, consequently, does not allow us to test the suggested prediction with sufficient statistical power and without incurring confounds.

Changes to the text: Even if we were unable to test the proposed hypothesis, following the reviewer suggestion, we included a section in the Discussion to put forward this interesting conceptual point.
- page 24 of the revised manuscript -

In what follow we provide a detailed explanation as to why we were unable to test whether there is a preference for attending leftward vs rightward motion in area phPIT.

The contrast attending leftwards vs. rightwards motion will results in 4 trials per each subject per session on average because 8 different directions of motion (leftwards, up-left oblique, upwards, up-right-oblique, rightwards, down-right-oblique, downwards, down-left-oblique) have been used. This would lead to a low statistical power. Because of the scarcity of trials the unattended stimulus will not have all the possible directions of motion which, in turn, will also result in an unbalanced contrast.

More importantly, we will be unable to disentangle attend rightward vs. leftwards motion from saccadic eye movements to the right target vs left target. Subjects were asked to make a saccade towards the target matching the direction of motion of stimulus in the attended hemifield. Therefore, attend rightward motion is always associated with a saccade to the right, while attend leftwards motion is always associated with a saccade to the left. In our main contrast (attend right vs. left) saccades were made equally frequently in 8 direction and saccadic eye movement were therefore balanced in the two attentional conditions.

Our final concern is on the timing of visual stimulation. In each trial two random dot stimuli were always presented on the left and right hemifield. Each stimulus rapidly, continuously and independently changed direction of motion every 60ms (Figure 2A). At a random time, each stimulus independently ceased changing for 500ms. This design strongly encourages

the subjects to keep sustained attention focused on one hemifield during the rapid visual presentation to catch the behaviorally-relevant event. However, it asks the subjects to keep attention to leftward (or rightward) motion for only 500 ms, which for fMRI dynamics is an extremely short period of time.

Future experiments will need to be designed to properly test the prediction that phPIT activity is related to object-centered spatial processing.

References.

de Schotten, M. T., Urbanski, M., Duffau, H., Volle, E., Lévy, R., Dubois, B., & Bartolomeo, P. (2005). Direct evidence for a parietal-frontal pathway subserving spatial awareness in humans. Science, 309(5744), 2226-2228.

Doricchi, F., Macci, E., Silvetti, M., & Macaluso, E. (2010). Neural correlates of the spatial and expectancy components of endogenous and stimulus-driven orienting of attention in the Posner task. Cerebral Cortex, 20(7), 1574-1585.

Dragone A, Lasaponara S, Silvetti M, Macaluso E, Doricchi F. 2015.
Selective reorienting response of the left hemisphere to in- valid visual targets in the right side of space: relevance for the spatial neglect syndrome. Cortex. 65:31–35.

Macaluso, E., & Doricchi, F. (2013). Attention and predictions: control of spatial attention beyond the endogenous-exogenous dichotomy. Frontiers in human neuroscience, 7, 685.

Ptak R, Schnider A. 2011. The attention network of the human brain: relating structural damage associated with spatial neg- lect to functional imaging correlates of spatial attention. Neuropsychologia. 49(11):3063–3070.

Shulman, G. L., Astafiev, S. V., McAvoy, M. P., d'Avossa, G., & Corbetta, M. (2007). Right TPJ deactivation during visual search: functional significance and support for a filter hypothesis. Cerebral Cortex, 17(11), 2625-2633.

Vandenberghe R, Molenberghs P, Gillebert CR. 2012. Spatial atten- tion deficits in humans: the critical role of superior compared to inferior parietal lesions. Neuropsychologia. 50(6):1092–1103.

Reviewer #3 (Remarks to the Author):

Sani et al sought to identify a ventral/temporal node to the human endogenous attention network, which is typically characterized as consisting of primarily parietal and frontal regions. This work is motivated by several recent studies identifying a similar region in macaque monkeys (most by the authors), PITd. The authors conducted a human fMRI study in which participants endogenously attended to a rapidly-changing moving dot pattern on either the left or right side of fixation and made a saccadic response to one of 8 target stimuli when they detected an extended motion pulse (an identical task to that used to identify this region in macaques). They performed some standard block-design analyses, along with some classic localizers, to identify a cluster of voxels in the ventral temporal cortex, overlapping with a previously-defined region phPIT (Kolster et al 2010), that responds more strongly when attending contralateral than ipsilateral, and does not have strong sensory responses during face- or motion-localizer tasks, mirroring results from macaques (Stemmann & Freiwald, 2016). They went on to use the large-scale diffusion imaging dataset acquired by the human connectome project to establish fiber tracts connecting each of the 3 nodes (phPIT, FEF, and what they call LIP). Altogether, the evidence seems strong that there is indeed a region of the temporal cortex that is active when participants attend contralaterally, and that this region seems to be connected to other regions thought to comprise the human attentional control network, in line with recent macaque studies.
First, I have to declare that I have little to no background in diffusion imaging methods/analysis, so my review is based solely on the functional imaging aspects of the manuscript. That said, I think the authors are making a compelling case, and the data seems to support their conclusions. A new ventral node of the human attention network is a novel discovery, and will likely be of broad interest to the readership of Nature Communications. However, for the manuscript to be suitable for publication, it needs to substantially expand the functional imaging results and methods sections (at present, I don't think I could reproduce this result based on the manuscript alone), and the authors should clarify the basis for their conclusions that phPIT is a 'control' region. One of my comments below may require additional data – I understand that in the present climate data acquisition might be impossible, so I defer to the judgment of the editor(s) as to whether such data is truly necessary to address my concerns.

We thank the reviewer for pointing to the importance of this discovery and the strengths of our work. Following her/his suggestions we expanded the functional imaging results and methods. We believe this substantially improved the quality of the current research and of the current conclusions. It also made the study more reproducible.

Major comments:
1. The report does a nice job of illustrating a focal patch of activation during endogenous contralateral attention in phPIT, but I'd like to see a more thorough examination of the human neuroimaging data, in line with what was reported in Stemmann & Freiwald, 2016. Specifically, can the authors show: (a) something like a bar graph of attend contra vs attend ipsi for a similar set of ROIs (V1-hV4, V3AB, MT+/MST, IPS0-3, etc), (b) activation in each ROI during each localizer, and (c) AMIs for each ROI (can be easily derived from (a)). Basically – is there evidence that this region is really unique among the other regions that will also likely show evidence of attentional modulation? This can only be shown via direct comparison of response properties across regions, as the authors showed previously.

Following the reviewer suggestion, we implemented a region of interest (ROI)-based analyses to further test our hypotheses. We defined ROI using the Glasser atlas, guided by the eccentricity and retinotopy mapping (for details see response to comment #4 below and Methods section). We quantified activity in each ROI to determine the profile of response of each region to the attention task, the motion localizer, and the face-scene-object localizer, thus providing a richer profile of the activity of the ROIs (Fig. 5A-C, reproduced below). The Attention Modulation Index was not informative with the current dataset: the presence negative values can often led to index saturation. Therefore, to characterize the modulation and selectivity profiles of each ROIs we compared the response difference with response magnitude (Fig. 5D).

Area PIT shows a strong attention effect. PIT, in fact, is the area with the largest attention index among areas with a robust positive response to visual stimuli (Fig. 5D, left panel) and the largest response difference of all areas for attended versus non-attended stimulus (Fig. 5D, left panel). Attention effect in PIT are thus stronger than in early visual areas or in motion areas (Fig. 5A).

Motion selectivity, exhibits almost the reverse pattern of effects. As the motion localizer confirmed, early visual areas, traditional motion areas MT, MST, FST, and even LO1,2, and 3 exhibit strong preferences for motion over static stimuli (panel B). Critically, traditional motion areas are characterized by a strong response to moving stimuli and a non-significant response to static stimuli (panel B). PIT exhibits a small modulation by motion (Fig. 5B), which is smaller than that of the other areas relative to its visual activation (Fig. 5D), yet a strong activation by static stimuli.

Finally, the shape localizer, confirmed specialized processing for faces in FFC, for places in PHA1-2-3, for objects in LO1 and 3. PIT, among the most strongly activated areas, showed similar responses for faces, scenes, and objects (panel C).

Overall, PIT stood out as a highly visually responsive area. PIT responding strongly to both shape-less motion stimuli and motion-less shape stimuli, suggesting that neurons in this area are not strongly tuned and thus capable of representing any stimulus. PIT also stood out as an area whose activity is very strongly attention modulated, both in absolute terms and relative to its degree of visual activation (panel D). Importantly, PIT's response profile across all three tasks, strongly differs from that of nearby motion areas (in yellow) as well as from that of nearby temporal areas (in pink).

Following the reviewer suggestion, we integrated this new analysis in the revised manuscript as a main figure.

Changes to the text: page 33 (methods section) and page 11-14 (results section) of the revised manuscript

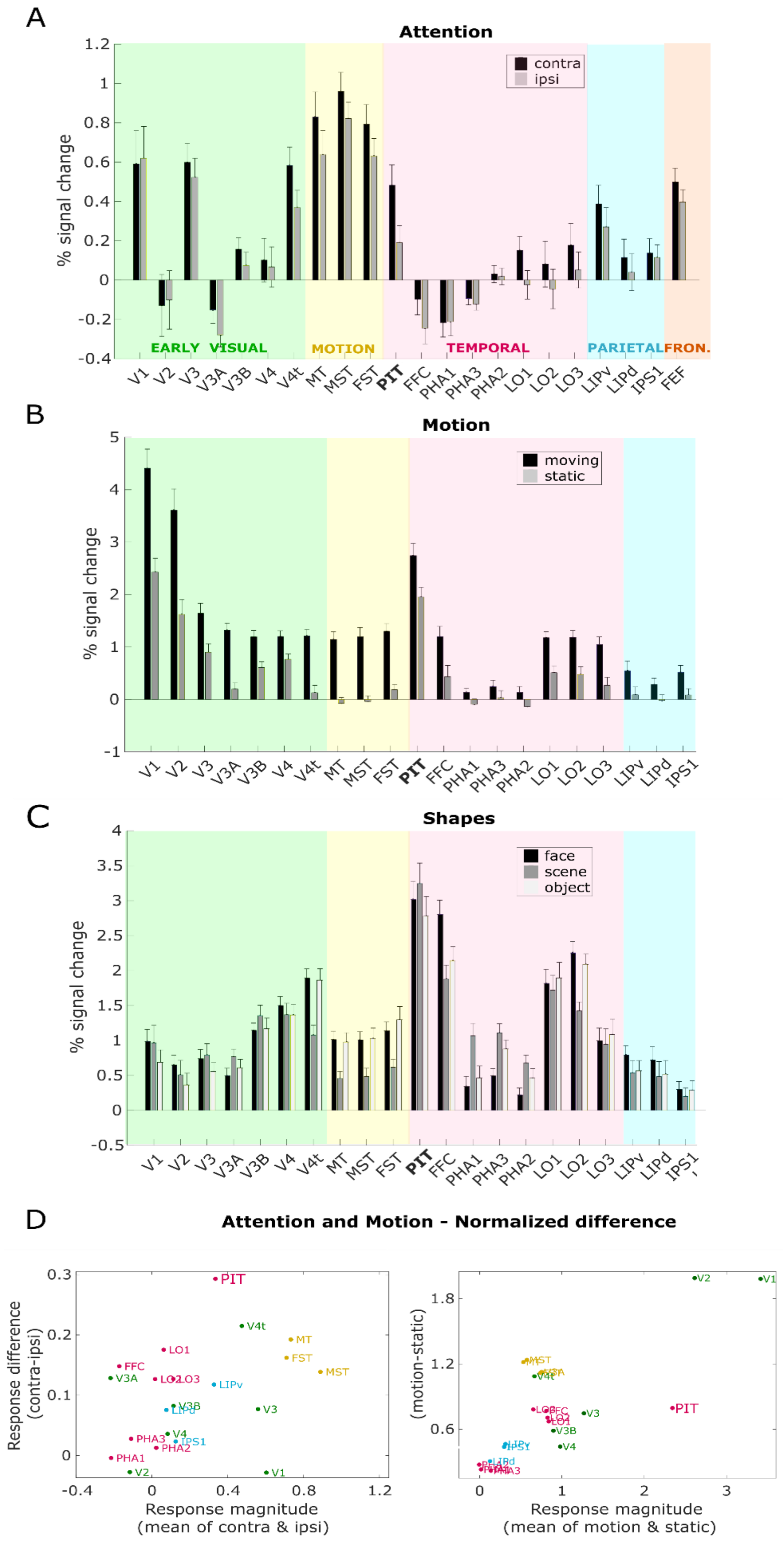

Figure 5. Comparative functional profile of cortical ROIs in the occipital, temporal, parietal, and frontal lobes. ***A.*** *Attentional modulation; the bar plot shows the average percentage signal change across subjects for each ROI (error bars indicate across-subject standard error). Signal extracted during the attend contralateral (Attend contra) and the attend ipsilateral (Attend ipsi) condition are shown in black and gray respectively;* **B**. ROI responses to motion and static stimuli; *bar plots show the average percentage signal change across subjects for each ROI in response to moving and static stimuli; same conventions as in A.* **C.** ROI responses to three shape categories; *bar plots show the average percentage signal change across subjects for each ROI in response to faces-scenes-objects; same conventions as in A.* ***D.*** *Attention and motion modulation profiles of ROIs relative to mean activation. Scatter plots show activation differences (vertical axis) as a function of average activation (horizontal axis) during the attention task (left) and the motion localizer (right). The ratio of response difference and response magnitude defines the attention index.*

2. At several points in the discussion, the authors describe phPIT as an attentional 'control' region. I'm not sure this conclusion is justified based on what seems to be two lines of evidence: stronger activation when attend contra > attend ipsi, and lack of obvious response to particular localizers (but see comment 1). For example, across many previous reports V1 also shows strong attentional modulation, and does not necessarily have strong preference to one or another type of visual stimulus. What basis, in their own data, do the authors have for inferring a 'control' function of this region, rather than showing that it is attentionally modulated, like much of extrastriate visual cortex in humans? I understand the link to macaque studies (especially Boghadi et al 2019 and Stemmann & Freiwald 2019), but this study itself seems to just be showing attentional modulation of this region, rather than a control role. I'll mention that I'm quite convinced by the authors' arguments throughout that this region is a much more likely homolog to macaque PITd than human TPJ, though I'm still not sure I'm convinced there's a direct 'control' role demonstrated in the present study. The authors may also wish to consider other recent studies investigating the extent to which parietal and frontal 'control' regions may also carry representations of attended information (e.g., Ester et al, 2016).

We thank the reviewer for suggesting caution when talking about attentional control. Demonstrating a control role for an attention area is indeed a very difficult argument to make based on human unimodal data alone and it has been a matter of intense debate in the field. We now clarify in the discussion that in the current study, the time-resolved causal evidence from non-human primates is not only used as motivation, but is also key element for the interpretation of human data beyond what can be deduced from the human neuroimaging data alone. Based in this multi-modal, multi-species approach we are currently proposing a role in attentional control for human PIT. In this framework, future studies will be able to further strengthen this proposal by specifically testing additional key properties typically attributed to control areas for area PIT. To better explain this concept, we added a supplementary table showing which features have been used in previous studies to support the notion that a given area has attentional control properties. In the table, we compare PIT, LIP, FEF, and also TPJ and V1 and provide example references to highlight what we already know about PIT and what needs to be further investigated.

Following the reviewer suggestion, we also acknowledge that the divide between areas with source and target roles in attention might be less distinct than previously thought, when considering the area representational properties (Ester et al, 2016). Interestingly, Ester and colleagues show that several fronto-parietal regions, but also an area compatible with PIT location, encode continuous representation of sensory information. This suggests that even in this framework, PIT, LIP and FEF share important similarities.

Changes to the text: *page 22, page 23, and page 50 of the revised manuscript.*

CONTROL PROPERTY	Theoretical proposals (1)	FEF (2)	LIP (3)	PIT (4)	TPJ (5)	V1 (6)
Activation during prolonged endogenous attention	✓	✓	✓	✓	x	x
Independence of specific visual features	✓	✓	✓	✓	✓	x
Causal relationship with attentive behavior/state	✓	✓	✓	✓	n.a.	x
Sustained neuronal response for attention signals	✓	✓	✓	✓	n.a.	x
Neuropsychological evidence	n.a.	✓	✓	✓	✓	x

Supplementary Table 3. *A checklist of functional properties required to define an attention control area is shown for dorsal attention nodes FEF and LIP, for PIT, and for TPJ and V1 for comparison. Example references are provided below: (1) Fecteau and Munoz, 2006; (2) Fecteau and Munoz, 2006; Bichot et al., 2015; Rossi et al., 2007; Moore and Armstrong, 2003; Kastner and Ungerleider, 2000; Corbetta and Shulman, 2002; (2) Kastner and Ungerleider, 2000; Corbetta and Shulman, 2002; (4) Stemmann and Freiwald, 2016, 2019; Bogadhi et al., 2018; 2019; Kolster 2010; 2014; (5) Doricchi et al., 2010; Shulman et al., 2007 (6) Serences and Boynton, 2007; Buracas and Boynton 2007; Maunsell and Cook, 2002; Reynolds and Chelazzi, 2004*

3. Boghadi et al 2018 & 2019 describe a similar-seeming area, which they call FST/IPa, in macaques. The authors cite these studies as describing PITd, though the authors of the original studies seem to make an effort to focus on their particular region/nomenclature. Can the authors clearly state how the FST/IPa regions examined in these previous studies relate to the PITd region discussed/studied by the authors?

We thank the reviewer for pointing to the need of clarifications.

The investigations performed by Stemmann and Freiwald (2016) and Bogadhi and colleagues (2018; 2019) show remarkable similarities both in the experimental design and in their findings. They both performed an attentive motion detection task (Stemmann and Freiwald also performed a more demanding motion-discrimination task); they conducted a control task where moving dots were substituted by a different stimulus; they both described attentional activation in fundus/lower bank of the STS. The location of these activations has the same relative location from MT, as stated in (Bogadhi et al., 2018). Bogadhi et al., 2018 dedicate a paragraph to the comparison between their results and the results shown in Stemmann and Freiwald 2016. They concluded that the only difference is a discrepancy in the medial-lateral location of activation and that –*in their own words*- it "*might be explained by the difference in the retinotopic location of the stimuli used (8° in our*

study compared to 5° used in Stemmann et al. 2016". Importantly, despite the adopted nomenclature (FST/IPa), all their figures show activation at the intersection between three areas FST, IPa and PITd, i.e. the portion of TEO located in the lower bank of the STS (see for example Fig. 2 and 3 in Bogadhi et al., 2019). For the above reasons, we thought we could safely report that these studies are describing the same phenomenon.

We then decided to adopt a 'PIT nomenclature' for two important reasons: (1) it makes it clear that there is attentional activation outside the traditional motion-sensitive areas; FST instead is known to be strongly modulated by moving stimuli (Desimone and Ungerleider, 1986; Komatsu and Wurtz, 1988; Tanaka et al., 1993) (2) two recent reports (Kolster et al., 2010; Glasser et al., 2016) had already described evidence for functional similarities between human and macaque PIT and proposed a consistent nomenclature across species that would facilitate knowledge transfer across fields.

These labelling issues are not uncommon in brain function localization, as well as in cross-species comparisons especially when standard atlas localization is used. We indeed faced a similar challenge in the current study, where a fine distinction between Glasser areas phPIT and V8 could not be drawn. Overall, we believe that the multi-task functional characterizations performed in both Stemmann and Freiwald 2016 and Bogadhi et al., 2018, as well as in the current study, is the best tool do define the functional similarities across areas, studies and species.

4. The authors cite a separate report (Kolster et al, 2010) describing the retinotopic organization of area phPIT with respect to other retinotopic regions in the human visual system. However, there is a tremendous amount of variability between participants (this can be seen in the location of phPIT in the supplemental figures of the present study as well) – so ideally, the authors would be able to present the attentional task activations alongside at least a few individual participants' retinotopic maps. Given the current public health crisis, I imagine acquiring new human imaging data is impossible. I think this remains an important point to address, but will leave it to the editor(s) to determine whether this concern is justified.

We thank the reviewer for this request. We complimented the revised version with two additional experiments: retinotopic mapping and eccentricity mapping. We believe this improved the quality and precision of the current results. In the retinotopy experiment, for each participant we showed blocks of vertical and horizontal wedges of checkerboards. By contrasting these blocks we determined the boundaries between retinotopic visual areas. In the eccentricity experiment, we showed foveal and gradually more peripheral apertures (three in total) displaying checkerboard rings to map iso-eccentricity areas. Experimental details are described in the method section.

Results of this experiments are now illustrated in Supplementary figure 4 (also reported below). The combination of retinotopic and eccentricity mapping revealed to be a powerful tool to further separate infero-temporal activation from that of early visual areas, of motion areas, and of other specialized areas located more anteriorly in the temporal lobe. In individual subjects we were able to identify the attentional activation in the infero-temporal cortex as the most anterior and ventral region possessing a retinotopic organization, but not strong motion selectivity, nor complex object selectivity. This area corresponds for most subjects to Glasser areas phPIT and V8.

<u>Changes to the text:</u> *page 11 (main text), page 31-32 (method section), and page 56 (supplementary information) of the revised manuscript.*

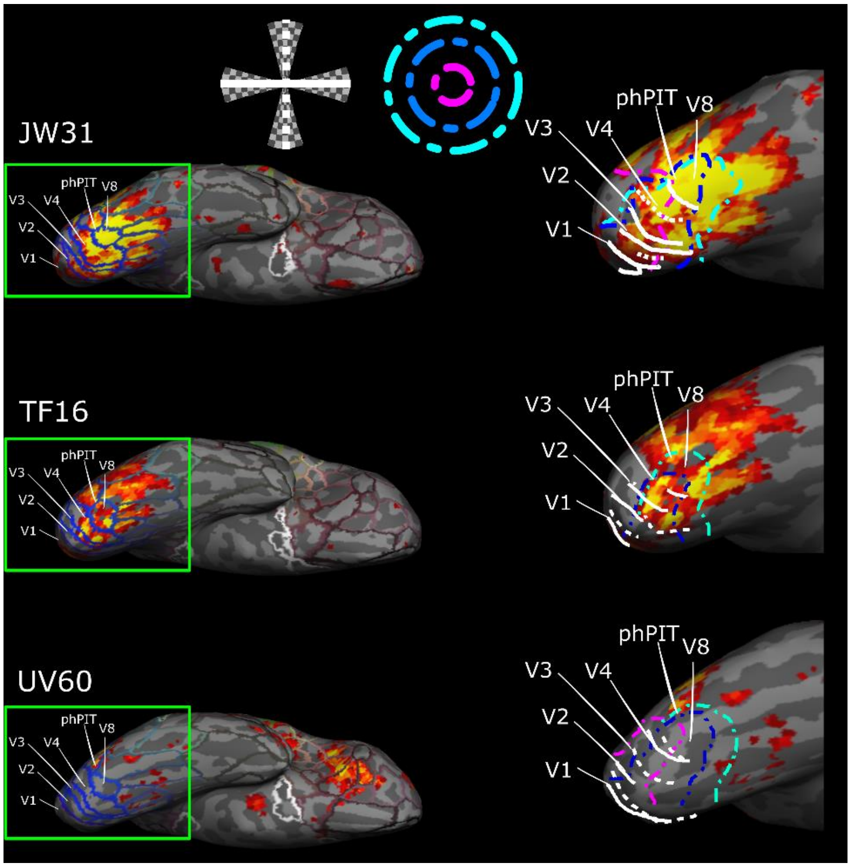

Supplementary Figure 4 – related to Figure 3. Individual subject retinotopic and eccentricity characterization of attentional activation phPIT. Statistical parametric maps of attention superimposed with meridians and eccentricity boundaries are overlaid on the inflated brain of two single subjects. Task-related activations (yellow/red) are shown on both lateral and inferior views and superimposed on the Glasser atlas parcellation (first column; (Glasser et al., 2016)) and with retinotopic and eccentricity mapping (second column – enlarged view). Full and dotted white lines indicate horizontal and vertical meridians respectively; colored dashed lines show positions of central, intermediate and peripheral eccentricity ridges. The combination of retinotopic and eccentricity mapping better clarified the separation between phPIT activation and that of early visual areas, of motion areas, and of other specialized areas located more anteriorly in the temporal lobe. In all subjects we were able to identify phPIT as the most anterior and ventral area possessing a retinotopic organization.

Minor comments:
5. I think the authors can/should more clearly delineate which aspects of the report come from their sample of participants (I understand the functional MRI data do), and which come from the HCP dataset. How are these interrelated? There are hints throughout the methods but I'm still not entirely sure I understand. Is there any reason they did not acquire diffusion data on this sample?

We thank the reviewer for pointing this problem out to us. We now more precisely explain in the methods section the provenance of the datasets used in the current study. In brief, all the fMRI data where acquired for the purpose of the current study; all the dMRI data were acquired by the HCP consortium. We interrelated the two datasets in three ways. First, we used the Glasser atlas as a reference for both datasets. This means that we localized attentional activation within the framework of the Glasser atlas and then used the very same atlas to parcel individual subjected from the HCP, thus drawing a direct correspondence between the location of the areas of interest (PIT, LIP, and FEF) in the two datasets. Secondly, we directly transferred attention ROIs defined in the fMRI experiment to the individual subject space of each HCP dataset and performed a control tractography. Thirdly, we utilized Talairach coordinates to draw spherical ROIs, which were likewise used to performance a control tractography analysis. As shown in Supplementary Figure 7, these three approaches gave consistent connectivity results. This approach is now highlighted in the methods.

Importantly, the Glasser atlas delineates brain areas on the basis of cortical architecture, function, connectivity, and/or topography, and thus provides a parcellation that takes into account individual differences. Our own localizers indeed showed a perfect agreement between the location of a given area (e.g. MT) parceled by the Glasser atlas and the actual location functionally defined for each individual subject (see Supplementary Figure 3). This striking observation, now further confirmed and strengthened by the retinotopic and eccentricity experiments, suggested we could use a high-resolution standardized dataset to draw faithful conclusion about the connectivity of PIT and other areas of interests.

The use of HCP dataset has important advantages: first, it gave us access to high quality data from many subjects; second, it allowed us to evaluate the statistical evidence supporting the existence of the fascicles of interest, a procedure that benefits from a big data analysis approach and that is particularly important in a field –*tractography*- where false positives are a matter of concern (the results of validation are shown in Figure 6E). Third, it strongly favored the development of a data processing pipeline fully accessible as computable applications and static code, which in turn guarantees the possibility to replicate, extend, and reuse the current results.

Changes to the text: *page 33-34-35 of the revised manuscript*

6. Functional data – alignment across subjects: for figures in which data is aggregated across subj (Figs 3 & 4), how is the data aligned? It seems as though analyses were conducted in SPM8 standard space after volumetric smoothing (implied on line 513). Lately it seems like aggregating data across subjects for visual system mapping is more effective using surface-based coregistration (e.g., using Freesurfer to warp surfaces together rather than volumes). I wonder if the authors have tried this? Indeed, for the single subject analyses (Supp Figs), it appears the analyses were performed/displayed in each subject's native surface space. More clarity here and throughout would help the reader understand the analyses performed.

We thank the reviewer and now clarify in the method section that group analyses were conducted in SPM8 standard space after volumetric smoothing. Single subject analyses were displayed in each subject's native surface space.

Changes to the text: *page 32-33 of the revised manuscript*

7. Fig. 3B: did the authors conduct 2 GLMs, one for each hemisphere? (e.g., for the LH, contrast attend right > attend left; for RH, attend left > attend right)? The different color bars for each hemisphere suggest this is the case. I'll also note that the title of this panel (ATTEND LEFT > ATTEND RIGHT) must be incorrect, or must only apply to the right side. Otherwise, we'd expect to see one hemisphere in cool colors and the other in warm colors.

We thank the reviewer and clarified it in the figure legend that 1 GLM for the whole brain was conducted and displayed in Figure 3B. The contrasts attention Right > Left and Left > Right are shown in the left and right column respectively, i.e. attention contralateral > ipsilateral. For visualization purposes, a slightly different threshold is displayed for the left and right hemisphere to highlight activation similarities. We thus promptly corrected the panel title to 'ATTEND CONTRALATEAL > ATTEND ISPILATERAL' and better explained the figure specifics in the capture.

Changes to the figure: *page 8-9 of the revised manuscript*

8. fMRI data presentation throughout is a bit confusing. In the methods (line 515), the authors mention using $p < 0.001$ FWE to define significant voxels. But it seems like different thresholds are used throughout – especially Fig. 3B and Fig. 4A/B. Moreover, I don't understand what's presented on the colorbars – T-values? Why isn't the full range of the colorbars used (e.g., no dark orange in Fig 3B)? And the colorbar in Fig. 4B doesn't show a lower bound for each direction.

We thank the reviewer for offering us an opportunity to better describe the methods and the figures. We used $p < 0.001$ FWE to define significant voxels in the attention task. For displaying purposes only, in figure 3B and 4A/B we used a different threshold in the two hemispheres. The color-bars represent T-values. Figure 3B shows the full range of the colorbars, however dark orange shades are only visible at the edges. Figure 4B doesn't show a lower bound for each direction because there is no significant activation for the null condition, which is a static visual stimulus in the motion localizer and objects, scenes, and scrambled images in the face localizer. In the revised version all the above details have been included in the figure captions.

<u>Changes to the text:</u> *pages 8-9 and 12-13 of the revised manuscript*

9. fMRI methods – the authors refer to their previous publication (Stremmann & Freiwald, 2016), but this is a single-subject fixed-effects report – typically random effects studies employ somewhat different procedures. The authors should add sufficient detail to understand how data was aggregated (& aligned, see above) across participants, and how statistics were performed.

We apologize for the lack of details provided in the original version of the manuscript. For consistency to the approach used in the macaques, the group analysis was performed as a fixed effects analysis concatenating all scanning sessions of all subjects together. In addition, the group analysis was also performed as a random effect analysis. The results were consistent with the two approaches and we now show both approaches in the main text (Fig. 3-4) and supplementary material (Fig. S2). Both types of group analyses were conducted in SPM8 standard space after volumetric smoothing. Single subject analyses were displayed in each subject's native surface space. ROI analyses were performed using a random effect approach using SPM8 and marsbar toolbox (as described above).

<u>Changes to the text:</u> *page 32-33 (method section) and page 51 (supplementary material) of the revised manuscript.*

10. Task – I'm a bit confused by the task design and stimuli. During the 'passive fixation' intervals described by the authors, is there still a RDK stimulus on the screen? (suggested by legend in Fig. 3A; Fig. 2B) Or, is there a blank fixation screen (as suggested by the first panel of Fig. 2A)? Moreover, when is the eye movement required to be made? During the stimulus presentation sequence after a target is detected? Perhaps the authors could show an example trial's eye trace measured during scanning beneath the event timing graph in Fig. 2B.

We thank the reviewer and more extensively describe the task in the methods section and figure legends by clearly separating a single trial description (Fig. 2A) and the block design (Fig. 2B and 3A).

The trial structure was consistent in each experimental condition: 'Attend left', 'Attend right', and 'Passive Fixation' (fig. 2A). Each single trial began with the fixation spot and the 8 peripheral targets. After 500 ms the cue appeared and instructed subjects to pay attention to the left or right hemifield. After additional 500 ms the RDS stimuli appeared. So in all trial type the moving stimuli were not on the screen for the first 1000 ms. After 1000 ms in all trial type two RDS appeared on the screen. In 'Passive Fixation' blocks the trial structure was exactly the same as attention trials, but no attentional cue was displayed and the subjects were therefore asked to maintain fixation on the central dot while passively viewing the RDS stimuli (fig.3A and 2B).

Subjects were required to maintain fixation until the prolonged event (i.e. a translation in direction of motion lasting 500 ms, instead of 60 ms) was detected and discriminated. At this point, subjects were expected to saccade towards the peripheral. Stimulus presentation, eye movements and behavioral monitoring were integrated online by custom written software. Therefore, whenever the subject broke fixation before the expected time the trial was aborted and considered as an early selection.

Following the reviewer suggestion, we now shown an example trial's eye trace measured during scanning beneath the trial timing graph in Fig. 2A to further clarify this point.

<u>Changes to the text:</u> *pages 7-8-9 and 30 of the revised manuscript*

11. Eyetracking – there are essentially no details provided for how eyes were tracked inside the scanner – this should be substantially elaborated, especially because it appears that the task timing was contingent upon correct behavioral performance (wait for 7 'hits' to move on to next block).

We apologize for the lack of details provided in the original version of the manuscript. We now substantially elaborate on the eye tracking approach used inside the scanner. The eye position was monitored at 60 Hz during all fMRI scanning sessions using a custom-made eye tracker system positioned at the back of the magnet to track pupil position and corneal reflection. During the attention task, subjects were required to maintain fixation until the prolonged event (i.e. a translation in direction of motion lasting 500 ms, instead of 60 ms) was detected and discriminated. At this point, subjects were expected/allowed to saccade towards the target matching the direction of motion of the random-dot stimulus to be attended, as detailed in the previous comment. Stimulus presentation, eye movements and

behavioral monitoring were integrated online by custom written software and a trial was aborted when subject broke fixation before the expected time.

Changes to the text: *page 29 of the revised manuscript*

12. Additional methods details: the authors should provide further details about (a) functional localizer tasks (stimulus size, duration, block properties, task, etc) – there's only 5 lines to account for at least 2 tasks now. Additionally, the reporting about how many runs are conducted is somewhat confusing – were 3 runs of the main task acquired per scanning session? Or across both sessions?

We acknowledge the reviewer concern about the brevity of the description of the localizers. We now added a paragraph in the methods section, with a detailed description of motion, face, object, scene localizers, as well as for the retinotopic and eccentricity mapping. We also more clearly report the number of runs per each task.

Changes to the text: *page 31-32 of the revised manuscript*

13. In Supplementary Fig. 2, I wonder why the authors see no object-selective voxels? I understand this is an example participant, but is this result common?

We acknowledge the reviewer catching this issue. In Supplementary Fig. 2 (bottom panels) of the original manuscript we erroneously showed the contrast scrambled vs. (face-object-scenes) instead of objects vs. (face-objects-scrambled). We now replaced those panels with the contrast showing object-selective voxels.

Changes to the text: Supplementary Figure 3, *pages 53 of the revised manuscript*

14. The three-node structure of the attention network the authors describe reminds me of a talk I recently saw by Randy Buckner, in which he described a 3-node (temporal/parietal/frontal) motif for resting state networks in humans and macaques. I haven't read it yet, but I believe this is all summarized in Buckner & DiNicola, 2019, Nature Reviews Neuroscience. I only mention this in case the authors might be interested in this framework – the similarity is somewhat striking though!

We thank the reviewer for raising this interesting point. The three-node structure of the attention network described here might be a general organizing principle shared across different networks, possibly emerging from evolutionary and developmental constrains, as

clearly explained by Buckner and DiNicola in their recent review. We now integrate in the discussion this critical point that would certainly speak to a broader readership.

Changes to the text: *page 26-27 of the revised manuscript*

Reviewer #4 (Remarks to the Author):

Sani et al: The human attentional control network includes a ventro-temporal cortical node

This is an interesting and ambitious study that present a novel idea. The authors' laboratory have previously argued that a region in the dorsal part of the posterior inferotemporal (PITd) cortex, in the ventral bank of the posterior superior temporal sulcus, with a role in exerting attentional control. This was an important observation because for many years it has been widely believed that the cortical centres exerting attentional control are in the parietal and frontal cortex. The authors build on their prior work in macaques and test the applicability of these ideas to the understanding of the human brain. Using the same cognitive task they identify a posterior temporal cortical region in the human brain with a similar activation profile. Second, they show that the topological relationship between that area and adjacent areas concerned with face processing and motion processing is similar in humans and macaques. Finally they show that the connectional anatomy of the human region, as inferred from diffusion weighted imaging, is similar to the connectional anatomy of the macaque PITd region.

The study is convincing as it currently stands. The following minor points might be considered in a revised version of the manuscript.

We thank the reviewer for highlighting the importance of the observation that attentional control is not only located in the parietal and frontal cortex, as well as for recognizing that the manuscript is convincing.

1 An argument is made that putative human PIT (phPITd) has activity that increases with attentional demand but not with visual motion per se. Would it be useful to show the visual motion onset related activity in the passive viewing condition in the phPITd region? This

might be done by extracting effects timelocked to motion onset versus some other trial time point from a region of interest in the phPIT.

We thank the reviewer for pointing to a possibility to further strengthen the idea that phPIT activity increases with attentional demand but not with visual motion per se. The blocked-design approach used in the current study prevented the time-sensitive analysis proposed by the reviewer. To further strengthen the point that activity in phPIT increases with attention but not with visual motion *per se*, we instead performed a detailed ROI analysis of phPIT and nearby regions (now included as Figure 4 of the revised manuscript). We defined ROI using the Glasser atlas. We refined phPIT definition by using information from an eccentricity and retinotopy mapping now also included in the revised manuscript (see method section, page 33 of the revised manuscript). We then quantified activity in each ROI to determine the profile of response of phPIT and compared it with that of traditional motion areas. MT, MST and FST were characterized by a strong response to moving stimuli and a non-significant response to static stimuli (Fig. 5B, revised manuscript). PIT, differently from motion areas and comparably to other inferior temporal areas, was similarly activated by moving and static stimuli (Fig. 5B, revised manuscript). Overall, the modulation and selectivity profile of area PIT stands out as the most attentionally activated, but does not stand out as the most motion selective (Fig. 5D). In both the attention task and motion localizers PIT modulation profile strongly differs from that of motion areas (in yellow) as well as from nearby temporal areas (in pink).

Changes to the text: *page 11-14 (main text) and page 33 (method section) of the revised manuscript.*

2 Diffusion weighted imaging is used to estimate connectivity between the phPIT and the lateral intraparietal area (LIP) in posterior parietal cortex and frontal eye field (FEF). The authors conclude that like macaque PIT, the phPIT has a high probability of connectivity with LIP and FEF. Would it be useful to supplement this demonstration with evidence of connections that both phPIT and macaque PITd lack? For example, while PITd in macaque is connected with LIP and FEF are there areas that adjacent brain regions are connected to but to which PITd is not connected? For example, does PITd have the same connections with extrastriate visual areas as MT+? If not, then is the same true of human phPIT? In other words, does phPIT have both the connections that resemble macaque PITd and does it lack the connections that macaque PITd lacks compared to its neighbours? An approach analogous to this one – examining both connections that ae present and connections that are absent – was used by Mars and colleagues (PNAS, 2013) when identifying a homologue of human TPJ in the macaque on the basis of MRI-derived estimates of connectivity.

Redacted

Redacted

3 Figure 7. I realise that this figure is diagrammatic and that every last details is not to be over interpreted. Nevertheless, human FEF is shown in two different locations in the panels on the left and right of figure 7. Is that intentional? On the left FEF is shown in a ventral location but on the right it is in a dorsal location. On the left it is the caudal bank of the precentral sulcus. On the right it is in the fundus of the precentral sulcus. The figure suggests that human precentral sulcus is one very big sulcus that spans the dorsoventral extent of the frontal lobe but, as I am sure the authors know, the human superior and inferior precentral sulci are usually separate sulci that do not join (Germann et al., J. Comp. Neurol., 2005).

We thank the reviewer for finding and alerting us on this imprecision in the summary figure. We now report the location of FEF, as segmented by Glasser and colleagues (2016) in the caudal bank of the superior component of the PS. FEF is now drawn at a consistent location in the right and left panels. We also more precisely draw the precentral sulcus with its superior and inferior components as reported by Germann et al., J. Comp. Neurol., 2005.

Changes to the figure: *page 27 of the revised manuscript.*

Reviewer #1 (Remarks to the Author):

This is an excellent work. I have no further comments and I recommend this paper for publication.

Reviewer #2 (Remarks to the Author):

The authors have satisfactorily addressed the points made in my review, and provided a good revision of their paper

Reviewer #3 (Remarks to the Author):

Overall, I'm pleased with the authors' careful response to my comments in this revision. I'm glad they were able to use retinotopic mapping data to help better understand how their phPIT region is anatomically situated among other well-characterized visual regions. I also am glad to see a figure showing the ROI-based analyses (new Fig. 5). I have two small requests the authors should be able to easily accommodate before I fully endorse this manuscript for publication.

1. Again, I appreciate the authors' inclusion of ROI analyses in the new Figure 5. However, I don't see any statistics reported associated with these analyses. If I'm understanding the expanded methods section correctly, these ROIs are based on the Glasser atlas, so it is not redundant to report statistics on extracted activation values. However, if I'm misunderstanding, or if the authors feel statistics are not appropriate or necessary to include here, I'm happy to hear their argument.
2. For the new Supplementary Figure 4 comparing attention-related activation to retinotopic region borders based on the authors' eccentricity/meridian scans, I think it's important to include at least a few examples of the actual retinotopic activation profiles – not just the meridians as included now.

Reviewer #4 (Remarks to the Author):

The authors have dealt with all the comments that I raised.

RESPONSE TO REVIEWERS

We are grateful to all Reviewers for their positive evaluation of our response. We would like to thank Reviewers #1, #2, and #4 for seeing the manuscript in its current form fit for publication and Reviewer #3 for two specific suggestions for improvement.

Below, we describe our point-by-point responses to the comments of Reviewer #3. All changes to the text of the manuscript have been marked in green to help the review process.

Reviewer #1 (Remarks to the Author):

This is an excellent work. I have no further comments and I recommend this paper for publication.

Reviewer #2 (Remarks to the Author):

The authors have satisfactorily addressed the points made in my review, and provided a good revision of their paper

Reviewer #4 (Remarks to the Author):

The authors have dealt with all the comments that I raised.

Reviewer #3 (Remarks to the Author):

Overall, I'm pleased with the authors' careful response to my comments in this revision. I'm glad they were able to use retinotopic mapping data to help better understand how their phPIT region is anatomically situated among other well-characterized visual regions. I also am glad to see a figure showing the ROI-based analyses (new Fig. 5). I have two small requests the authors should be able to easily accommodate before I fully endorse this manuscript for publication.

We thank Reviewers #3 for this positive evaluation of our response.

1. Again, I appreciate the authors' inclusion of ROI analyses in the new Figure 5. However, I don't see any statistics reported associated with these analyses. If I'm understanding the expanded methods section correctly, these ROIs are based on the Glasser atlas, so it is not

redundant to report statistics on extracted activation values. However, if I'm misunderstanding, or if the authors feel statistics are not appropriate or necessary to include here, I'm happy to hear their argument.

Following the reviewer suggestion, we now report in Figure 5 the statistics associated with the ROI analysis. Our ROIs were indeed based on the Glasser atlas, and this allowed us to perform the reviewer-requested analysis. To compare the two attentional conditions (attend ipsilateral vs. attend contralateral) and the two motions conditions (moving vs. static stimuli), we performed paired t-tests; to assess modulation exerted by different stimulus categories (faces, scenes or objects) we performed a one-way ANOVA. Results are shown below and reported in the revised manuscript.

The new Figure 5 shows that area phPIT is characterized by a strong significant attention effect. PIT, in fact, is the area with the largest attention index among areas with a robust positive response to visual stimuli (Fig. 5D, left panel) and the largest response difference of all areas for attended versus non-attended stimulus (Fig. 5D, left panel). Attention effect in PIT are thus stronger than in early visual areas or in motion areas (Fig. 5A).

Motion selectivity, exhibits almost the reverse pattern of effects. As the motion localizer confirmed, early visual areas, traditional motion areas MT, MST, FST, and even LO1,2, and 3 exhibit strong preferences for motion over static stimuli (Fig. 5B). Critically, traditional motion areas are characterized by a strong response to moving stimuli and a non-significant response to static stimuli. PIT exhibits a modulation by motion (Fig. 5B), which is smaller than that of the other areas relative to its visual activation (Fig. 5D), yet a strong activation by static stimuli (red asterisks). Finally, the shape localizer, confirmed specialized processing for faces in FFC, for places in PHA1-3, for objects in LO2. PIT, among the most strongly activated areas, showed instead similar responses for faces, scenes, and objects (Fig. 5C); the response differences between these categories were not significant.

Overall, PIT stood out as a highly and generally visually responsive area. PIT responds strongly to both shape-less motion stimuli and motion-less shape stimuli, suggesting that neurons in this area are not strongly tuned and thus capable of representing any stimulus. PIT also stood out as an area whose activity is very strongly attention modulated, both in absolute terms and relative to its degree of visual activation (Fig. 5D). Importantly, PIT's response profile across all three tasks strongly differs from that of nearby motion areas (in yellow) as well as from that of nearby temporal areas (in pink).

These confirmatory analysis is reported in main Figure 5 and the procedures are described in the method section.

Changes to the text: pages 14-15, page 33 and page 49 of the revised manuscript.

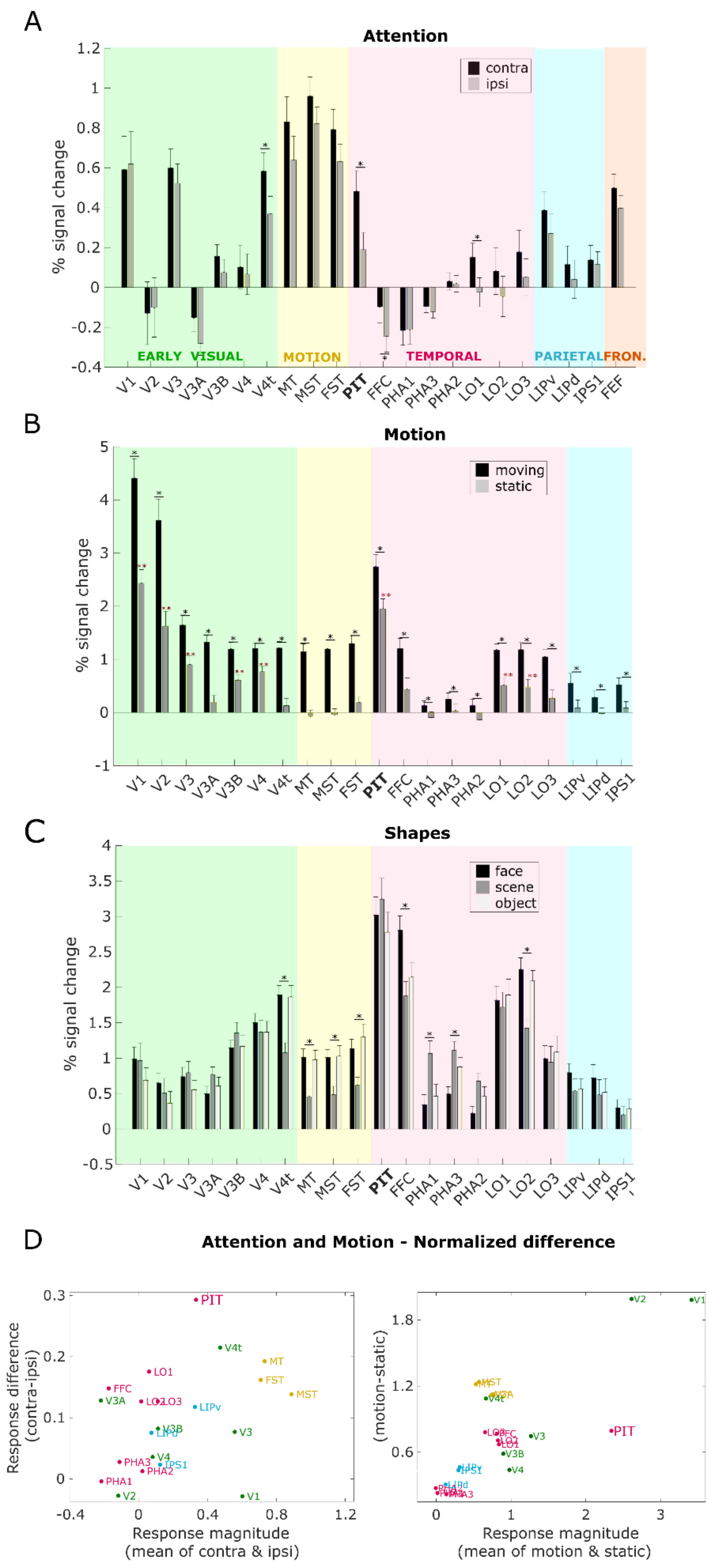

__Figure 5.__ Comparative functional profile of cortical ROIs in the occipital, temporal, parietal, and frontal lobes. __A.__ Attentional modulation; the bar plot shows the average percentage signal change across subjects for each ROI (error bars indicate across-subject standard error). Signal extracted during the attend contralateral (Attend contra) and the attend ipsilateral (Attend ipsi) condition are shown in black and gray respectively. Black asterisks indicate a significantly stronger response differences for attended than for unattended condition (p<0.05 non-corrected for Family Wise Errors - FWE); __B.__ ROI responses to motion and static stimuli; bar plots show the average percentage signal change across subjects for each ROI in response to moving and static stimuli; red asterisks indicate a significant response for static stimuli; black asterisks indicate a significantly stronger response differences for moving than for static stimuli (same conventions as in A). __C.__ ROI responses to three shape categories; bar plots show the average percentage signal change across subjects for each ROI in response to faces-scenes-objects; black asterisks indicate significantly different responses for the three different stimulus categories (same conventions as in A). __D.__ Attention and motion modulation profiles of ROIs relative to mean activation. Scatter plots show activation differences (vertical axis) as a function of average activation (horizontal axis) during the attention task (left) and the motion localizer (right). The ratio of response difference and response

magnitude defines the attention index.

	V1	V2	V3	V3A	V3B	V4	V4t	MT	MST	FST	**PIT**	FFC	PHA1	PHA2	PHA3	LO1	LO2	LO3	LIPv	LIPd	IPS1
**Attention**	0.54	0.54	0.28	0.11	0.08	0.36	**0.02**	0.06	0.17	0.08	**0.02**	**0.04**	**0.53**	0.23	0.43	**0.01**	0.06	0.14	0.19	0.29	0.34
**Motion**	**0.00**	**0.00**	**0.00**	**0.00**	**0.00**	**0.00**	**0.00**	**0.00**	**0.00**	**0.00**	**0.00**	**0.00**	**0.00**	**0.00**	**0.01**	**0.00**	**0.00**	**0.00**	**0.00**	**0.00**	**0.00**
**Static**	**0.00**	**0.00**	**0.00**	0.17	**0.00**	**0.00**	0.43	0.57	0.75	0.13	**0.00**	0.10	0.45	0.84	0.31	**0.00**	**0.01**	0.15	0.61	0.87	0.54
**Shapes**	0.63	0.63	0.58	0.37	0.61	0.83	**0.00**	**0.01**	**0.03**	**0.02**	0.62	**0.03**	**0.03**	**0.02**	0.09	0.89	**0.00**	0.92	0.55	0.76	0.88

Supplementary Table 2 – related to figure 5. *p-values for the statistics testing significance of response differences in the attention task (attended vs. unattended; paired t-test), motion localizer (moving vs. static stimuli; t-test), responsivity to static stimuli (t-test) and shape localizer (faces, scenes, objects; one-way ANOVA). Significant effects are indicated in bold. p-values < 0.01 are indicated as 0.*

2. For the new Supplementary Figure 4 comparing attention-related activation to retinotopic region borders based on the authors' eccentricity/meridian scans, I think it's important to include at least a few examples of the actual retinotopic activation profiles – not just the meridians as included now.

We followed the reviewer's suggestion and complimented Supplementary figure 4 with retinotopy and eccentricity maps for one representative subject. The new extended figure includes the maps for the vertical and horizontal meridian (two views each) and for the inner and outer ring (two views each). We report the new figure below as well as in the supplementary material.

Changes to the text: page 55-56 of the revised manuscript.

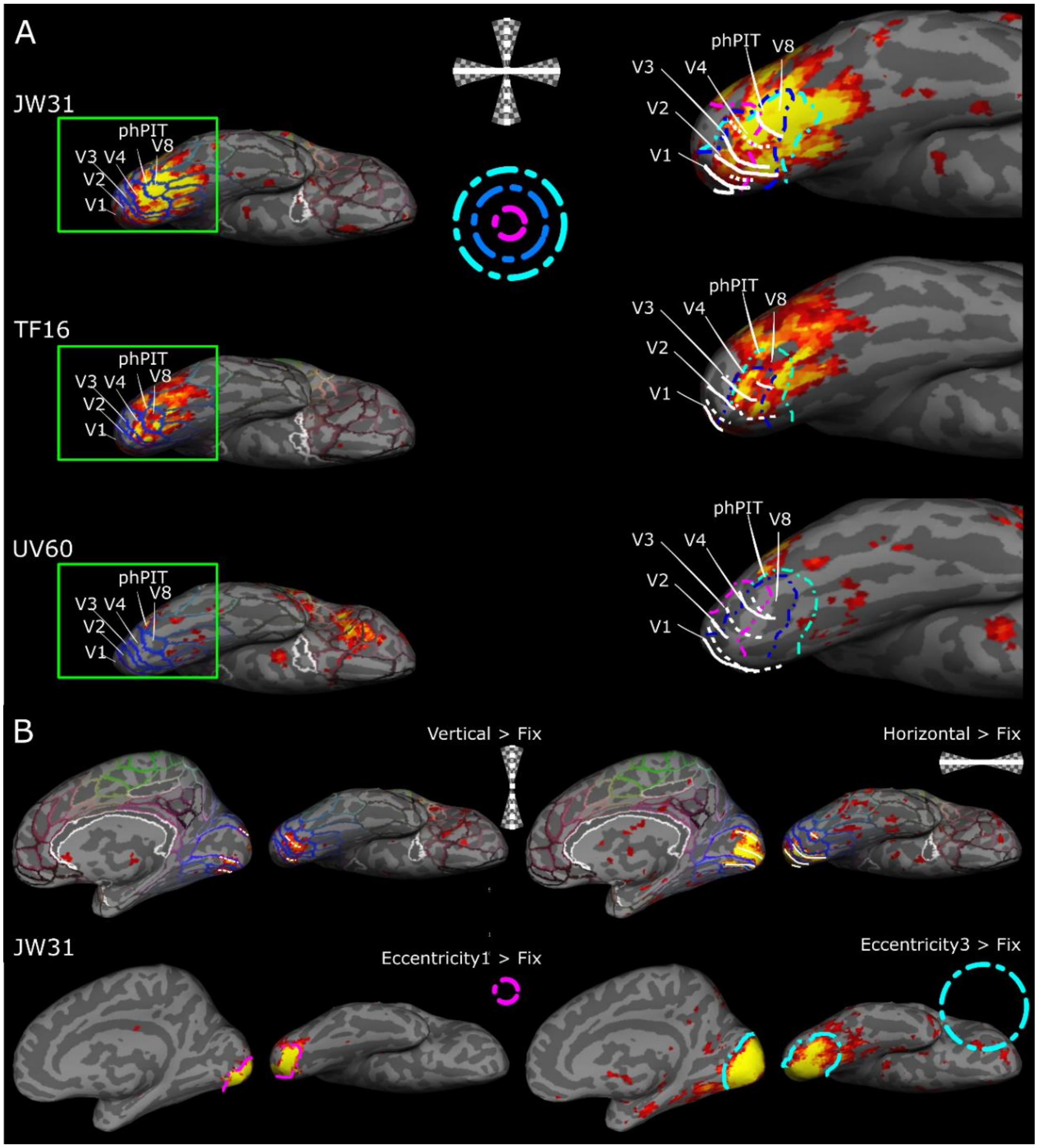

Supplementary Figure 4 – related to Figure 3*. Individual subject retinotopic and eccentricity characterization of attentional activation in phPIT.* ***A.*** *Statistical parametric maps of attention superimposed with meridians and eccentricity boundaries are overlaid on the inflated brain of three single subjects. Task-related activations (yellow/red) are shown on the inferior views and superimposed on the Glasser atlas parcellation (first column; (Glasser et al., 2016), 2016)) and with retinotopic and eccentricity mapping (second column – enlarged view). Full and dotted white lines indicate horizontal and vertical meridians respectively; colored dashed lines show positions of central, intermediate and peripheral eccentricity ridges. The combination of retinotopic and eccentricity mapping better clarified the separation between the infero-temporal activation and that of early visual areas, and of other specialized areas located more anteriorly in the temporal lobe. In individual subjects we were able to identify the attentional activation in the infero-temporal cortex as the most anterior and ventral region possessing a retinotopic organization, but not strong motion selectivity, nor complex object selectivity. In Glasser nomenclature phPIT corresponded to phPIT and V8 areas.* ***B.***

Statistical parametric maps of retinotopic (top, vertical and horizontal wedge) and eccentricity (bottom, inner and outer ring) for a representative subject.

Reviewer #3 (Remarks to the Author):

I appreciate the authors' careful attention to my comments throughout the review process, and I am happy to fully endorse the manuscript for publication.

(though the authors should double-check the sentence from Fig. 5's caption, copied below - do they mean 'uncorrected, $p < 0.05$', or 'corrected for multiple comparisons via family-wise error rate (FWE)'? The answer to this question will not impact my endorsement and this is only meant to help the authors correct their manuscript prior to publication)

sentence in question:
"Black asterisks indicate a significantly stronger response differences for attended than for unattended condition ($p<0.05$ non-corrected for Family Wise Errors - FWE)"

RESPONSE TO REVIEWERS – REBUT3

Reviewer #3 (Remarks to the Author):

I appreciate the authors' careful attention to my comments throughout the review process, and I am happy to fully endorse the manuscript for publication.

(though the authors should double-check the sentence from Fig. 5's caption, copied below - do they mean 'uncorrected, $p < 0.05$', or 'corrected for multiple comparisons via family-wise error rate (FWE)'? The answer to this question will not impact my endorsement and this is only meant to help the authors correct their manuscript prior to publication)

sentence in question:
"Black asterisks indicate a significantly stronger response differences for attended than for unattended condition ($p<0.05$ non-corrected for Family Wise Errors - FWE)"

We thank Reviewers #3 for his positive evaluation of our responses.

We promptly checked on Fig. 5‘s caption and clarify, both in the caption and in the method section, that our statistical tests were uncorrected for multiple comparisons. This was due to our focus on defining areal properties, rather than inter-areal comparison. More precisely, we aimed at characterizing PIT unique functional profile: PIT was strongly activated by attention and strongly activated by static stimuli, with a small motion modulation and similar responses for specific types of stimuli, namely faces, scenes, and objects.
